# Tuning the formal potential of ferrocyanide over a 2.1 V range†
†Electronic supplementary information (ESI) available: Materials and methods, additional data from NMR, X-ray crystallographic, laser-quench, and electrochemical experiments. CCDC 1877648, 1877649 and 1896046. For ESI and crystallographic data in CIF or other electronic format see DOI: 10.1039/c8sc04972f


**DOI:** 10.1039/c8sc04972f

**Published:** 2019-02-21

**Authors:** Brendon J. McNicholas, Robert H. Grubbs, Jay R. Winkler, Harry B. Gray, Emmanuelle Despagnet-Ayoub

**Affiliations:** a Beckman Institute , Division of Chemistry and Chemical Engineering , California Institute of Technology , 1200 East California Boulevard, Mail Code 139-74 , Pasadena , California 91125 , USA . Email: edespagnetay@oxy.edu; b Occidental College , Norris Hall of Chemistry , 1600 Campus Rd , Los Angeles , CA 90041 , USA

## Abstract

We report the synthesis and characterization of homoleptic borane adducts of hexacyanoferrate(ii). Borane coordination blueshifts d–d transitions and CN IR and Raman frequencies. Control over redox properties is established with respect to borane Lewis acidity, reflected in peak anodic potential shifts per borane of +250 mV for BPh_3_ and +350 mV for B(C_6_F_5_)_3_. Electron transfer from [Fe(CN-B(C_6_F_5_)_3_)_6_]^4–^ to photogenerated [Ru(2,2′-bipyridine)_3_]^3+^ is very rapid, consistent with voltammetry data. Coordination by Lewis acids provides an avenue for selective modification of the electronic structures and electrochemical properties of cyanometalates.

## Abstract 2

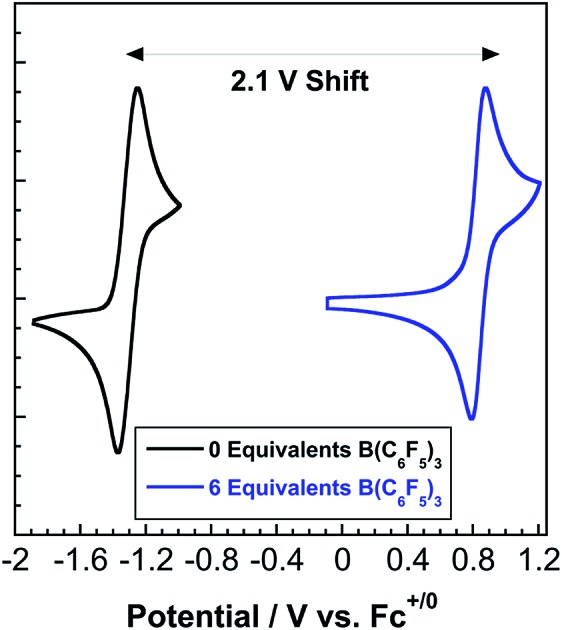
Coordination of tris(pentafluorophenyl)borane to hexacyanoferrate shifts the formal potential by over 2.1 V while maintaining electrochemical reversibility.

## Introduction

Duward Shriver pioneered the study of borane adducts of cyanometalates.1a–d Although he established from analysis of vibrational spectroscopic data that borane coordination (CN-BR_3_) enhanced M(d^6^)–CN π backbonding, surprisingly, to the best of our knowledge, no structures of d^6^ low-spin borane adducts have been hitherto reported.^2^ Indeed, only three homoleptic borane adducts have been crystallographically characterized, (TEA)_3_[Cr(NC-BPh_3_)_6_] (TEA = tetraethylammonium, BPh_3_ = triphenylborane), (CPh_3_)_2_[Ni(CN-B(C_6_F_5_)_3_)_4_] and (CPh_3_)_2_[Pd(CN-B(C_6_F_5_)_3_)_4_] (CPh_3_^+^ = trityl cation, B(C_6_F_5_)_3_ = tris(pentafluorophenyl)borane).^3^,^4^ Also surprising is that very little is known about the electrochemistry of coordinatively-saturated borane adducts of hexacyanometalates, with the majority of previous work focused on the solvent dependence of cyanometalate redox potentials.1d,^5^–^8^ We show herein that the formal potentials of these boronated adducts span an unusually wide range (over 2.1 V), providing a way to use as oxidants and reductants in energy storage devices.

## Results and discussion

Tetrabutylammonium (TBA) and TEA hexacyanoferrate(ii) compounds (**1a**, **1b**) were prepared using a modified literature procedure.^9^ Neutralization of H_4_[Fe(CN)_6_] with TBAOH (or TEAOH) in water generated the alkylammonium salt in quantitative yield. Bis(triphenylphosphine)iminium (PPN) hexacyanoferrate(ii) (**1c**) was prepared by combining four equivalents of PPNCl with one equivalent of K_4_[Fe(CN)_6_] in water. ^1^H NMR, ^13^C{^1^H} NMR, UV-vis, solid-state IR, and voltammetry confirmed the purity of **1**, with a single, reversible redox couple with a formal potential of –1.25 V *vs.* Fc^+/0^ in MeCN.

The borane adducts of **1** were synthesized by combining six equivalents of borane with one equivalent of **1** dissolved in dichloromethane (DCM) in a nitrogen-filled glove box. Coordination of borane generated (TEA)_4_[Fe(CN-BPh_3_)_6_] (**2**) and (PPN)_4_[Fe(CN-B(C_6_F_5_)_3_)_6_] (**3**). Each complex was purified and subsequently analyzed by ^11^B and ^13^C NMR spectroscopy, X-ray crystallography, UV-vis spectroscopy, elemental analysis, and voltammetry.

**Fig. 1:**
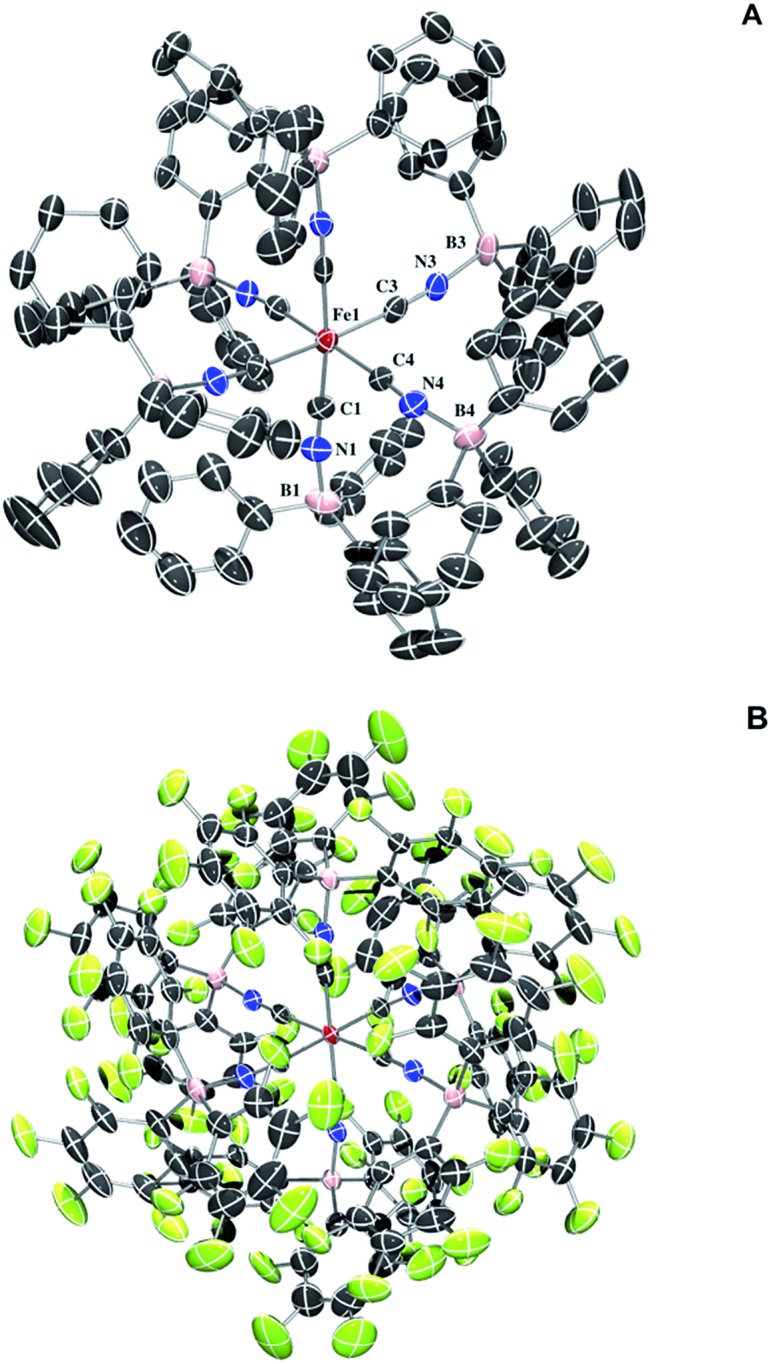
(A) Molecular structure of (TEA)_4_[Fe(CN-BPh_3_)_6_] (**2**). Thermal ellipsoids set at 50% probability. Cations and protons omitted for clarity. Selected bond distances and angles: Fe1–C1: 1.908(7) Å, Fe1–C3: 1.913(8) Å, Fe1–C4: 1.908(8) Å, C1–N1: 1.170(9) Å, C3–N3: 1.164(10) Å, C4–N4: 1.166(10) Å, N1–B1: 1.559(9) Å, N3–B3: 1.573(10) Å, N4–B4: 1.557(10) Å, Fe1–C1–N1: 174.0(7)°, Fe1–C3–N3: 174.0(7)°, Fe4–C4–N4: 179.6(8)°, C1–N1–B1: 172.0(8)°, C3–N3–B3: 173.5(8)°, C4–N4–B4: 176.3(7)°. (B) Molecular structure of (PPN)_4_[Fe(CN-B(C_6_F_5_)_3_)_6_] (**3**). Thermal ellipsoids set at 50% probability. Cations omitted for clarity. Selected bond distances and angles (labels as in **2**): Fe1–C1: 1.899(4) Å, Fe1–C3: 1.904(3) Å, Fe1–C4: 1.897(3) Å, C1–N1: 1.146(4) Å, C3–N3: 1.152(4) Å, C4–N4: 1.160(4) Å, N1–B1: 1.545(5) Å, N3–B3: 1.550(5) Å, N4–B4: 1.551(4) Å, Fe1–C1–N1: 176.9(3)°, Fe1–C3–N3: 177.9(3)°, Fe1–C4–N4: 175.9(3)°, C1–N1–B1: 173.2(3)°, C3–N3–B3: 175.1(3)°, C4–N4–B4: 173.3(4)°.

Full borane coordination for all species was confirmed by analysis of X-ray crystallographic data (see ESI†), with representative structures depicted in Fig. 1. The average M–CN (1.91 Å) and C–N (1.17 Å) bond lengths for **2** compared to **1**, 1.93 Å and 1.17 Å (Fig. S11†), respectively, are consistent with competing σ and π interactions, where σ donation from nitrogen to boron weakens the M–CN bond, while π backbonding strengthens it. Thus, the contraction of the M–CN bond in **2** is negligibly small. The average M–C–N (174.6°) and C–N–B (172.3°) bond angles in complex **2** are not perfectly linear, likely due to the effects of steric clash among the aryl groups. The average M–C–N (176.9°) and C–N–B (173.9°) bond angles for complex **3** are similarly perturbed. There are slight contractions in average M–CN (1.90 Å) and C–N (1.15 Å) bond lengths for **3** compared to those in complex **2**.

In the ^13^C{^1^H} NMR spectra, the cyanide carbon exhibits only one peak, indicating that boranes are bound to all six cyanides (*δ*_CN_: 159 ppm for **2** and 162 ppm for **3**). The carbon chemical shift follows the expected downfield trend for a Lewis acid inductively withdrawing electron density through the terminal nitrogen. The change in shift between the BPh_3_ and B(C_6_F_5_)_3_ adducts is small due to cooperative σ donation from the nitrogen and π backdonation from the metal center. It is well understood that isocyanoborate complexes experience decreased σ-bonding relative to cyano parents due to reduced electron density on carbon. Conversely, isocyanoborate complexes experience greater π-backbonding relative to cyanide complexes due to lower π*(CN) energies. The ^11^B NMR spectra of **2** and **3** are in line with increased electron-withdrawing by B(C_6_F_5_)_3_ relative to BPh_3_, with the ^11^B signal for B(C_6_F_5_)_3_ more upfield *versus* BPh_3_ (*δ* = –5.3 ppm for **2** and –14.4 ppm for **3**).

**Fig. 2:**
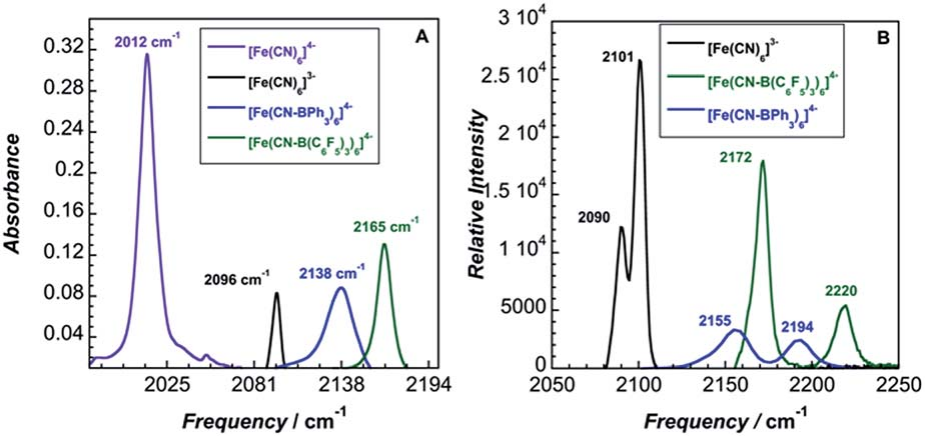
(A) Infrared spectra, (B) Raman spectra.

The solid-state IR and Raman spectra of **1** and its various adducts are shown in Fig. 2. As expected, the CN stretching frequency increases as the Lewis acidity of the borane or the oxidation state of the metal increases. Increased Lewis acidity causes increased stretching frequency, a result of lowering the absolute energies of the cyanide-based π-bonding orbitals.^9^

**Fig. 3:**
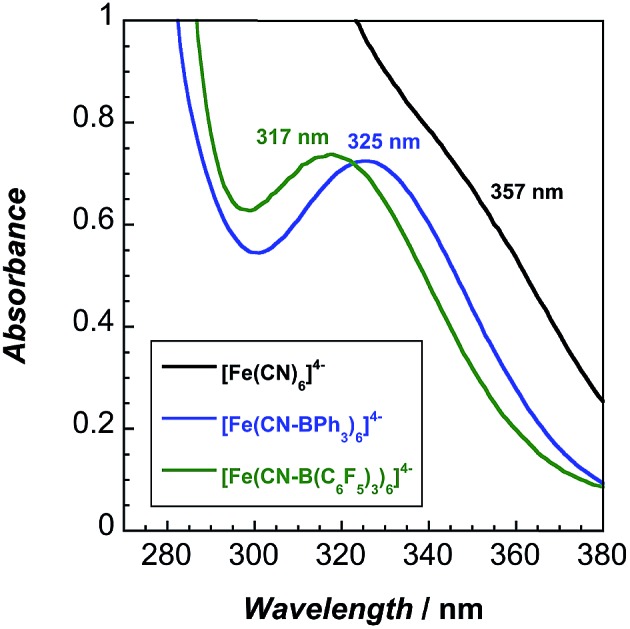
UV-vis absorption spectra of **1** and its borane adducts. Absorbance maxima (nm) and extinction coefficients (M^–1^ cm^–1^): **1b**: 357 (583), 255 (7204), 227 (10 130); **2**: 324 (347); **3**: 317 (333), 265 (9540), 230 (35 650).

The lowest energy absorption band in the UV-vis spectra (Fig. 3) of the adducts is attributable to a spin-allowed d–d transition (^1^A_1g_ → ^1^T_1g_).^10^ This band, 323 nm in water, redshifts to 357 nm in MeCN. Upon coordination of BPh_3_ and B(C_6_F_5_)_3_, **2** and **3** exhibit blueshifted absorbance maxima, indicating that increased backbonding outweighs decreased σ bonding in the octahedral ligand field.^10^ For **2** and **3**, bands below 270 nm are attributable to borane π to π* transitions (Fig. S16–S18†).

**Fig. 4:**
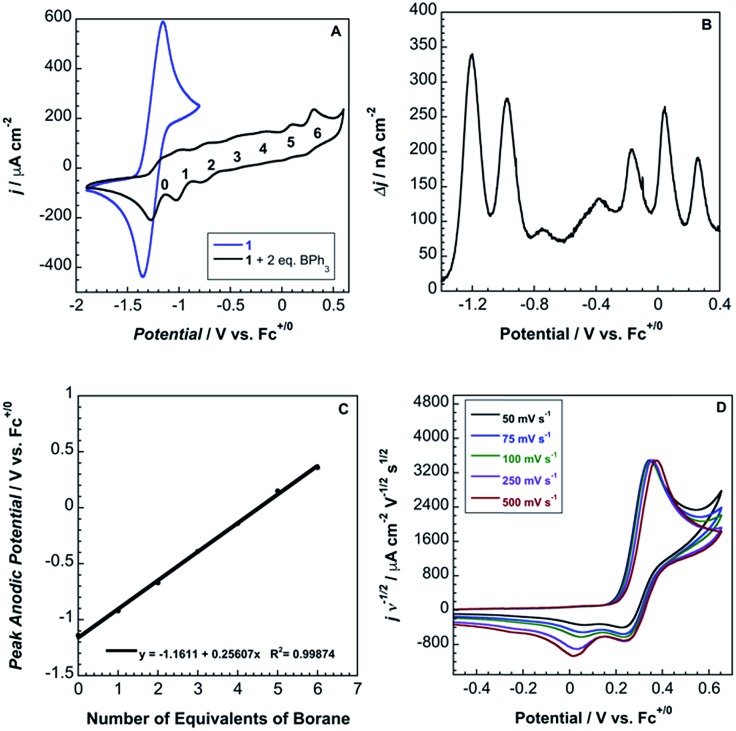
(A) Cyclic voltammograms of 5 mM **1b** (blue) and 5 mM **1b** with two equivalents of BPh_3_ (black) at 100 mV s^–1^ in DCM with 0.1 M TBAPF_6_. (B) Differential pulse voltammetry of **1b** with two equivalents of BPh_3_. (C) Linear regression of peak anodic potential from cyclic voltammetry *versus* number of BPh_3_ molecules coordinated to ferrocyanide. (D) Normalized scan-rate dependence of **2** in MeCN with 0.2 M TBAPF_6_.

Cyclic voltammograms of pure **1b** in DCM solution and one with two equivalents of BPh_3_ added are shown in Fig. 4A. Both CVs were corrected for the non-faradaic charging current and integrated to ensure that the anodic charge passed was equal for both, which suggests that the only redox reaction taking place is the one of interest (Fe^III/II^). By adding two equivalents of BPh_3_, seven anodic peaks are visible, corresponding to a distribution of all possible numbers of boranes coordinated to hexacyanoferrate(ii). Addition of a greater number of equivalents of borane increases the peak current for the coordinatively-saturated species (Fig. S7†). The seven anodic peaks corresponding to different coordination numbers are clearly seen in differential pulse voltammetry (Fig. 4B). The differential current responses for the two- and three-coordinate species are broader, likely due to the existence of isomers being oxidized at slightly different potentials, which decreases the intensity and broadens the observed differential wave for these adducts.^11^ As expected, the addition of borane results in a more positive peak anodic potential, likely due to lowering the absolute energies of the metal-based orbitals. The trend in peak anodic potential shift (from cyclic voltammetry) per borane added to hexacyanoferrate(ii) is linear (Fig. 4C), suggesting an electron withdrawing effect that is solely dependent on the Lewis acidity of the borane, with little to no attenuating effects as more boranes are added to the secondary coordination sphere.^12^ Assuming the peak anodic potential correlates with the formal potential for each borane species, the linear behavior is consistent with,1
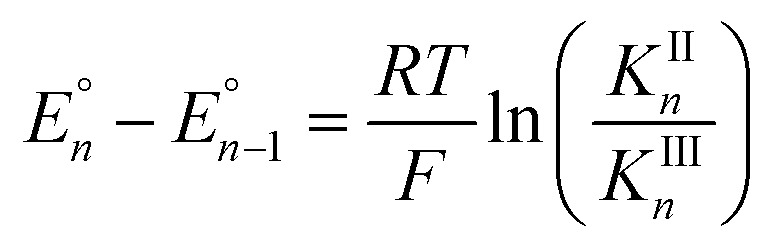
where the change in formal potential upon coordination of an additional borane, 
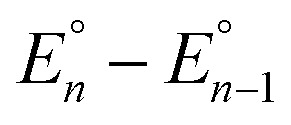
, is proportional to the ratio of binding constants of BPh_3_ to the Fe(ii) and Fe(iii) states of the complex, *K*II*n*/*K*III*n*.^12^ The approximate *E*_1/2_ value for **2** is 0.32 V *vs.* Fc^+/0^.

Although full coordination of borane in complex **2** was confirmed by both X-ray crystallography and ^13^C NMR, the voltammetry of **2** in both DCM and MeCN is not electrochemically reversible (Fig. 4D).

Cyclic voltammetry suggests that the complex undergoes electron transfer followed by a borane dissociation, with the metalloproduct undergoing reduction and subsequent re-oxidation (EC mechanism).^13^ The proposed mechanism is supported by a peak current ratio that is less than one for the six-coordinate species and by the presence of cathodic waves that correspond to reduction of the four-, five-, and six-coordinate species (Fig. 4A and D).^13^ We observe a 28 mV shift in peak anodic potential with a ten-fold increase in scan rate, consistent with the theoretical value of 30 mV for a purely kinetic EC mechanism.^13^ This mechanism also is supported by the appearance of anodic waves for the four- and five-coordinate species as a result of sweeping through multiple cycles at fast scan rates and by the decrease in anodic current for the six-coordinate species after the first scan (Fig. S8†). The peak cathodic current does not increase with increasing scan rate, consistent with very rapid BPh_3_ dissociation.^13^ Additionally, **2** was oxidized with dibenzo-1,4-dioxin radical cation in MeCN, and ^1^H NMR in CD_3_CN confirmed the presence of Ph_3_B–NCMe (Fig. S9†).

**Fig. 5:**
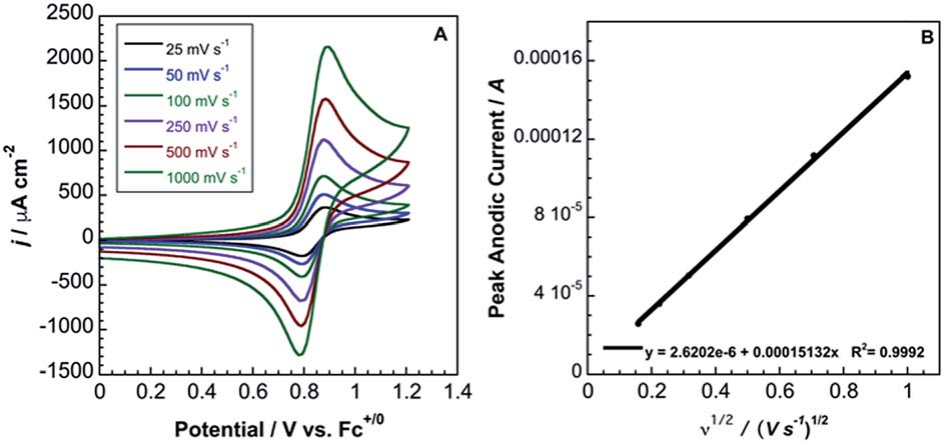
(A) Cyclic voltammetry (scan rates from 25 mV s^–1^ to 1000 mV s^–1^) of 3.6 mM **3** in acetonitrile with 0.2 M TBAPF_6_. Potentials relative to Fc^+/0^. (B) Randles–Sevcik plot of the CV data from (A).

In contrast to **2**, complex **3** displays a single, electrochemically reversible redox event with a formal potential of 0.85 V *vs.* Fc^+/0^ (Fig. 5A), corresponding to a 2.1 V anodic shift in the Fe^III/II^ couple, which is a 350 mV anodic shift per B(C_6_F_5_)_3_ added to hexacyanoferrate(ii). Similar to **2**, a linear potential shift per borane added to hexacyanoferrate(ii) was observed for **3** (Fig. S10†). Borane adducts of Fe(phen)_2_(CN)_2_ in dichloromethane showed an approximately 300 mV increase in peak anodic potential per borane, with BBr_3_ producing the largest shift.1*d* Cyanorhenate(i) complexes, Re(R_2_phen)(CO)_3_[CN-B(C_6_F_5_)_3_], where R = H, Me, displayed a ∼320 mV shift in peak anodic potential (*E*_p,a_) in acetonitrile compared to the parent.^14^ Cyanoosmate(ii) complexes, [Os(4,4′-R_2_(bpy))_2_(CNBL_3_)_2_], where R = H, Me, showed ∼420 and ∼290 mV anodic shifts per borane for L = (C_6_F_5_)_3_B and BPh_3_ in MeCN, respectively.^15^ As the Fe^III/II^ peak anodic potential shifts are very near those observed for Re(i) and Os(ii) complexes, there is minimal dependence on metal identity or oxidation state.

The peak current, *i*_p_, of an electrochemically reversible, diffusion-controlled voltammogram is defined by,^16^2
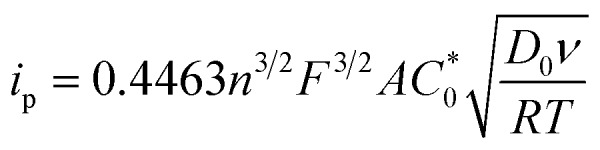
where *n* is the number of electrons, *F* is Faraday's constant, *D*_0_ is the diffusion coefficient (cm^2^ s^–1^), *ν* is the scan rate (V s^–1^), *A* is the surface area of the electrode (*A* = 0.0707 cm^2^) and 
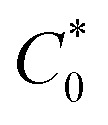
 is the bulk concentration of the redox-active species (mol cm^–3^). The diffusion coefficient for **3** in MeCN, obtained from a Randles–Sevcik plot of peak current *vs. ν*^1/2^, was found to be 4.9 × 10^–6^ cm^2^ s^–1^, which is similar to values for potassium ferrocyanide in aqueous electrolyte.^17^,^18^

Oxidation of **3** by flash-quench-generated [Ru(2,2′-bipyridine)_3_]^3+^ was very rapid. From a linear fit of electron transfer rate *versus* concentration (Fig. S15†), the apparent second order rate constant, *k*_ex_, was found to be 8.4 × 10^8^ M^–1^ s^–1^. Using an ion-pair preequilibrium model, *K*_0_, the ion-pair association constant, was found to be 0.19 M^–1^, with *k*_et_ estimated to be 4.5 × 10^9^ s^–1^.^19^ We conclude that electron tunneling from Fe^II^ to Ru^III^ in the ion-paired precursor complex is not inhibited by the wall of 90 fluorine atoms in the boronated cyanide complex.

## Conclusions

We have demonstrated that boranes can tune the formal potentials of reversible redox couples. This finding means that researchers can selectively alter the formal potentials of cyanide-based inorganic complexes, providing opportunities for creating new quenchers, oxidants, and single-electron transfer reagents. Importantly, the fluorinated “cage” surrounding the iron center of **3** does not hinder outer-sphere electron transfer, indicating that boronated cyanide complexes will likely be useful as electrolytes in non-aqueous redox flow batteries.^20^

## Conflicts of interest

There are no conflicts to declare.

## Supplementary Material

Supplementary information

Crystal structure data
