# Supplementary material for: Tuning the formal potential of ferrocyanide over a 2.1 V range
Source: Chem Sci. 2019 Feb 21;10(12):3623–6. doi: 10.1039/c8sc04972f (PMC6430091; doi:10.1039/c8sc04972f)
Supplement: Supplementary file 1 [file SC-010-C8SC04972F-s001.pdf]

Electronic Supplementary Material (ESI) for Chemical Science.
This journal is © The Royal Society of Chemistry 2019

Tuning the Formal Potential of Ferrocyanide over a 2.1 V Range

Brendon J. McNicholas, Robert H. Grubbs, Jay R. Winkler, Harry B. Gray*, and Emmanuelle Despagnet-Ayoub*

Beckman Institute, and Division of Chemistry and Chemical Engineering, California Institute of Technology, 1200 East California Boulevard, Mail Code 139-74, Pasadena, California 91125, United States

Occidental College, Norris Hall of Chemistry, 1600 Campus Rd., Los Angeles, CA, 90041, United States

Contents:

1. **Synthesis**
2. **Electrochemical Measurements**
3. **Crystallographic Data**
4. **Laser Experiments**
5. **UV-Vis Data**

1. Synthesis

General:

Tetrabutylammonium hydroxide (Sigma-Aldrich), tetraethylammonium hydroxide (Sigma-Aldrich), potassium hexacyanoferrate(II) (Sigma-Aldrich), bis(triphenylphosphine)iminium chloride (Sigma-Aldrich), methyl triflate (Sigma-Aldrich), tris(pentafluorophenyl)borane (Sigma-Aldrich), and triphenylborane (Sigma-Aldrich) were used as received. [dibenzo-1,4-dioxin][$SbCl_6$] was prepared according to literature precedent.[1] NMR spectra were collected on either a Varian 400 MHz spectrometer or Bruker 400 MHz spectrometer (*δ* in ppm, m: multiplet, s: singlet, d: doublet, t: triplet, pt: pseudo-triplet). ^{13}C NMR and ^{31}P NMR were ^{1}H decoupled. ^{31}P NMR spectra were externally referenced to 85% H_3PO_4, ^{19}F NMR spectra were externally referenced to neat $CFCl_3$, and ^{11}B NMR were internally referenced to 15% $BF_3{\cdot}Et_2O$. UV-visible spectroscopy were collected on an HP 8453 spectrometer. Solid-state infrared spectra were collected on a Thermo Scientific Nicolet iS5 FT-IR spectrometer with an iD5 ATR diamond. Solid-state Raman spectra were collected using a coherent Innova argon ion laser with a 488 nm line, a Spex 0.75 m spectrograph, and a Horiba thermoelectrically cooled CCD detector.

Tetrabutylammonium/tetraethylammonium hexacyanoferrate(II), $(TBA)_4/(TEA)_4[Fe(CN)_6]$ (1a/1b).

$H_4[Fe(CN)_6]$ was prepared according to literature procedure.[2] $H_4[Fe(CN)_6]$ (0.25 g, 1.16 mmol) was combined under nitrogen with a stoichiometric excess of 55% tetrabutylammonium hydroxide (TBAOH) in water (2.2 mL, 1.20 g, 4.64 mmol). The reaction was stirred for one hour and then immediately dried under vacuum. The product was then placed in a nitrogen-filled glove box, washed with ether to remove excess TBAOH, and dried under vacuum. To remove trace water, the desired product was repeatedly redissolved in dicholoromethane and re-dried to obtain a colorless powder. UV-visible spectroscopy can be used to confirm the absence of ferricyanide (Fe(III)). **1a** ^{1}H NMR (400 MHz, CD_3CN) δ 2.94 (br s, 8H, CH_2), 1.56 (br s, 8H, CH_2), 1.31 (br s, 8H, CH_2), 0.95 (br s, 12H, CH_3); ^{13}C NMR (400 MHz, CD_3CN) δ 144.7 (CN) δ 59.7 (s, CH_2), 24.4 (s, CH_2), 20.4 (s, CH_2), 13.9 (s, CH_3). **1b** ^{1}H NMR (400 MHz, CD_3CN) δ 3.28 (q, 8H, CH_2), 1.20 (t, 12H, CH_3); ^{13}C NMR (400 MHz, CD_3CN) δ 143.4 (CN) δ 52.9 (s, CH_2), 7.9 (s, CH_3).

Bis(triphenylphosphine)iminium hexacyanoferrate(II), $(PPN)_4[Fe(CN)_6]$ (1c).

PPNCl (6.00 g, 0.010 mmol) was dissolved in approximately 3 L of water. $K_4[Fe(CN)_6]$ (0.822 g, 0.0022 mmol) was added, and a colorless precipitate immediately formed. The solution was filtered, and the precipitate was washed three times with water. The precipitate was placed on a vacuum line and dried and immediately transferred to a nitrogen-filled glove box and redissolved in dichloromethane to remove trace water. The solution was dried, washed with ether to remove any remaining PPNCl, and re-dried to obtain a colorless powder. ^{31}P NMR (162 MHz, CD_3CN) δ 20.8; ^{1}H NMR (400 MHz, CD_3CN) δ 7.45-7.67 (m, 30H, CH_{Ph}); ^{13}C NMR (100 MHz, CD_3CN) δ 134.5 (m), 133.2 (m), 130.3 (m), 128.7 (dd, J_{CP} = 108 Hz, J_{CP} = 2 Hz), the CN peak was not observed.

$(TEA)_4[Fe(CN\text{-}B(C_6H_5)_3)_6$ (2). Triphenylborane (BPh_3) was purified by washing repeatedly with diethyl ether to remove a yellow impurity until the washes and the borane were colorless. The purity of the BPh_3 was confirmed by ^{11}B NMR. $(TEA)_4[Fe(CN)_6]$ (0.20 g, 0.27 mmol) was combined with 6.1 equivalents of BPh_3 (0.40 g, 1.65 mmol) in DCM (5 mL). After one hour of stirring, the precipitate was filtered and washed with ether. The desired product was obtained as a white powder (0.4 g, 67 %). Elem. Anal. (%) found (calc'd) for $C_{146}H_{170}B_6FeN_{10}$ ($(TEA)_4[Fe(CN\text{-}B(C_6H_5)_3)_6]$): C, 78.67 (80.27); H, 7.71 (8.14); N, 6.88 (6.46). ^{11}B NMR (128 MHz, CD_3CN) δ -5.3; ^{1}H NMR (400 MHz, CD_3CN) δ 7.45 (d, J_{HH} = 7.9 Hz, J_{HH} = 1.5 Hz, 36H, CH_{Ph}), 6.53 (t, J_{HH} = 7.3 Hz, 18H, CH_{Ph}), 6.35 (t, J_{HH} = 7.5 Hz, 36H, CH_{Ph}), 3.08 (q, J_{HH} = 7.4 Hz, 32H, CH_2), 1.16 (t, J_{HH} = 7.4 Hz, 48H, CH_3) ^{13}C NMR (100 MHz, CD_3CN) 134.4 (s, CH), 127.0 (s, CH), 123.1 (s, CH), 53.0 (s, CH_2), 7.7 (s, CH_3).

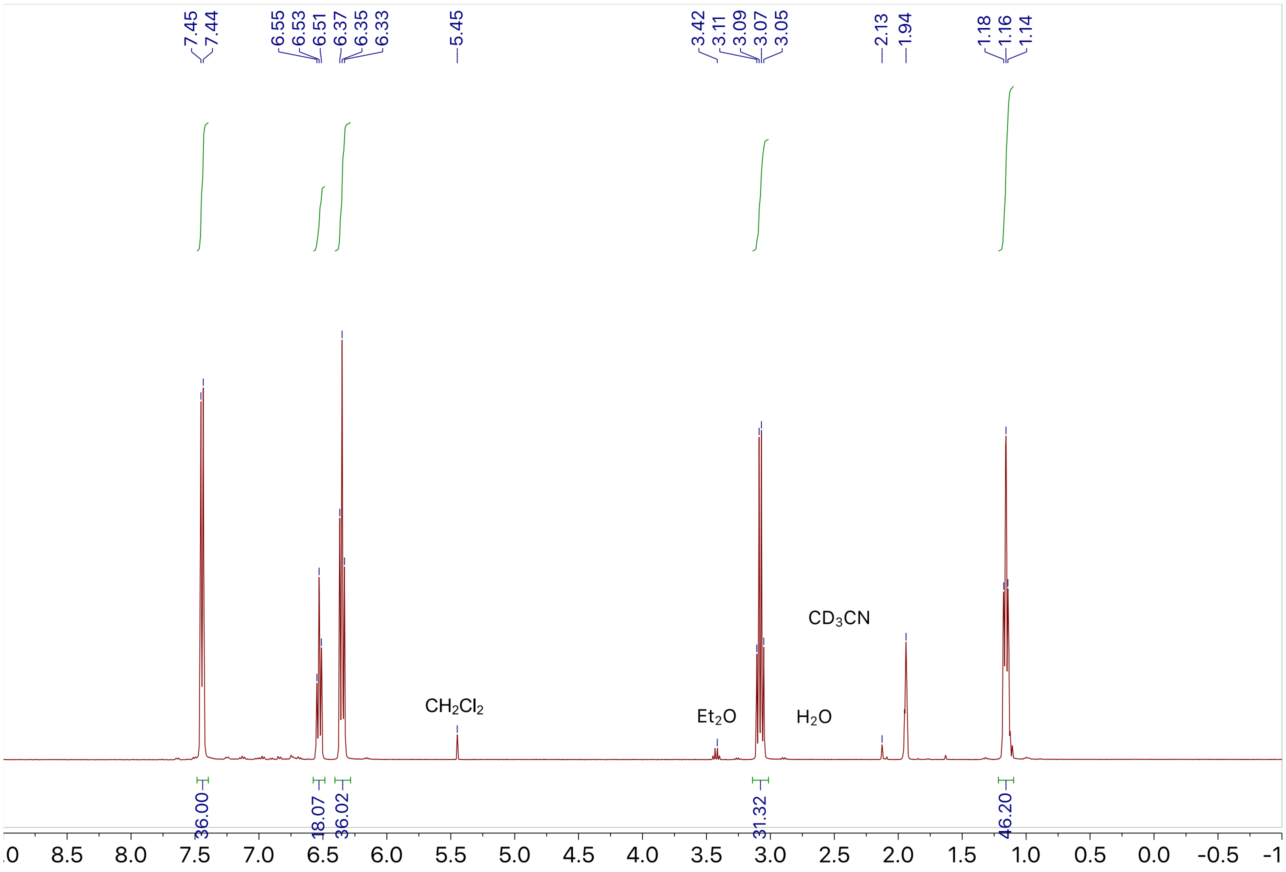

Figure S1. ^{1}H NMR of complex **2** in MeCN-d3.

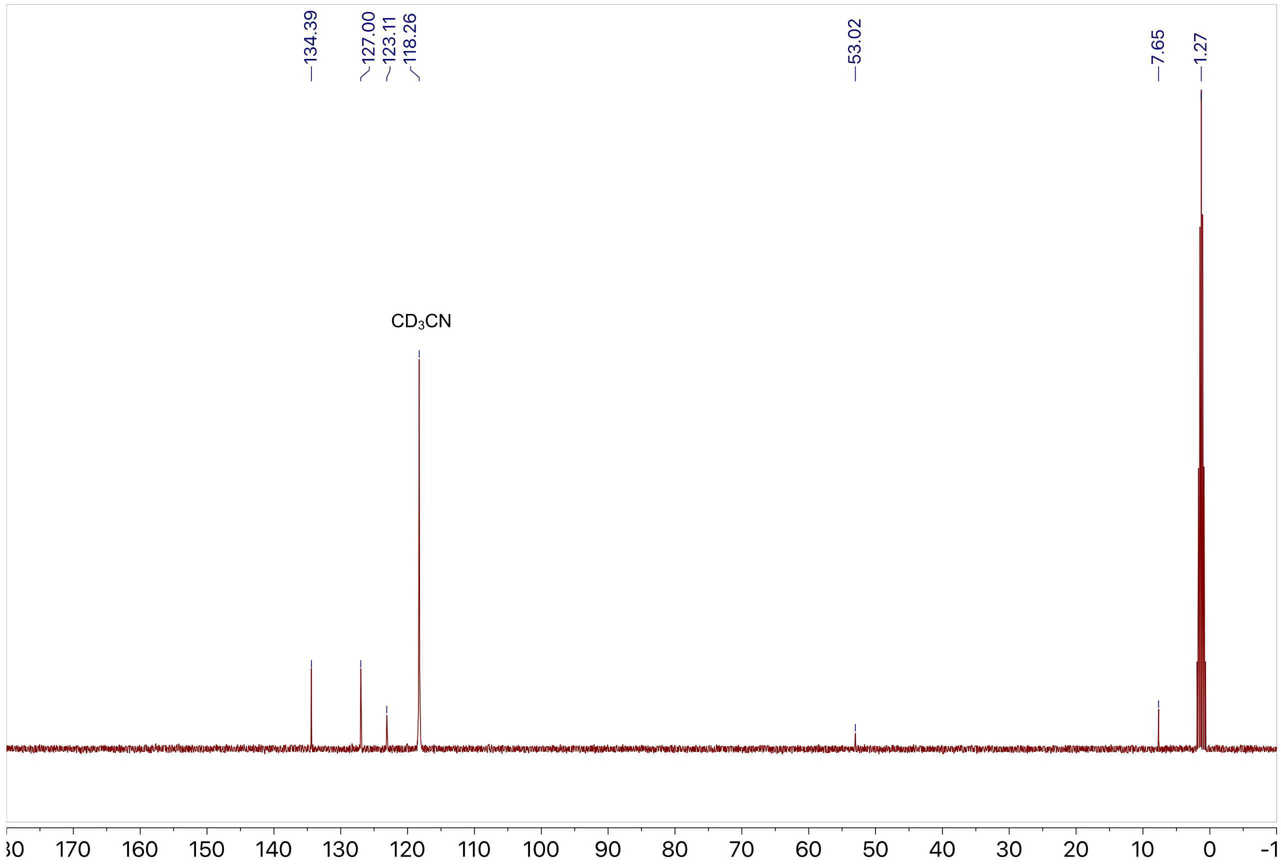

Figure S2. ^{13}C NMR of complex **2** in MeCN-d3.

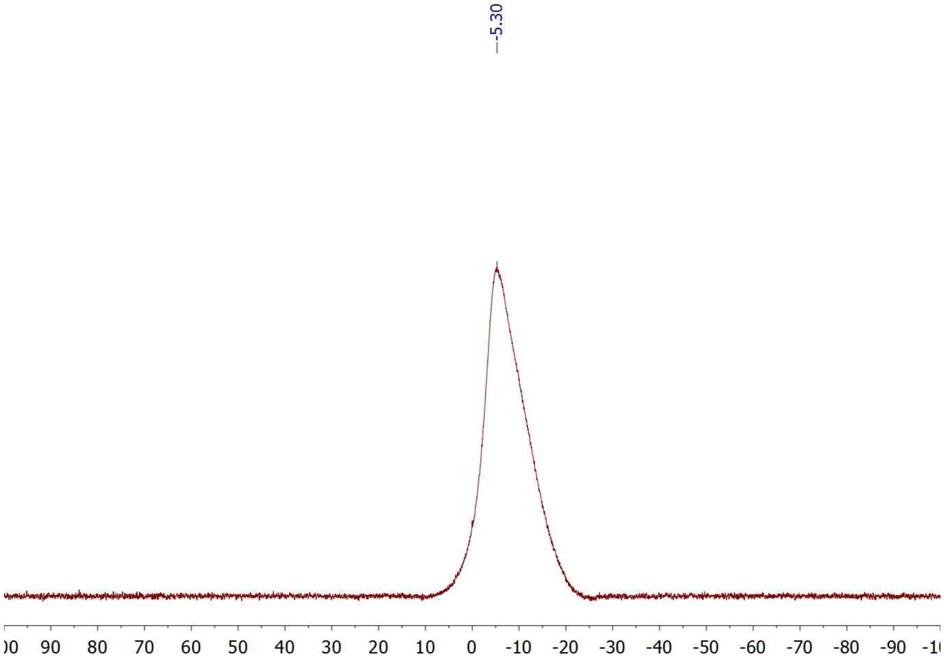

Figure S3. ^{11}B NMR of complex **2** in MeCN-d3.

$(PPN)_4[Fe(CN\text{-}B(C_6F_5)_3)_6]$ (3). $(PPN)_4[Fe(CN)_6]$ (0.45 g, 0.19 mmol) was combined with 6.2 equivalents of tris(pentafluorophenyl)borane (0.6 g, 1.17 mmol) in DCM (10 mL). After 30 minutes, ether was added to the reaction mixture and the vial was stored in the fridge yielding a white powder. The resulting precipitate was filtered and washed with ether. For further purification, the precipitate was dissolved in tetrahydrofuran and precipitated with ether. After drying under vacuum, the desired product was obtained as a white powder (0.62 g, 0.11 mmol, 57 %). The tetrabutylammonium salt of this compound was synthesized in an identical manner. Elem. Anal. (for TBA salt) (%) found (calc'd) for $C_{178}H_{144}B_6F_{90}FeN_{10}$ ($(TBA)_4[Fe(CN\text{-}B(C_6F_5)_3)_6]$): C, 50.18 (50.26); H, 3.67 (3.41); N, 3.28 (3.29). ^{11}B NMR (128 MHz, CD_3CN) δ -14.4; ^{31}P NMR (162 MHz, CD_3CN) δ 20.8; ^{19}F NMR (376 MHz, CD_3CN) δ -132.3 (d, J_{FF} = 20.7 Hz), -165.0 (pt, J_{FF} = 19.8 Hz), -169.0 (pt, J_{FF} = 18.1 Hz); ^{1}H NMR (400 MHz, CD_3CN) δ 7.43-7.66 (m, 30H, CH_{Ph}); ^{13}C NMR (100 MHz, CD_3CN) δ 161.5 (CN), 149.1 (d, J_{CF} = 242 Hz), 139.5 (d, J_{CF} = 245 Hz), 136.7 (d, J_{CF} = 248 Hz), 134.5 (s), 133.4 (m), 130.3 (m), 128.1 (dd, J_{CP} = 108 Hz, J_{CP} = 2 Hz), 122.1 (br. s, CB).

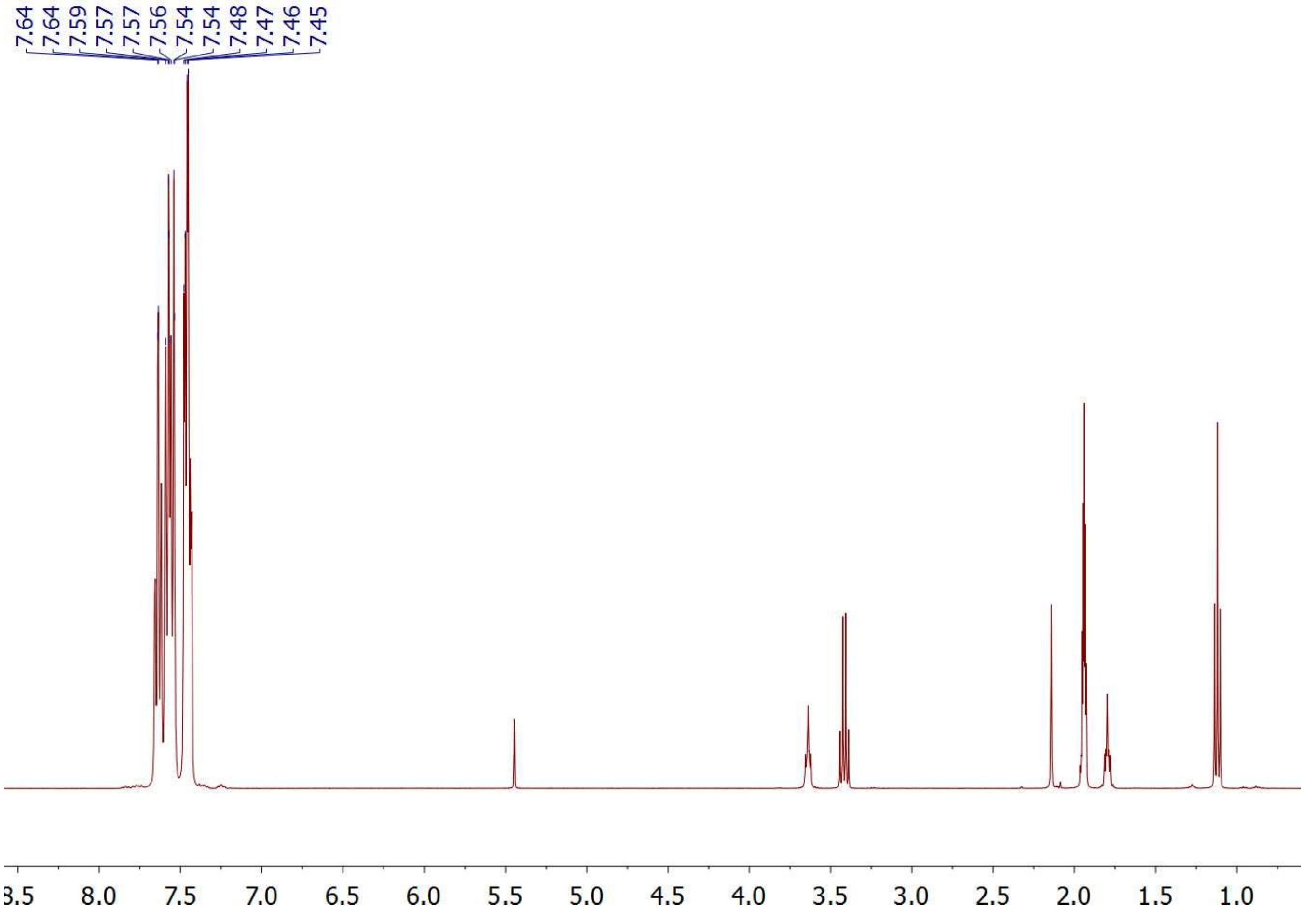

Figure S4. ^{1}H NMR of complex **3** in MeCN-d3 (presence of Et_2O, CH_2Cl_2, THF, and H_2O).

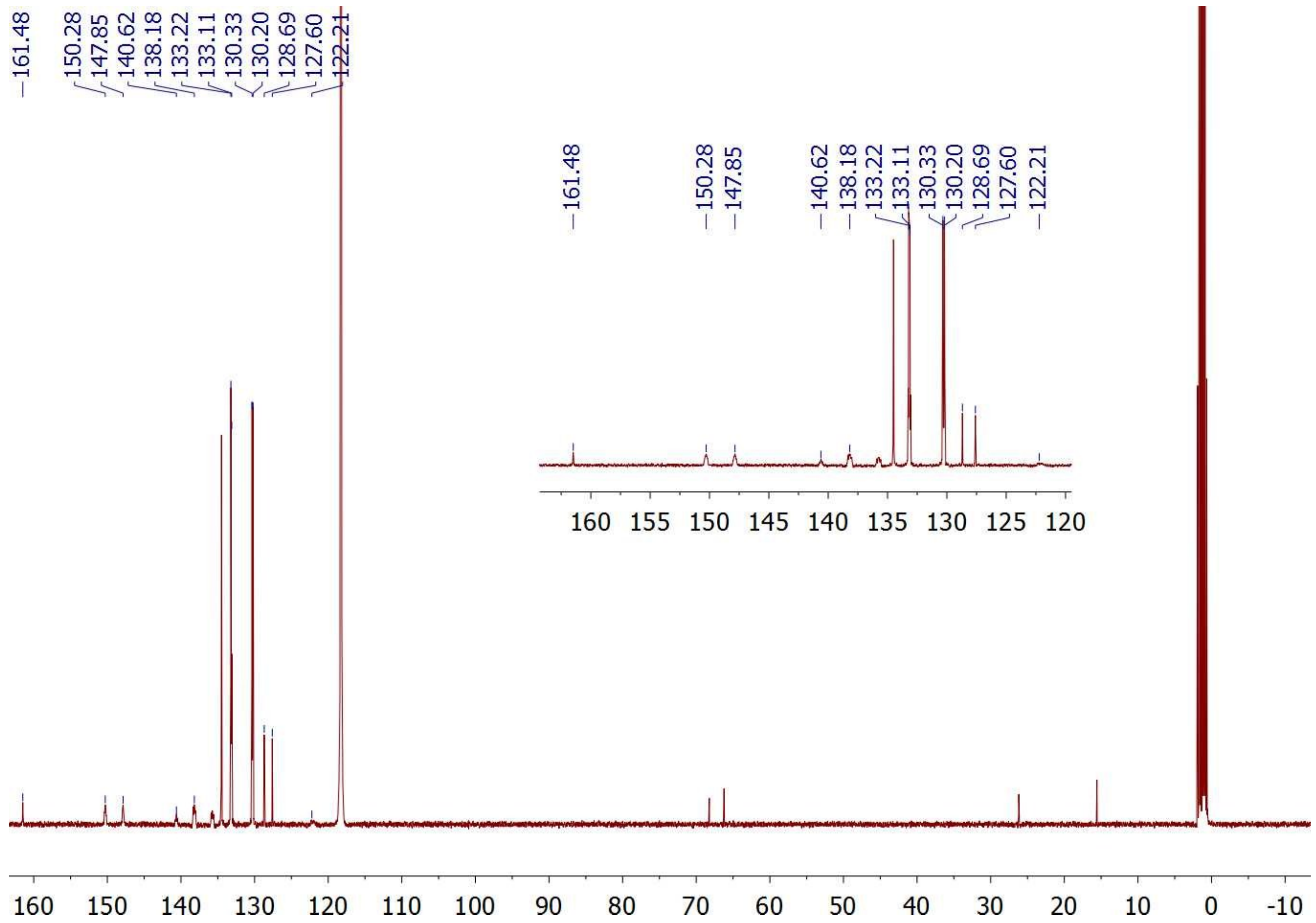

Figure S5. ^{13}C NMR of complex **3** in MeCN-d3.

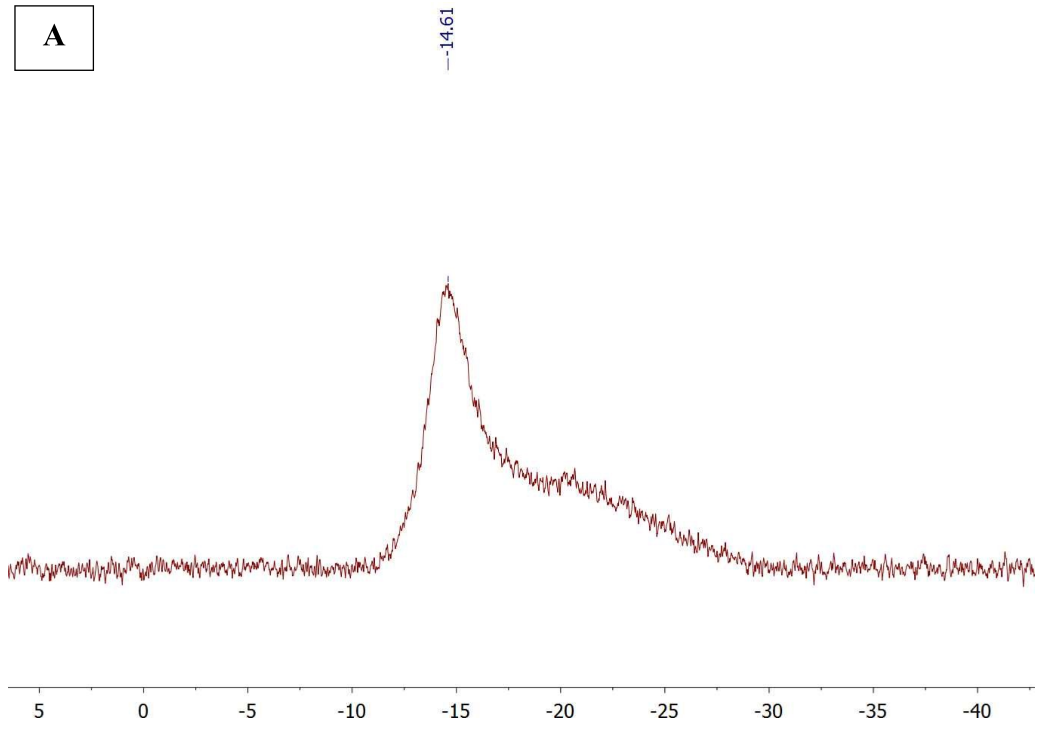

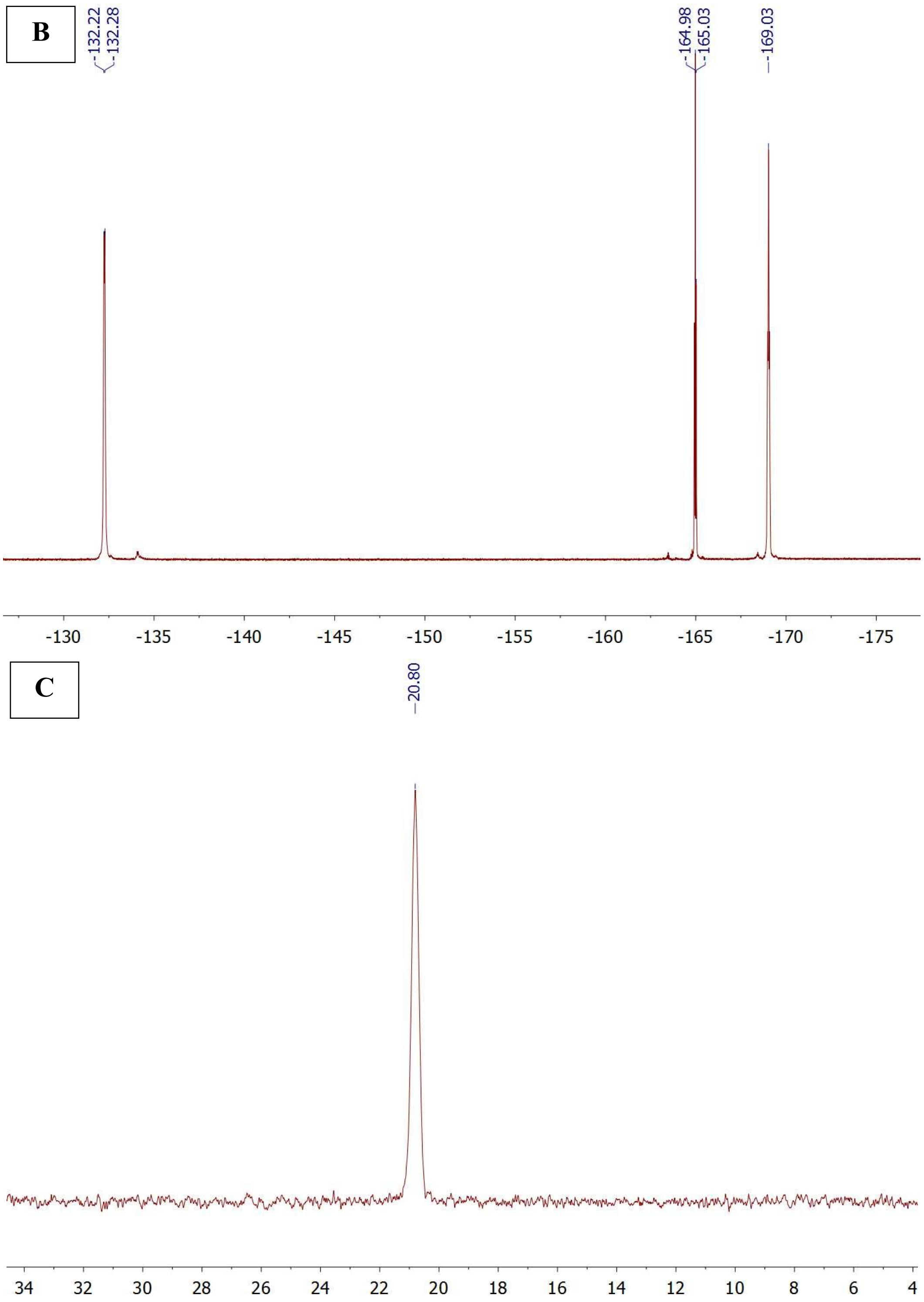

Figure S6. A. ^{11}B NMR, **B**. ^{19}F NMR, and **C**. ^{31}P NMR of complex **3** in MeCN-d3.

2. Electrochemical Measurements

All electrochemical characterization was performed in an inert, nitrogen-filled glove box. Electrochemical characterization was performed with a Gamry Reference 600 potentiostat in a standard three-electrode cell containing a 3 mm diameter glassy carbon (CH Instruments) working electrode, a 0.01 M $Ag^{+/0}$ in 0.1 M $TBAPF_6$/MeCN quasireference electrode (CH Instruments), and a platinum wire counter electrode (Kurt J. Lesker). All voltammograms were compensated for 85% of the measured uncompensated resistance (R_u) value.

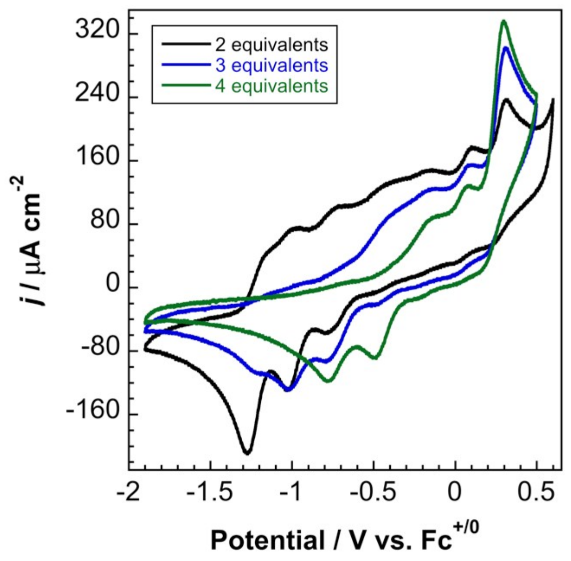

Figure S7. Voltammetry of **1a** in dichloromethane with 0.2 M $TBAPF_6$ with varying stoichiometric equivalents of BPh_3 added to solution.

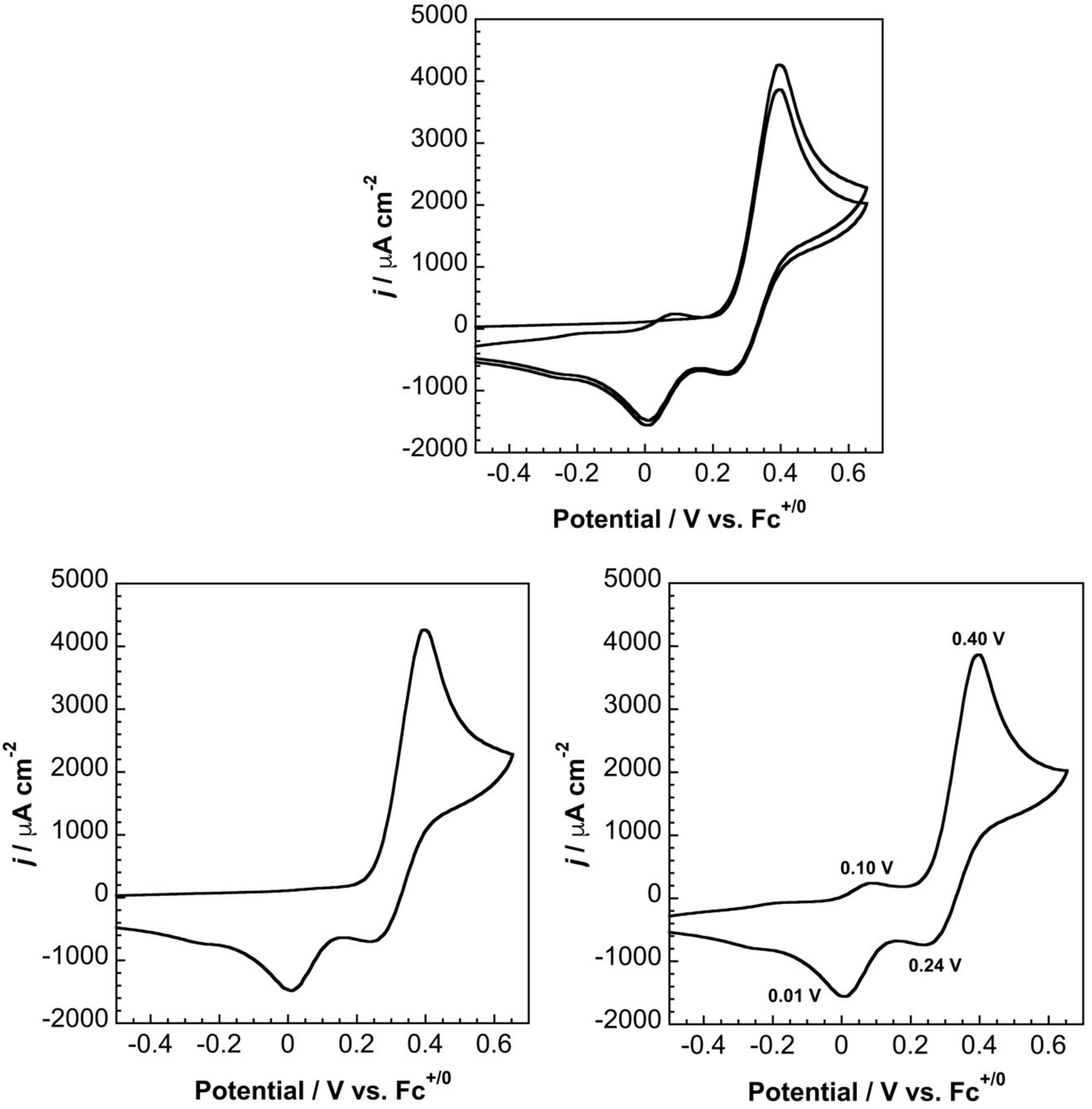

Figure S8. Voltammetry of **2** in acetonitrile with 0.2 M $TBAPF_6$ at 5 V s^{-1}. **Bottom Left**: First scan at 5 V s^{-1}, showing three cathodic peaks. **Bottom Right**: Second scan at 5 V s^{-1} showing re-oxidation of the electrochemically generated four- and five-coordinate species. The anodic potentials match the peak potentials observed in the voltammetry of $(TEA)_4[Fe(CN)_6]$ with different equivalents of BPh_3.

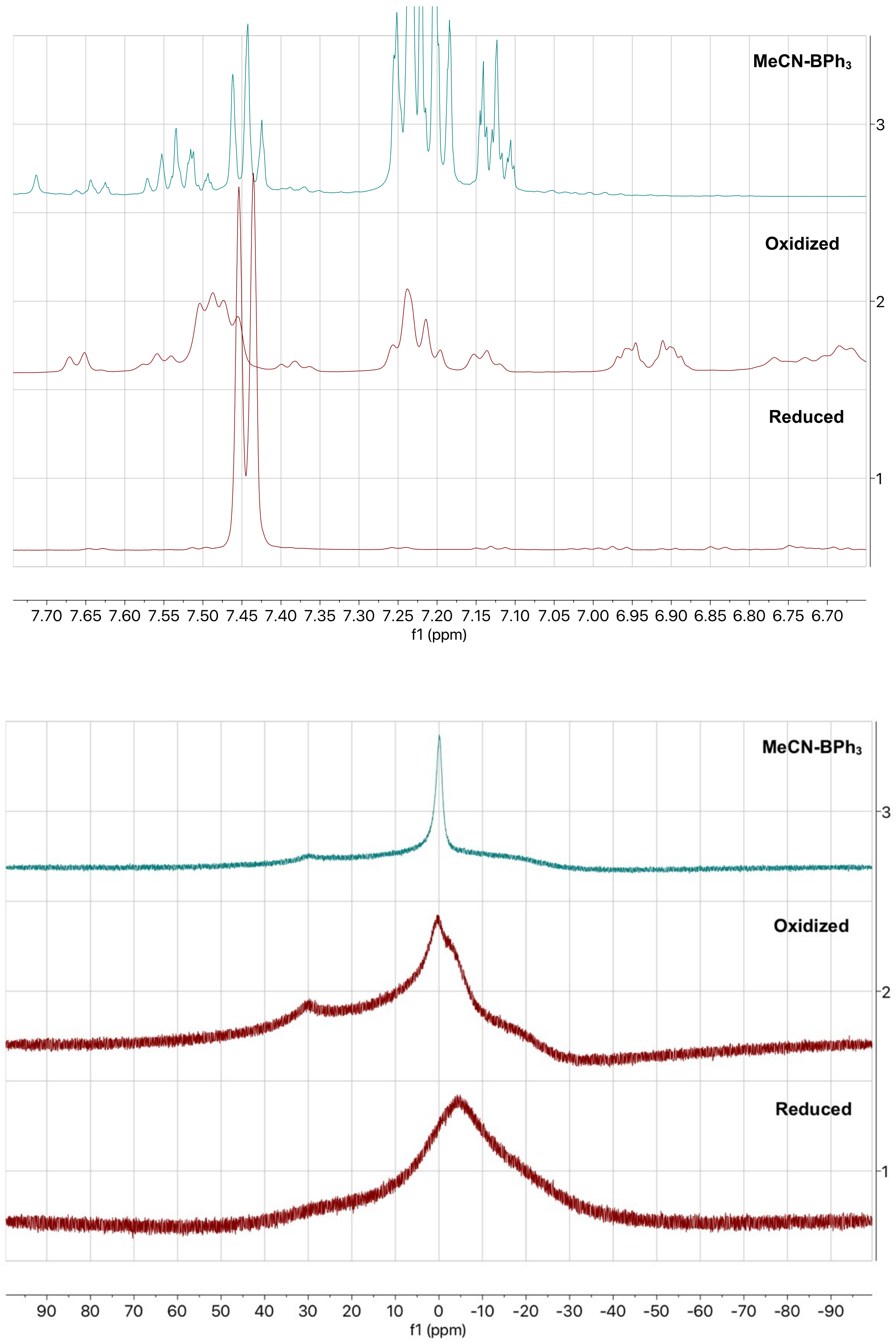

Figure S9. ^{1}H (top) and ^{11}B (bottom) NMR in CD_3CN of free MeCN-BPh_3, oxidized **2**, and **2**, showing the liberation of BPh_3 upon oxidation of **2**. ^{1}H peaks upfield of 7.00 ppm in the oxidized NMR are due to benzo-1,4-dioxin. Additional ^{11}B peaks are due to background signal from borosilicate glass.

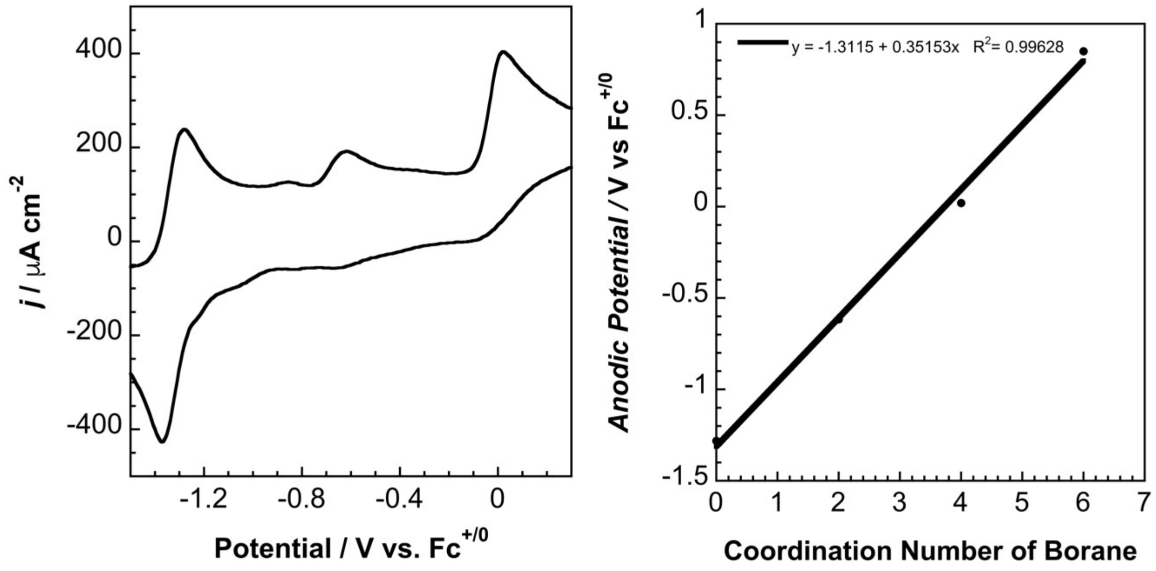

Figure S10. Voltammetry of **1c** in MeCN with 0.2 M $TBAPF_6$ and one equivalent of $B(C_6F_5)_3$.

Table S1. Observed peak anodic potentials from cyclic voltammetry for **1a** in DCM with 0.2 M $TBAPF_6$ with varying stoichiometric equivalents of triphenylborane added to solution.

**Redox Process**	**0 Eq.**	**0.5 Eq.**	**1 Eq.**	**2 Eq.**	**3 Eq.**	**4 Eq.**	**5 Eq.**	**6 Eq.**
**1**	-1.16 V	-1.16 V	-1.14 V	-1.14 V	-1.09 V	---	---	---
**2**	---	---	-0.92 V	-0.92 V	-0.91 V	---	---	---
**3**	---	---	-0.67 V	-0.67 V	-0.64 V	-0.63 V	---	---
**4**	---	---	-0.39 V	-0.39 V	-0.37 V	-0.37 V	---	---
**5**	---	---	---	-0.14 V	-0.14 V	-0.11 V	-0.10 V	-0.10 V
**6**	---	---	---	0.15 V	0.13 V	0.12 V	---	0.11 V
**7**	---	---	---	0.36 V	0.36 V	0.34 V	0.34 V	0.35 V

3. Crystallographic Data

a) Complex 1:

Collection and Refinement of Complex 1b:

Low-temperature diffraction data (φ-and ω-scans) were collected on a Bruker AXS D8 VENTURE KAPPA diffractometer coupled to a PHOTON 100 CMOS detector with Mo K_α radiation (λ = 0.71073 Å) from an IµS micro-source for the structure of compound **1b**. The structure was solved by direct methods using SHELXS[4] and refined against F^2 on all data by full-matrix least squares with SHELXL-2016[5] using established refinement techniques.[6] All non-hydrogen atoms were refined anisotropically. Unless otherwise noted, all hydrogen atoms were included into the model at geometrically calculated positions and refined using a riding model. The isotropic displacement parameters of all hydrogen atoms were fixed to 1.2 times the U value of the atoms they are linked to (1.5 times for methyl groups). All disordered atoms were refined with the help of similarity restraints on the 1,2- and 1,3-distances and displacement parameters as well as enhanced rigid bond restraints for anisotropic displacement parameters.

Compound **1b** crystallizes in the monoclinic space group Cc with one molecule in the asymmetric unit along with four tetraethyl ammonium cations, two molecules of acetonitrile, and five molecules of water. One of the tetraethyl ammonium cations and one of the acetonitrile molecules were disordered over two positions. The coordinates for the hydrogen atoms bound to O1S, O2S, O3S, O4S and O5S were located in the difference Fourier synthesis and refined semi-freely with the help of restraints on the O-H distance (0.84(4) Å) and H-H distance (1.5(4) Å). In addition, the oxygen atom, hydrogen atoms, and hydrogen acceptors were restrained to be flat for all water molecules.

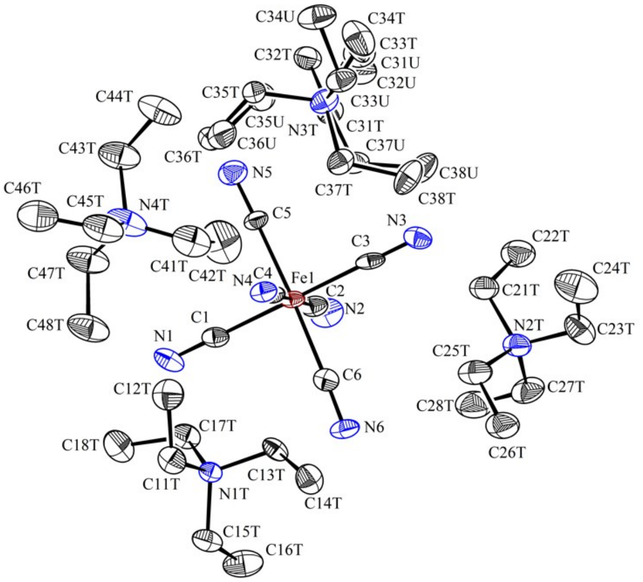

Figure S11. Crystal structure of **1**. Thermal ellipsoids set at 50% probability. Solvent molecules omitted for clarity.

Table S2. Crystal data and structure refinement for complex **1b**.

Empirical formula	C44 H99 Fe N13 O5	
Formula weight	946.21	
Temperature	100(2) K	
Wavelength	0.71073 Å	
Crystal system	Monoclinic	
Space group	Cc	
Unit cell dimensions	a = 16.3801(6) Å	a= 90°.
	b = 15.2273(6) Å	b= 92.0316(14)°.
	c = 22.2072(9) Å	g = 90°.
Volume	5535.5(4) $Å^3$	
Z	4	
Density (calculated)	1.135 Mg/m^3	
Absorption coefficient	0.323 mm^{-1}	
F(000)	2080	
Crystal size	0.300 x 0.300 x 0.250 mm^3	
Theta range for data collection	2.488 to 30.581°.	
Index ranges	-23<=h<=21, -20<=k<=21, -31<=l<=31	
Reflections collected	36004	
Independent reflections	15251 [R(int) = 0.0369]	
Completeness to theta = 25.242°	99.9 %	
Absorption correction	Semi-empirical from equivalents	
Max. and min. transmission	0.7461 and 0.6757	
Refinement method	Full-matrix least-squares on F^2	
Data / restraints / parameters	15251 / 717 / 700	
Goodness-of-fit on F^2	1.041	
Final R indices [I>2sigma(I)]	R1 = 0.0621, wR2 = 0.1517	
R indices (all data)	R1 = 0.0767, wR2 = 0.1605	
Absolute structure parameter	0.050(18)	
Extinction coefficient	n/a	
Largest diff. peak and hole	0.792 and -0.537 e.$Å^{-3}$	

Table S3. Bond lengths [Å] and angles [°] for complex **1b**.

Fe(1)-C(6)	1.918(4)	C(21T)-H(21B)	0.9900
Fe(1)-C(5)	1.919(4)	C(22T)-H(22A)	0.9800
Fe(1)-C(2)	1.920(4)	C(22T)-H(22B)	0.9800
Fe(1)-C(3)	1.922(5)	C(22T)-H(22C)	0.9800
Fe(1)-C(4)	1.924(4)	C(23T)-C(24T)	1.544(9)
Fe(1)-C(1)	1.926(5)	C(23T)-H(23A)	0.9900
C(1)-N(1)	1.174(6)	C(23T)-H(23B)	0.9900
C(2)-N(2)	1.164(6)	C(24T)-H(24A)	0.9800
C(3)-N(3)	1.168(6)	C(24T)-H(24B)	0.9800
C(4)-N(4)	1.170(6)	C(24T)-H(24C)	0.9800
C(5)-N(5)	1.165(6)	C(25T)-C(26T)	1.492(8)
C(6)-N(6)	1.156(6)	C(25T)-H(25A)	0.9900
N(1T)-C(13T)	1.509(5)	C(25T)-H(25B)	0.9900
N(1T)-C(15T)	1.512(5)	C(26T)-H(26A)	0.9800
N(1T)-C(11T)	1.517(5)	C(26T)-H(26B)	0.9800
N(1T)-C(17T)	1.518(5)	C(26T)-H(26C)	0.9800
C(11T)-C(12T)	1.492(7)	C(27T)-C(28T)	1.463(9)
C(11T)-H(11A)	0.9900	C(27T)-H(27A)	0.9900
C(11T)-H(11B)	0.9900	C(27T)-H(27B)	0.9900
C(12T)-H(12A)	0.9800	C(28T)-H(28A)	0.9800
C(12T)-H(12B)	0.9800	C(28T)-H(28B)	0.9800
C(12T)-H(12C)	0.9800	C(28T)-H(28C)	0.9800
C(13T)-C(14T)	1.522(6)	N(3T)-C(35U)	1.440(11)
C(13T)-H(13A)	0.9900	N(3T)-C(33T)	1.458(7)
C(13T)-H(13B)	0.9900	N(3T)-C(31T)	1.499(7)
C(14T)-H(14A)	0.9800	N(3T)-C(37U)	1.513(9)
C(14T)-H(14B)	0.9800	N(3T)-C(33U)	1.531(9)
C(14T)-H(14C)	0.9800	N(3T)-C(37T)	1.540(8)
C(15T)-C(16T)	1.514(7)	N(3T)-C(35T)	1.591(8)
C(15T)-H(15A)	0.9900	N(3T)-C(31U)	1.605(10)
C(15T)-H(15B)	0.9900	C(31T)-C(32T)	1.520(10)
C(16T)-H(16A)	0.9800	C(31T)-H(31A)	0.9900
C(16T)-H(16B)	0.9800	C(31T)-H(31B)	0.9900
C(16T)-H(16C)	0.9800	C(32T)-H(32A)	0.9800
C(17T)-C(18T)	1.502(7)	C(32T)-H(32B)	0.9800
C(17T)-H(17A)	0.9900	C(32T)-H(32C)	0.9800
C(17T)-H(17B)	0.9900	C(33T)-C(34T)	1.527(10)
C(18T)-H(18A)	0.9800	C(33T)-H(33A)	0.9900
C(18T)-H(18B)	0.9800	C(33T)-H(33B)	0.9900
C(18T)-H(18C)	0.9800	C(34T)-H(34A)	0.9800
N(2T)-C(27T)	1.505(6)	C(34T)-H(34B)	0.9800
N(2T)-C(21T)	1.519(6)	C(34T)-H(34C)	0.9800
N(2T)-C(25T)	1.527(6)	C(35T)-C(36T)	1.516(10)
N(2T)-C(23T)	1.533(7)	C(35T)-H(35A)	0.9900
C(21T)-C(22T)	1.517(7)	C(35T)-H(35B)	0.9900
C(21T)-H(21A)	0.9900	C(36T)-H(36A)	0.9800

C(36T)-H(36B)	0.9800	C(44T)-H(44B)	0.9800
C(36T)-H(36C)	0.9800	C(44T)-H(44C)	0.9800
C(37T)-C(38T)	1.520(10)	C(45T)-C(46T)	1.457(9)
C(37T)-H(37A)	0.9900	C(45T)-H(45A)	0.9900
C(37T)-H(37B)	0.9900	C(45T)-H(45B)	0.9900
C(38T)-H(38A)	0.9800	C(46T)-H(46A)	0.9800
C(38T)-H(38B)	0.9800	C(46T)-H(46B)	0.9800
C(38T)-H(38C)	0.9800	C(46T)-H(46C)	0.9800
C(31U)-C(32U)	1.512(12)	C(47T)-C(48T)	1.531(7)
C(31U)-H(31C)	0.9900	C(47T)-H(47A)	0.9900
C(31U)-H(31D)	0.9900	C(47T)-H(47B)	0.9900
C(32U)-H(32D)	0.9800	C(48T)-H(48A)	0.9800
C(32U)-H(32E)	0.9800	C(48T)-H(48B)	0.9800
C(32U)-H(32F)	0.9800	C(48T)-H(48C)	0.9800
C(33U)-C(34U)	1.531(12)	N(1S)-C(11S)	1.134(7)
C(33U)-H(33C)	0.9900	C(11S)-C(12S)	1.445(7)
C(33U)-H(33D)	0.9900	C(12S)-H(12D)	0.9800
C(34U)-H(34D)	0.9800	C(12S)-H(12E)	0.9800
C(34U)-H(34E)	0.9800	C(12S)-H(12F)	0.9800
C(34U)-H(34F)	0.9800	N(2S)-C(21S)	1.141(6)
C(35U)-C(36U)	1.512(12)	C(21S)-C(22S)	1.444(6)
C(35U)-H(35C)	0.9900	C(22S)-H(22D)	0.9800
C(35U)-H(35D)	0.9900	C(22S)-H(22E)	0.9800
C(36U)-H(36D)	0.9800	C(22S)-H(22F)	0.9800
C(36U)-H(36E)	0.9800	N(3S)-C(31S)	1.116(8)
C(36U)-H(36F)	0.9800	C(31S)-C(32S)	1.458(9)
C(37U)-C(38U)	1.516(13)	C(32S)-H(32G)	0.9800
C(37U)-H(37C)	0.9900	C(32S)-H(32H)	0.9800
C(37U)-H(37D)	0.9900	C(32S)-H(32I)	0.9800
C(38U)-H(38D)	0.9800	N(4S)-C(41S)	1.12(2)
C(38U)-H(38E)	0.9800	C(41S)-C(42S)	1.50(2)
C(38U)-H(38F)	0.9800	C(42S)-H(42G)	0.9800
N(4T)-C(41T)	1.473(8)	C(42S)-H(42H)	0.9800
N(4T)-C(47T)	1.509(6)	C(42S)-H(42I)	0.9800
N(4T)-C(45T)	1.529(6)	O(1S)-H(1S1)	0.86(3)
N(4T)-C(43T)	1.540(6)	O(1S)-H(1S2)	0.86(3)
C(41T)-C(42T)	1.519(8)	O(2S)-H(2S1)	0.85(3)
C(41T)-H(41A)	0.9900	O(2S)-H(2S2)	0.83(3)
C(41T)-H(41B)	0.9900	O(3S)-H(3S1)	0.86(3)
C(42T)-H(42A)	0.9800	O(3S)-H(3S2)	0.89(3)
C(42T)-H(42B)	0.9800	O(4S)-H(4S1)	0.86(3)
C(42T)-H(42C)	0.9800	O(4S)-H(4S2)	0.85(3)
C(43T)-C(44T)	1.513(8)	O(5S)-H(5S1)	0.87(3)
C(43T)-H(43A)	0.9900	O(5S)-H(5S2)	0.88(3)
C(43T)-H(43B)	0.9900		
C(44T)-H(44A)	0.9800	C(6)-Fe(1)-C(5)	177.67(19)

C(6)-Fe(1)-C(2)	88.27(17)
C(5)-Fe(1)-C(2)	92.74(18)
C(6)-Fe(1)-C(3)	92.17(18)
C(5)-Fe(1)-C(3)	89.95(16)
C(2)-Fe(1)-C(3)	89.28(18)
C(6)-Fe(1)-C(4)	89.68(15)
C(5)-Fe(1)-C(4)	89.35(17)
C(2)-Fe(1)-C(4)	177.7(2)
C(3)-Fe(1)-C(4)	89.78(17)
C(6)-Fe(1)-C(1)	88.47(17)
C(5)-Fe(1)-C(1)	89.46(19)
C(2)-Fe(1)-C(1)	88.10(19)
C(3)-Fe(1)-C(1)	177.29(18)
C(4)-Fe(1)-C(1)	92.86(17)
N(1)-C(1)-Fe(1)	176.4(4)
N(2)-C(2)-Fe(1)	177.2(4)
N(3)-C(3)-Fe(1)	179.1(4)
N(4)-C(4)-Fe(1)	178.6(4)
N(5)-C(5)-Fe(1)	179.0(4)
N(6)-C(6)-Fe(1)	178.0(4)
C(13T)-N(1T)-C(15T)	112.8(3)
C(13T)-N(1T)-C(11T)	111.1(3)
C(15T)-N(1T)-C(11T)	105.2(3)
C(13T)-N(1T)-C(17T)	105.3(3)
C(15T)-N(1T)-C(17T)	112.2(3)
C(11T)-N(1T)-C(17T)	110.5(3)
C(12T)-C(11T)-N(1T)	115.2(4)
C(12T)-C(11T)-H(11A)	108.5
N(1T)-C(11T)-H(11A)	108.5
C(12T)-C(11T)-H(11B)	108.5
N(1T)-C(11T)-H(11B)	108.5
H(11A)-C(11T)-H(11B)	107.5
C(11T)-C(12T)-H(12A)	109.5
C(11T)-C(12T)-H(12B)	109.5
H(12A)-C(12T)-H(12B)	109.5
C(11T)-C(12T)-H(12C)	109.5
H(12A)-C(12T)-H(12C)	109.5
H(12B)-C(12T)-H(12C)	109.5
N(1T)-C(13T)-C(14T)	114.5(3)
N(1T)-C(13T)-H(13A)	108.6
C(14T)-C(13T)-H(13A)	108.6
N(1T)-C(13T)-H(13B)	108.6
C(14T)-C(13T)-H(13B)	108.6
H(13A)-C(13T)-H(13B)	107.6
C(13T)-C(14T)-H(14A)	109.5
C(13T)-C(14T)-H(14B)	109.5
H(14A)-C(14T)-H(14B)	109.5
C(13T)-C(14T)-H(14C)	109.5
H(14A)-C(14T)-H(14C)	109.5
H(14B)-C(14T)-H(14C)	109.5
N(1T)-C(15T)-C(16T)	114.7(4)
N(1T)-C(15T)-H(15A)	108.6
C(16T)-C(15T)-H(15A)	108.6
N(1T)-C(15T)-H(15B)	108.6
C(16T)-C(15T)-H(15B)	108.6
H(15A)-C(15T)-H(15B)	107.6
C(15T)-C(16T)-H(16A)	109.5
C(15T)-C(16T)-H(16B)	109.5
H(16A)-C(16T)-H(16B)	109.5
C(15T)-C(16T)-H(16C)	109.5
H(16A)-C(16T)-H(16C)	109.5
H(16B)-C(16T)-H(16C)	109.5
C(18T)-C(17T)-N(1T)	114.6(3)
C(18T)-C(17T)-H(17A)	108.6
N(1T)-C(17T)-H(17A)	108.6
C(18T)-C(17T)-H(17B)	108.6
N(1T)-C(17T)-H(17B)	108.6
H(17A)-C(17T)-H(17B)	107.6
C(17T)-C(18T)-H(18A)	109.5
C(17T)-C(18T)-H(18B)	109.5
H(18A)-C(18T)-H(18B)	109.5
C(17T)-C(18T)-H(18C)	109.5
H(18A)-C(18T)-H(18C)	109.5
H(18B)-C(18T)-H(18C)	109.5
C(27T)-N(2T)-C(21T)	111.9(4)
C(27T)-N(2T)-C(25T)	111.0(4)
C(21T)-N(2T)-C(25T)	105.1(3)
C(27T)-N(2T)-C(23T)	107.1(4)
C(21T)-N(2T)-C(23T)	111.1(4)
C(25T)-N(2T)-C(23T)	110.7(4)
C(22T)-C(21T)-N(2T)	113.9(4)
C(22T)-C(21T)-H(21A)	108.8
N(2T)-C(21T)-H(21A)	108.8
C(22T)-C(21T)-H(21B)	108.8
N(2T)-C(21T)-H(21B)	108.8
H(21A)-C(21T)-H(21B)	107.7
C(21T)-C(22T)-H(22A)	109.5
C(21T)-C(22T)-H(22B)	109.5
H(22A)-C(22T)-H(22B)	109.5
C(21T)-C(22T)-H(22C)	109.5
H(22A)-C(22T)-H(22C)	109.5
H(22B)-C(22T)-H(22C)	109.5

N(2T)-C(23T)-C(24T)	113.4(5)
N(2T)-C(23T)-H(23A)	108.9
C(24T)-C(23T)-H(23A)	108.9
N(2T)-C(23T)-H(23B)	108.9
C(24T)-C(23T)-H(23B)	108.9
H(23A)-C(23T)-H(23B)	107.7
C(23T)-C(24T)-H(24A)	109.5
C(23T)-C(24T)-H(24B)	109.5
H(24A)-C(24T)-H(24B)	109.5
C(23T)-C(24T)-H(24C)	109.5
H(24A)-C(24T)-H(24C)	109.5
H(24B)-C(24T)-H(24C)	109.5
C(26T)-C(25T)-N(2T)	113.9(4)
C(26T)-C(25T)-H(25A)	108.8
N(2T)-C(25T)-H(25A)	108.8
C(26T)-C(25T)-H(25B)	108.8
N(2T)-C(25T)-H(25B)	108.8
H(25A)-C(25T)-H(25B)	107.7
C(25T)-C(26T)-H(26A)	109.5
C(25T)-C(26T)-H(26B)	109.5
H(26A)-C(26T)-H(26B)	109.5
C(25T)-C(26T)-H(26C)	109.5
H(26A)-C(26T)-H(26C)	109.5
H(26B)-C(26T)-H(26C)	109.5
C(28T)-C(27T)-N(2T)	115.3(4)
C(28T)-C(27T)-H(27A)	108.4
N(2T)-C(27T)-H(27A)	108.4
C(28T)-C(27T)-H(27B)	108.4
N(2T)-C(27T)-H(27B)	108.4
H(27A)-C(27T)-H(27B)	107.5
C(27T)-C(28T)-H(28A)	109.5
C(27T)-C(28T)-H(28B)	109.5
H(28A)-C(28T)-H(28B)	109.5
C(27T)-C(28T)-H(28C)	109.5
H(28A)-C(28T)-H(28C)	109.5
H(28B)-C(28T)-H(28C)	109.5
C(33T)-N(3T)-C(31T)	113.6(5)
C(35U)-N(3T)-C(37U)	115.9(7)
C(35U)-N(3T)-C(33U)	116.5(7)
C(37U)-N(3T)-C(33U)	108.0(6)
C(33T)-N(3T)-C(37T)	114.4(5)
C(31T)-N(3T)-C(37T)	108.0(4)
C(33T)-N(3T)-C(35T)	108.3(4)
C(31T)-N(3T)-C(35T)	108.8(5)
C(37T)-N(3T)-C(35T)	103.1(5)
C(35U)-N(3T)-C(31U)	105.6(6)
C(37U)-N(3T)-C(31U)	106.2(6)
C(33U)-N(3T)-C(31U)	103.3(6)
N(3T)-C(31T)-C(32T)	114.8(6)
N(3T)-C(31T)-H(31A)	108.6
C(32T)-C(31T)-H(31A)	108.6
N(3T)-C(31T)-H(31B)	108.6
C(32T)-C(31T)-H(31B)	108.6
H(31A)-C(31T)-H(31B)	107.5
C(31T)-C(32T)-H(32A)	109.5
C(31T)-C(32T)-H(32B)	109.5
H(32A)-C(32T)-H(32B)	109.5
C(31T)-C(32T)-H(32C)	109.5
H(32A)-C(32T)-H(32C)	109.5
H(32B)-C(32T)-H(32C)	109.5
N(3T)-C(33T)-C(34T)	116.3(7)
N(3T)-C(33T)-H(33A)	108.2
C(34T)-C(33T)-H(33A)	108.2
N(3T)-C(33T)-H(33B)	108.2
C(34T)-C(33T)-H(33B)	108.2
H(33A)-C(33T)-H(33B)	107.4
C(33T)-C(34T)-H(34A)	109.5
C(33T)-C(34T)-H(34B)	109.5
H(34A)-C(34T)-H(34B)	109.5
C(33T)-C(34T)-H(34C)	109.5
H(34A)-C(34T)-H(34C)	109.5
H(34B)-C(34T)-H(34C)	109.5
C(36T)-C(35T)-N(3T)	116.0(6)
C(36T)-C(35T)-H(35A)	108.3
N(3T)-C(35T)-H(35A)	108.3
C(36T)-C(35T)-H(35B)	108.3
N(3T)-C(35T)-H(35B)	108.3
H(35A)-C(35T)-H(35B)	107.4
C(35T)-C(36T)-H(36A)	109.5
C(35T)-C(36T)-H(36B)	109.5
H(36A)-C(36T)-H(36B)	109.5
C(35T)-C(36T)-H(36C)	109.5
H(36A)-C(36T)-H(36C)	109.5
H(36B)-C(36T)-H(36C)	109.5
C(38T)-C(37T)-N(3T)	111.6(7)
C(38T)-C(37T)-H(37A)	109.3
N(3T)-C(37T)-H(37A)	109.3
C(38T)-C(37T)-H(37B)	109.3
N(3T)-C(37T)-H(37B)	109.3
H(37A)-C(37T)-H(37B)	108.0
C(37T)-C(38T)-H(38A)	109.5
C(37T)-C(38T)-H(38B)	109.5

H(38A)-C(38T)-H(38B)	109.5
C(37T)-C(38T)-H(38C)	109.5
H(38A)-C(38T)-H(38C)	109.5
H(38B)-C(38T)-H(38C)	109.5
C(32U)-C(31U)-N(3T)	114.0(8)
C(32U)-C(31U)-H(31C)	108.8
N(3T)-C(31U)-H(31C)	108.8
C(32U)-C(31U)-H(31D)	108.8
N(3T)-C(31U)-H(31D)	108.8
H(31C)-C(31U)-H(31D)	107.6
C(31U)-C(32U)-H(32D)	109.5
C(31U)-C(32U)-H(32E)	109.5
H(32D)-C(32U)-H(32E)	109.5
C(31U)-C(32U)-H(32F)	109.5
H(32D)-C(32U)-H(32F)	109.5
H(32E)-C(32U)-H(32F)	109.5
N(3T)-C(33U)-C(34U)	116.1(9)
N(3T)-C(33U)-H(33C)	108.3
C(34U)-C(33U)-H(33C)	108.3
N(3T)-C(33U)-H(33D)	108.3
C(34U)-C(33U)-H(33D)	108.3
H(33C)-C(33U)-H(33D)	107.4
C(33U)-C(34U)-H(34D)	109.5
C(33U)-C(34U)-H(34E)	109.5
H(34D)-C(34U)-H(34E)	109.5
C(33U)-C(34U)-H(34F)	109.5
H(34D)-C(34U)-H(34F)	109.5
H(34E)-C(34U)-H(34F)	109.5
N(3T)-C(35U)-C(36U)	110.4(10)
N(3T)-C(35U)-H(35C)	109.6
C(36U)-C(35U)-H(35C)	109.6
N(3T)-C(35U)-H(35D)	109.6
C(36U)-C(35U)-H(35D)	109.6
H(35C)-C(35U)-H(35D)	108.1
C(35U)-C(36U)-H(36D)	109.5
C(35U)-C(36U)-H(36E)	109.5
H(36D)-C(36U)-H(36E)	109.5
C(35U)-C(36U)-H(36F)	109.5
H(36D)-C(36U)-H(36F)	109.5
H(36E)-C(36U)-H(36F)	109.5
N(3T)-C(37U)-C(38U)	118.3(8)
N(3T)-C(37U)-H(37C)	107.7
C(38U)-C(37U)-H(37C)	107.7
N(3T)-C(37U)-H(37D)	107.7
C(38U)-C(37U)-H(37D)	107.7
H(37C)-C(37U)-H(37D)	107.1
C(37U)-C(38U)-H(38D)	109.5
C(37U)-C(38U)-H(38E)	109.5
H(38D)-C(38U)-H(38E)	109.5
C(37U)-C(38U)-H(38F)	109.5
H(38D)-C(38U)-H(38F)	109.5
H(38E)-C(38U)-H(38F)	109.5
C(41T)-N(4T)-C(47T)	113.6(5)
C(41T)-N(4T)-C(45T)	106.5(4)
C(47T)-N(4T)-C(45T)	110.8(4)
C(41T)-N(4T)-C(43T)	111.6(5)
C(47T)-N(4T)-C(43T)	104.9(4)
C(45T)-N(4T)-C(43T)	109.5(4)
N(4T)-C(41T)-C(42T)	113.4(5)
N(4T)-C(41T)-H(41A)	108.9
C(42T)-C(41T)-H(41A)	108.9
N(4T)-C(41T)-H(41B)	108.9
C(42T)-C(41T)-H(41B)	108.9
H(41A)-C(41T)-H(41B)	107.7
C(41T)-C(42T)-H(42A)	109.5
C(41T)-C(42T)-H(42B)	109.5
H(42A)-C(42T)-H(42B)	109.5
C(41T)-C(42T)-H(42C)	109.5
H(42A)-C(42T)-H(42C)	109.5
H(42B)-C(42T)-H(42C)	109.5
C(44T)-C(43T)-N(4T)	114.4(5)
C(44T)-C(43T)-H(43A)	108.7
N(4T)-C(43T)-H(43A)	108.7
C(44T)-C(43T)-H(43B)	108.7
N(4T)-C(43T)-H(43B)	108.7
H(43A)-C(43T)-H(43B)	107.6
C(43T)-C(44T)-H(44A)	109.5
C(43T)-C(44T)-H(44B)	109.5
H(44A)-C(44T)-H(44B)	109.5
C(43T)-C(44T)-H(44C)	109.5
H(44A)-C(44T)-H(44C)	109.5
H(44B)-C(44T)-H(44C)	109.5
C(46T)-C(45T)-N(4T)	114.7(5)
C(46T)-C(45T)-H(45A)	108.6
N(4T)-C(45T)-H(45A)	108.6
C(46T)-C(45T)-H(45B)	108.6
N(4T)-C(45T)-H(45B)	108.6
H(45A)-C(45T)-H(45B)	107.6
C(45T)-C(46T)-H(46A)	109.5
C(45T)-C(46T)-H(46B)	109.5
H(46A)-C(46T)-H(46B)	109.5
C(45T)-C(46T)-H(46C)	109.5

H(46A)-C(46T)-H(46C)	109.5
H(46B)-C(46T)-H(46C)	109.5
N(4T)-C(47T)-C(48T)	111.9(5)
N(4T)-C(47T)-H(47A)	109.2
C(48T)-C(47T)-H(47A)	109.2
N(4T)-C(47T)-H(47B)	109.2
C(48T)-C(47T)-H(47B)	109.2
H(47A)-C(47T)-H(47B)	107.9
C(47T)-C(48T)-H(48A)	109.5
C(47T)-C(48T)-H(48B)	109.5
H(48A)-C(48T)-H(48B)	109.5
C(47T)-C(48T)-H(48C)	109.5
H(48A)-C(48T)-H(48C)	109.5
H(48B)-C(48T)-H(48C)	109.5
N(1S)-C(11S)-C(12S)	178.2(5)
C(11S)-C(12S)-H(12D)	109.5
C(11S)-C(12S)-H(12E)	109.5
H(12D)-C(12S)-H(12E)	109.5
C(11S)-C(12S)-H(12F)	109.5
H(12D)-C(12S)-H(12F)	109.5
H(12E)-C(12S)-H(12F)	109.5
N(2S)-C(21S)-C(22S)	178.0(6)
C(21S)-C(22S)-H(22D)	109.5
C(21S)-C(22S)-H(22E)	109.5
H(22D)-C(22S)-H(22E)	109.5
C(21S)-C(22S)-H(22F)	109.5
H(22D)-C(22S)-H(22F)	109.5
H(22E)-C(22S)-H(22F)	109.5
N(3S)-C(31S)-C(32S)	175.8(7)
C(31S)-C(32S)-H(32G)	109.5
C(31S)-C(32S)-H(32H)	109.5
H(32G)-C(32S)-H(32H)	109.5
C(31S)-C(32S)-H(32I)	109.5
H(32G)-C(32S)-H(32I)	109.5
H(32H)-C(32S)-H(32I)	109.5
N(4S)-C(41S)-C(42S)	168(3)
C(41S)-C(42S)-H(42G)	109.5
C(41S)-C(42S)-H(42H)	109.5
H(42G)-C(42S)-H(42H)	109.5
C(41S)-C(42S)-H(42I)	109.5
H(42G)-C(42S)-H(42I)	109.5
H(42H)-C(42S)-H(42I)	109.5
H(1S1)-O(1S)-H(1S2)	118(5)
H(2S1)-O(2S)-H(2S2)	126(5)
H(3S1)-O(3S)-H(3S2)	120(5)
H(4S1)-O(4S)-H(4S2)	117(5)
H(5S1)-O(5S)-H(5S2)	114(5)

b) Complex 2:

<u>Collection and Refinement of Complex 2:</u>

Low-temperature diffraction data (ϕ-and ω-scans) were collected on Bruker AXS D8 VENTURE KAPPA diffractometer coupled to a PHOTON 100 CMOS detector with Cu K_α radiation (λ = 1.54178 Å) from an IμS micro-source for the structure of $(TEA)_4[Fe(CN\text{-}B(C_6H_5)_3)_6]$ (**2**). The structure was solved by direct methods using SHELXS[4] and refined against F^2 on all data by full-matrix least squares with SHELXL-2014[5] using established refinement techniques.[6] All non-hydrogen atoms were refined anisotropically. All hydrogen atoms were included into the model at geometrically calculated positions and refined using a riding model. The isotropic displacement parameters of all hydrogen atoms were fixed to 1.2 times the U value of the atoms they are linked to (1.5 times for methyl groups). All disordered atoms were refined with the help of similarity restraints on the 1,2- and 1,3-distances and displacement parameters as well as rigid bond restraints for anisotropic displacement parameters.

$(TEA)_4[Fe(CN\text{-}B(C_6H_5)_3)_6]$ (**2**) crystallizes in the orthorhombic space group $P2_12_12_1$ with one molecule in the asymmetric unit along with four molecules of tetraethylammonium and 2.58 molecules of dichloromethane. Two of the triphenyl borane groups and tetraethylammonium cations were disordered over two positions. One of the dichloromethane molecules was disordered over two positions. The remaining 1.58 molecules of dichloromethane were disordered over four positions.

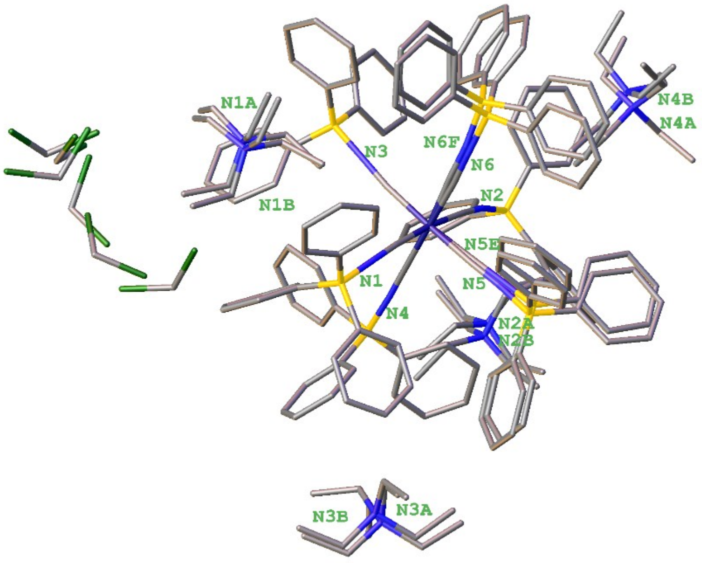

N1A
N1B
N3
N6F
N6
N2
N5E
N1
N5
N4
N2A
N2B
N4B
N4A
N3B
N3A

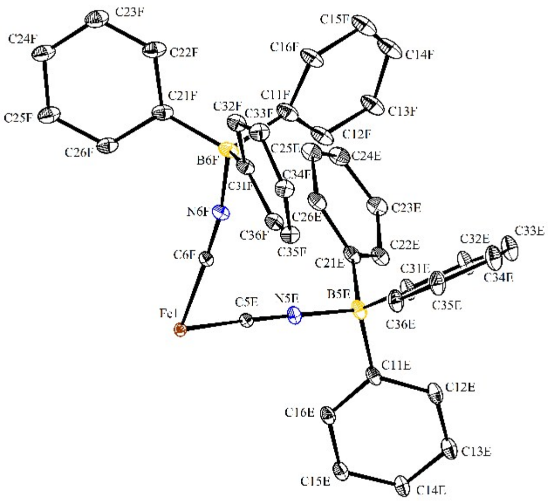

C23F
C24F
C22F
C15F
C16F
C14F
C21F
C11F
C32F
C33F
C13F
C25F
C12F
C25E
C24E
C26F
B6F
C34F
C31F
C23E
N6F
C26E
C36F
C35F
C22E
C6F
C21E
C32E
C33E
C31E
C34E
C5E
N5E
B5E
C35E
Fe1
C36E
C11E
C12E
C16E
C13E
C15E
C14E

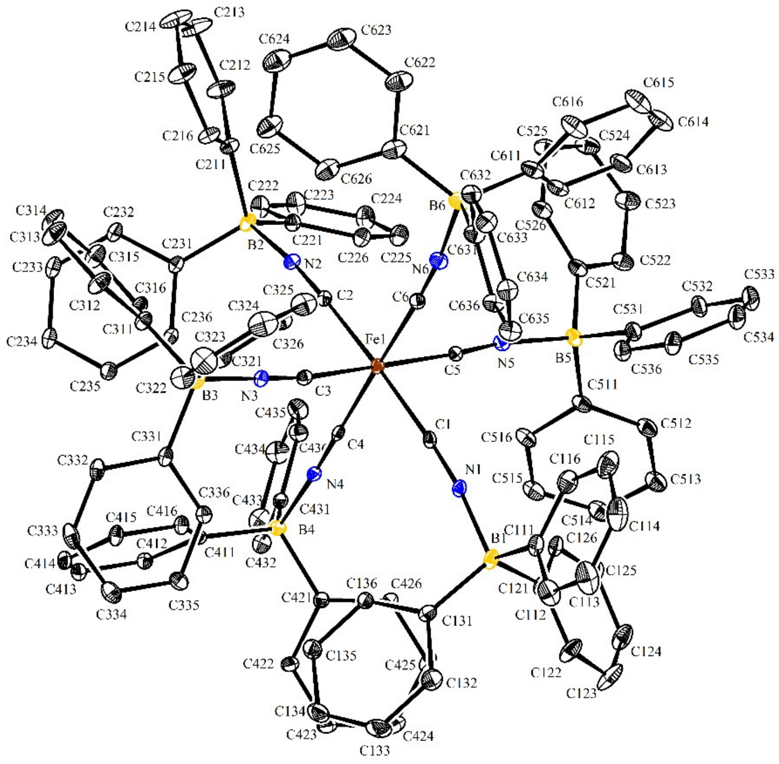

C213
C214
C623
C624
C212
C622
C215
C615
C616
C614
C621
C216
C525
C524
C625
C611
C613
C211
C626
C632
C222
C223
C224
B6
C612
C523
C314
C232
C526
C313
C315
C231
B2
C221
C633
C226
C225
N2
C631
C522
C233
N6
C634
C312
C316
C324
C325
C2
C533
C532
C236
C311
C6
C636
C531
C323
C635
C234
C321
C326
Fe1
N5
B5
C534
C322
B3
N3
C3
C5
C536
C535
C235
C435
C1
C511
C516
C512
C331
C434
C4
C436
C115
C332
N4
N1
C116
C336
C513
C433
C431
C515
C415
C416
C114
C333
C412
C411
B4
C514
B1
C111
C126
C414
C432
C125
C413
C121
C421
C136
C426
C131
C112
C113
C334
C335
C124
C422
C135
C425
C132
C122
C134
C123
C423
C133
C424

Figure S12. Crystal structure of **2** showing the disorder of two triphenylborane moieties. Thermal ellipsoids set at 50% probability.

Table S4. Crystal data and structure refinement for complex **2.**

Empirical formula	C148.57 H175.16 B6 Cl5.16 Fe N10	
Formula weight	2404.67	
Temperature	100(2) K	
Wavelength	1.54178 Å	
Crystal system	Orthorhombic	
Space group	$P2_12_12_1$	
Unit cell dimensions	a = 17.5522(6) Å	a= 90°.
	b = 21.2914(7) Å	b= 90°.
	c = 35.9929(11) Å	g = 90°.
Volume	13450.9(8) Å3	
Z	4	
Density (calculated)	1.187 Mg/m3	
Absorption coefficient	2.250 mm-1	
F(000)	5121	
Crystal size	0.200 x 0.150 x 0.050 mm3	
Theta range for data collection	2.411 to 74.493°.	
Index ranges	-21<=h<=21, -26<=k<=26, -44<=l<=44	
Reflections collected	155470	
Independent reflections	27477 [R(int) = 0.1619]	
Completeness to theta =	67.679°	100.0 %
Absorption correction	Semi-empirical from equivalents	
Max. and min. transmission	0.5953 and 0.4527	
Refinement method	Full-matrix least-squares on F2	
Data / restraints / parameters	27477 / 8929 / 2341	
Goodness-of-fit on F2	1.024	
Final R indices [I>2sigma(I)]	R1 = 0.1006, wR2 = 0.2528	

R indices (all data) R1 = 0.1439, wR2 = 0.2890

Absolute structure parameter 0.392(3)

Extinction coefficient n/a

Largest diff. peak and hole 0.726 and -0.575 e.Å-3

Table S5. Bond lengths [Å] and angles [°] for complex **2.**

Fe(1)-C(6)	1.862(13)	C(115)-C(116)	1.400(13)
Fe(1)-C(5E)	1.885(15)	C(115)-H(115)	0.9500
Fe(1)-C(4)	1.908(8)	C(116)-H(116)	0.9500
Fe(1)-C(1)	1.908(7)	C(121)-C(126)	1.388(10)
Fe(1)-C(5)	1.909(14)	C(121)-C(122)	1.391(11)
Fe(1)-C(3)	1.913(8)	C(122)-C(123)	1.397(13)
Fe(1)-C(6F)	1.917(16)	C(122)-H(122)	0.9500
Fe(1)-C(2)	1.925(7)	C(123)-C(124)	1.374(13)
C(1)-N(1)	1.170(9)	C(123)-H(123)	0.9500
N(1)-B(1)	1.559(9)	C(124)-C(125)	1.371(12)
B(1)-C(131)	1.622(11)	C(124)-H(124)	0.9500
B(1)-C(111)	1.635(11)	C(125)-C(126)	1.393(11)
B(1)-C(121)	1.637(11)	C(125)-H(125)	0.9500
C(111)-C(112)	1.364(11)	C(126)-H(126)	0.9500
C(111)-C(116)	1.386(12)	C(131)-C(136)	1.393(11)
C(112)-C(113)	1.388(13)	C(131)-C(132)	1.398(11)
C(112)-H(112)	0.9500	C(132)-C(133)	1.405(13)
C(113)-C(114)	1.384(15)	C(132)-H(132)	0.9500
C(113)-H(113)	0.9500	C(133)-C(134)	1.357(13)
C(114)-C(115)	1.383(15)	C(133)-H(133)	0.9500
C(114)-H(114)	0.9500	C(134)-C(135)	1.368(12)

C(134)-H(134)	0.9500
C(135)-C(136)	1.400(11)
C(135)-H(135)	0.9500
C(136)-H(136)	0.9500
C(2)-N(2)	1.141(9)
N(2)-B(2)	1.577(10)
B(2)-C(231)	1.621(13)
B(2)-C(211)	1.626(12)
B(2)-C(221)	1.631(12)
C(211)-C(216)	1.398(13)
C(211)-C(212)	1.411(12)
C(212)-C(213)	1.384(17)
C(212)-H(212)	0.9500
C(213)-C(214)	1.34(2)
C(213)-H(213)	0.9500
C(214)-C(215)	1.392(18)
C(214)-H(214)	0.9500
C(215)-C(216)	1.384(14)
C(215)-H(215)	0.9500
C(216)-H(216)	0.9500
C(221)-C(226)	1.373(12)
C(221)-C(222)	1.403(12)
C(222)-C(223)	1.379(14)
C(222)-H(222)	0.9500
C(223)-C(224)	1.374(15)
C(223)-H(223)	0.9500
C(224)-C(225)	1.404(14)
C(224)-H(224)	0.9500
C(225)-C(226)	1.387(13)
C(225)-H(225)	0.9500
C(226)-H(226)	0.9500
C(231)-C(232)	1.400(11)
C(231)-C(236)	1.411(12)
C(232)-C(233)	1.385(13)
C(232)-H(232)	0.9500
C(233)-C(234)	1.391(14)
C(233)-H(233)	0.9500
C(234)-C(235)	1.368(12)
C(234)-H(234)	0.9500
C(235)-C(236)	1.388(12)
C(235)-H(235)	0.9500
C(236)-H(236)	0.9500
C(3)-N(3)	1.164(10)
N(3)-B(3)	1.573(10)
B(3)-C(331)	1.626(14)
B(3)-C(311)	1.635(13)
B(3)-C(321)	1.643(13)
C(311)-C(316)	1.382(12)
C(311)-C(312)	1.397(12)
C(312)-C(313)	1.398(16)
C(312)-H(312)	0.9500
C(313)-C(314)	1.377(17)
C(313)-H(313)	0.9500
C(314)-C(315)	1.351(15)
C(314)-H(314)	0.9500
C(315)-C(316)	1.398(13)
C(315)-H(315)	0.9500
C(316)-H(316)	0.9500
C(321)-C(326)	1.382(15)
C(321)-C(322)	1.403(15)
C(322)-C(323)	1.417(15)
C(322)-H(322)	0.9500
C(323)-C(324)	1.37(2)
C(323)-H(323)	0.9500
C(324)-C(325)	1.39(2)
C(324)-H(324)	0.9500
C(325)-C(326)	1.417(14)
C(325)-H(325)	0.9500
C(326)-H(326)	0.9500
C(331)-C(336)	1.381(12)
C(331)-C(332)	1.406(12)
C(332)-C(333)	1.389(15)
C(332)-H(332)	0.9500
C(333)-C(334)	1.369(17)
C(333)-H(333)	0.9500
C(334)-C(335)	1.389(14)
C(334)-H(334)	0.9500
C(335)-C(336)	1.380(12)
C(335)-H(335)	0.9500
C(336)-H(336)	0.9500
C(4)-N(4)	1.166(10)
N(4)-B(4)	1.557(10)
B(4)-C(431)	1.627(12)
B(4)-C(411)	1.635(12)
B(4)-C(421)	1.636(11)
C(411)-C(416)	1.397(11)
C(411)-C(412)	1.404(11)
C(412)-C(413)	1.384(11)
C(412)-H(412)	0.9500
C(413)-C(414)	1.385(13)
C(413)-H(413)	0.9500
C(414)-C(415)	1.383(13)

C(414)-H(414)	0.9500	C(523)-C(524)	1.360(18)
C(415)-C(416)	1.381(11)	C(523)-H(523)	0.9500
C(415)-H(415)	0.9500	C(524)-C(525)	1.365(18)
C(416)-H(416)	0.9500	C(524)-H(524)	0.9500
C(421)-C(426)	1.369(12)	C(525)-C(526)	1.401(17)
C(421)-C(422)	1.403(12)	C(525)-H(525)	0.9500
C(422)-C(423)	1.392(13)	C(526)-H(526)	0.9500
C(422)-H(422)	0.9500	C(531)-C(536)	1.389(16)
C(423)-C(424)	1.351(17)	C(531)-C(532)	1.393(16)
C(423)-H(423)	0.9500	C(532)-C(533)	1.391(17)
C(424)-C(425)	1.404(16)	C(532)-H(532)	0.9500
C(424)-H(424)	0.9500	C(533)-C(534)	1.381(18)
C(425)-C(426)	1.390(13)	C(533)-H(533)	0.9500
C(425)-H(425)	0.9500	C(534)-C(535)	1.376(17)
C(426)-H(426)	0.9500	C(534)-H(534)	0.9500
C(431)-C(432)	1.401(13)	C(535)-C(536)	1.394(17)
C(431)-C(436)	1.410(12)	C(535)-H(535)	0.9500
C(432)-C(433)	1.394(13)	C(536)-H(536)	0.9500
C(432)-H(432)	0.9500	C(5E)-N(5E)	1.166(17)
C(433)-C(434)	1.380(16)	N(5E)-B(5E)	1.568(16)
C(433)-H(433)	0.9500	B(5E)-C(11E)	1.617(16)
C(434)-C(435)	1.378(16)	B(5E)-C(21E)	1.625(16)
C(434)-H(434)	0.9500	B(5E)-C(31E)	1.638(16)
C(435)-C(436)	1.391(13)	C(11E)-C(16E)	1.381(16)
C(435)-H(435)	0.9500	C(11E)-C(12E)	1.392(16)
C(436)-H(436)	0.9500	C(12E)-C(13E)	1.394(18)
C(5)-N(5)	1.181(16)	C(12E)-H(12E)	0.9500
N(5)-B(5)	1.572(16)	C(13E)-C(14E)	1.373(17)
B(5)-C(511)	1.613(16)	C(13E)-H(13E)	0.9500
B(5)-C(531)	1.628(16)	C(14E)-C(15E)	1.372(17)
B(5)-C(521)	1.635(15)	C(14E)-H(14E)	0.9500
C(511)-C(516)	1.379(17)	C(15E)-C(16E)	1.407(17)
C(511)-C(512)	1.398(16)	C(15E)-H(15E)	0.9500
C(512)-C(513)	1.388(17)	C(16E)-H(16E)	0.9500
C(512)-H(512)	0.9500	C(21E)-C(22E)	1.387(16)
C(513)-C(514)	1.371(18)	C(21E)-C(26E)	1.393(17)
C(513)-H(513)	0.9500	C(22E)-C(23E)	1.403(17)
C(514)-C(515)	1.377(18)	C(22E)-H(22E)	0.9500
C(514)-H(514)	0.9500	C(23E)-C(24E)	1.371(18)
C(515)-C(516)	1.409(17)	C(23E)-H(23E)	0.9500
C(515)-H(515)	0.9500	C(24E)-C(25E)	1.373(17)
C(516)-H(516)	0.9500	C(24E)-H(24E)	0.9500
C(521)-C(526)	1.365(16)	C(25E)-C(26E)	1.395(17)
C(521)-C(522)	1.397(16)	C(25E)-H(25E)	0.9500
C(522)-C(523)	1.408(17)	C(26E)-H(26E)	0.9500
C(522)-H(522)	0.9500	C(31E)-C(36E)	1.386(17)

C(31E)-C(32E)	1.389(16)	C(635)-H(635)	0.9500
C(32E)-C(33E)	1.406(17)	C(636)-H(636)	0.9500
C(32E)-H(32E)	0.9500	C(6F)-N(6F)	1.191(17)
C(33E)-C(34E)	1.380(18)	N(6F)-B(6F)	1.579(17)
C(33E)-H(33E)	0.9500	B(6F)-C(21F)	1.632(17)
C(34E)-C(35E)	1.363(18)	B(6F)-C(11F)	1.633(17)
C(34E)-H(34E)	0.9500	B(6F)-C(31F)	1.637(16)
C(35E)-C(36E)	1.402(17)	C(11F)-C(12F)	1.388(18)
C(35E)-H(35E)	0.9500	C(11F)-C(16F)	1.392(17)
C(36E)-H(36E)	0.9500	C(12F)-C(13F)	1.390(18)
C(6)-N(6)	1.168(14)	C(12F)-H(12F)	0.9500
N(6)-B(6)	1.550(15)	C(13F)-C(14F)	1.373(19)
B(6)-C(621)	1.628(15)	C(13F)-H(13F)	0.9500
B(6)-C(631)	1.637(15)	C(14F)-C(15F)	1.374(19)
B(6)-C(611)	1.644(15)	C(14F)-H(14F)	0.9500
C(611)-C(616)	1.387(15)	C(15F)-C(16F)	1.402(18)
C(611)-C(612)	1.403(16)	C(15F)-H(15F)	0.9500
C(612)-C(613)	1.399(16)	C(16F)-H(16F)	0.9500
C(612)-H(612)	0.9500	C(21F)-C(22F)	1.399(17)
C(613)-C(614)	1.377(16)	C(21F)-C(26F)	1.403(18)
C(613)-H(613)	0.9500	C(22F)-C(23F)	1.389(19)
C(614)-C(615)	1.359(18)	C(22F)-H(22F)	0.9500
C(614)-H(614)	0.9500	C(23F)-C(24F)	1.389(19)
C(615)-C(616)	1.410(17)	C(23F)-H(23F)	0.9500
C(615)-H(615)	0.9500	C(24F)-C(25F)	1.370(18)
C(616)-H(616)	0.9500	C(24F)-H(24F)	0.9500
C(621)-C(622)	1.391(15)	C(25F)-C(26F)	1.406(18)
C(621)-C(626)	1.400(17)	C(25F)-H(25F)	0.9500
C(622)-C(623)	1.393(17)	C(26F)-H(26F)	0.9500
C(622)-H(622)	0.9500	C(31F)-C(36F)	1.384(17)
C(623)-C(624)	1.372(18)	C(31F)-C(32F)	1.389(17)
C(623)-H(623)	0.9500	C(32F)-C(33F)	1.400(18)
C(624)-C(625)	1.364(18)	C(32F)-H(32F)	0.9500
C(624)-H(624)	0.9500	C(33F)-C(34F)	1.367(18)
C(625)-C(626)	1.399(17)	C(33F)-H(33F)	0.9500
C(625)-H(625)	0.9500	C(34F)-C(35F)	1.369(18)
C(626)-H(626)	0.9500	C(34F)-H(34F)	0.9500
C(631)-C(632)	1.383(15)	C(35F)-C(36F)	1.396(18)
C(631)-C(636)	1.391(15)	C(35F)-H(35F)	0.9500
C(632)-C(633)	1.414(15)	C(36F)-H(36F)	0.9500
C(632)-H(632)	0.9500	N(1A)-C(11A)	1.512(13)
C(633)-C(634)	1.384(16)	N(1A)-C(17A)	1.513(14)
C(633)-H(633)	0.9500	N(1A)-C(15A)	1.514(14)
C(634)-C(635)	1.369(16)	N(1A)-C(13A)	1.515(14)
C(634)-H(634)	0.9500	C(11A)-C(12A)	1.507(18)
C(635)-C(636)	1.392(15)	C(11A)-H(11A)	0.9900

C(11A)-H(11B)	0.9900	C(27A)-H(27B)	0.9900
C(12A)-H(12A)	0.9800	C(28A)-H(28A)	0.9800
C(12A)-H(12B)	0.9800	C(28A)-H(28B)	0.9800
C(12A)-H(12C)	0.9800	C(28A)-H(28C)	0.9800
C(13A)-C(14A)	1.503(16)	N(3A)-C(31A)	1.509(13)
C(13A)-H(13A)	0.9900	N(3A)-C(33A)	1.514(14)
C(13A)-H(13B)	0.9900	N(3A)-C(35A)	1.522(12)
C(14A)-H(14A)	0.9800	N(3A)-C(37A)	1.526(14)
C(14A)-H(14B)	0.9800	C(31A)-C(32A)	1.494(14)
C(14A)-H(14C)	0.9800	C(31A)-H(31A)	0.9900
C(15A)-C(16A)	1.505(16)	C(31A)-H(31B)	0.9900
C(15A)-H(15A)	0.9900	C(32A)-H(32A)	0.9800
C(15A)-H(15B)	0.9900	C(32A)-H(32B)	0.9800
C(16A)-H(16A)	0.9800	C(32A)-H(32C)	0.9800
C(16A)-H(16B)	0.9800	C(33A)-C(34A)	1.502(16)
C(16A)-H(16C)	0.9800	C(33A)-H(33A)	0.9900
C(17A)-C(18A)	1.527(16)	C(33A)-H(33B)	0.9900
C(17A)-H(17A)	0.9900	C(34A)-H(34A)	0.9800
C(17A)-H(17B)	0.9900	C(34A)-H(34B)	0.9800
C(18A)-H(18A)	0.9800	C(34A)-H(34C)	0.9800
C(18A)-H(18B)	0.9800	C(35A)-C(36A)	1.496(15)
C(18A)-H(18C)	0.9800	C(35A)-H(35A)	0.9900
N(2A)-C(27A)	1.505(15)	C(35A)-H(35B)	0.9900
N(2A)-C(21A)	1.519(15)	C(36A)-H(36A)	0.9800
N(2A)-C(25A)	1.521(15)	C(36A)-H(36B)	0.9800
N(2A)-C(23A)	1.523(15)	C(36A)-H(36C)	0.9800
C(21A)-C(22A)	1.507(18)	C(37A)-C(38A)	1.478(16)
C(21A)-H(21A)	0.9900	C(37A)-H(37A)	0.9900
C(21A)-H(21B)	0.9900	C(37A)-H(37B)	0.9900
C(22A)-H(22A)	0.9800	C(38A)-H(38A)	0.9800
C(22A)-H(22B)	0.9800	C(38A)-H(38B)	0.9800
C(22A)-H(22C)	0.9800	C(38A)-H(38C)	0.9800
C(23A)-C(24A)	1.493(17)	N(4A)-C(43A)	1.501(19)
C(23A)-H(23A)	0.9900	N(4A)-C(47A)	1.511(19)
C(23A)-H(23B)	0.9900	N(4A)-C(45A)	1.515(19)
C(24A)-H(24A)	0.9800	N(4A)-C(41A)	1.517(19)
C(24A)-H(24B)	0.9800	C(41A)-C(42A)	1.50(2)
C(24A)-H(24C)	0.9800	C(41A)-H(41A)	0.9900
C(25A)-C(26A)	1.514(18)	C(41A)-H(41B)	0.9900
C(25A)-H(25A)	0.9900	C(42A)-H(42A)	0.9800
C(25A)-H(25B)	0.9900	C(42A)-H(42B)	0.9800
C(26A)-H(26A)	0.9800	C(42A)-H(42C)	0.9800
C(26A)-H(26B)	0.9800	C(43A)-C(44A)	1.50(2)
C(26A)-H(26C)	0.9800	C(43A)-H(43A)	0.9900
C(27A)-C(28A)	1.506(18)	C(43A)-H(43B)	0.9900
C(27A)-H(27A)	0.9900	C(44A)-H(44A)	0.9800

C(44A)-H(44B)	0.9800	C(21B)-C(22B)	1.500(19)
C(44A)-H(44C)	0.9800	C(21B)-H(21C)	0.9900
C(45A)-C(46A)	1.51(2)	C(21B)-H(21D)	0.9900
C(45A)-H(45A)	0.9900	C(22B)-H(22D)	0.9800
C(45A)-H(45B)	0.9900	C(22B)-H(22G)	0.9800
C(46A)-H(46A)	0.9800	C(22B)-H(22H)	0.9800
C(46A)-H(46B)	0.9800	C(23B)-C(24B)	1.495(19)
C(46A)-H(46C)	0.9800	C(23B)-H(23C)	0.9900
C(47A)-C(48A)	1.50(2)	C(23B)-H(23D)	0.9900
C(47A)-H(47A)	0.9900	C(24B)-H(24D)	0.9800
C(47A)-H(47B)	0.9900	C(24B)-H(24G)	0.9800
C(48A)-H(48A)	0.9800	C(24B)-H(24H)	0.9800
C(48A)-H(48B)	0.9800	C(25B)-C(26B)	1.490(19)
C(48A)-H(48C)	0.9800	C(25B)-H(25C)	0.9900
N(1B)-C(13B)	1.513(18)	C(25B)-H(25D)	0.9900
N(1B)-C(17B)	1.514(18)	C(26B)-H(26D)	0.9800
N(1B)-C(11B)	1.515(18)	C(26B)-H(26G)	0.9800
N(1B)-C(15B)	1.519(18)	C(26B)-H(26H)	0.9800
C(11B)-C(12B)	1.50(2)	C(27B)-C(28B)	1.500(19)
C(11B)-H(11C)	0.9900	C(27B)-H(27C)	0.9900
C(11B)-H(11D)	0.9900	C(27B)-H(27D)	0.9900
C(12B)-H(12D)	0.9800	C(28B)-H(28D)	0.9800
C(12B)-H(12G)	0.9800	C(28B)-H(28E)	0.9800
C(12B)-H(12H)	0.9800	C(28B)-H(28F)	0.9800
C(13B)-C(14B)	1.50(2)	N(3B)-C(31B)	1.516(19)
C(13B)-H(13C)	0.9900	N(3B)-C(35B)	1.516(19)
C(13B)-H(13D)	0.9900	N(3B)-C(33B)	1.517(19)
C(14B)-H(14D)	0.9800	N(3B)-C(37B)	1.522(19)
C(14B)-H(14G)	0.9800	C(31B)-C(32B)	1.51(2)
C(14B)-H(14H)	0.9800	C(31B)-H(31C)	0.9900
C(15B)-C(16B)	1.49(2)	C(31B)-H(31D)	0.9900
C(15B)-H(15C)	0.9900	C(32B)-H(32D)	0.9800
C(15B)-H(15D)	0.9900	C(32B)-H(32G)	0.9800
C(16B)-H(16D)	0.9800	C(32B)-H(32H)	0.9800
C(16B)-H(16G)	0.9800	C(33B)-C(34B)	1.50(2)
C(16B)-H(16H)	0.9800	C(33B)-H(33C)	0.9900
C(17B)-C(18B)	1.50(2)	C(33B)-H(33D)	0.9900
C(17B)-H(17C)	0.9900	C(34B)-H(34D)	0.9800
C(17B)-H(17D)	0.9900	C(34B)-H(34G)	0.9800
C(18B)-H(18D)	0.9800	C(34B)-H(34H)	0.9800
C(18B)-H(18E)	0.9800	C(35B)-C(36B)	1.50(2)
C(18B)-H(18F)	0.9800	C(35B)-H(35C)	0.9900
N(2B)-C(25B)	1.515(17)	C(35B)-H(35D)	0.9900
N(2B)-C(21B)	1.517(17)	C(36B)-H(36D)	0.9800
N(2B)-C(27B)	1.520(17)	C(36B)-H(36G)	0.9800
N(2B)-C(23B)	1.525(17)	C(36B)-H(36H)	0.9800

C(37B)-C(38B)	1.49(2)
C(37B)-H(37C)	0.9900
C(37B)-H(37D)	0.9900
C(38B)-H(38D)	0.9800
C(38B)-H(38E)	0.9800
C(38B)-H(38F)	0.9800
N(4B)-C(43B)	1.485(18)
N(4B)-C(47B)	1.499(18)
N(4B)-C(45B)	1.510(18)
N(4B)-C(41B)	1.549(19)
C(41B)-C(42B)	1.49(2)
C(41B)-H(41C)	0.9900
C(41B)-H(41D)	0.9900
C(42B)-H(42D)	0.9800
C(42B)-H(42E)	0.9800
C(42B)-H(42F)	0.9800
C(43B)-C(44B)	1.48(2)
C(43B)-H(43C)	0.9900
C(43B)-H(43D)	0.9900
C(44B)-H(44D)	0.9800
C(44B)-H(44E)	0.9800
C(44B)-H(44F)	0.9800
C(45B)-C(46B)	1.48(2)
C(45B)-H(45C)	0.9900
C(45B)-H(45D)	0.9900
C(46B)-H(46D)	0.9800
C(46B)-H(46E)	0.9800
C(46B)-H(46F)	0.9800
C(47B)-C(48B)	1.49(2)
C(47B)-H(47C)	0.9900
C(47B)-H(47D)	0.9900
C(48B)-H(48D)	0.9800
C(48B)-H(48E)	0.9800
C(48B)-H(48F)	0.9800
C(1S)-Cl(1S)	1.667(19)
C(1S)-Cl(2S)	1.690(19)
C(1S)-H(1S1)	0.9900
C(1S)-H(1S2)	0.9900
C(2T)-Cl(3T)	1.72(2)
C(2T)-Cl(4T)	1.72(2)
C(2T)-H(2T1)	0.9900
C(2T)-H(2T2)	0.9900
C(2U)-Cl(4U)	1.73(2)
C(2U)-Cl(3U)	1.73(2)
C(2U)-H(2U1)	0.9900
C(2U)-H(2U2)	0.9900
C(2S)-Cl(3S)	1.73(2)
C(2S)-Cl(4S)	1.74(2)
C(2S)-H(2S1)	0.9900
C(2S)-H(2S2)	0.9900
C(3S)-Cl(5S)	1.70(2)
C(3S)-Cl(6S)	1.75(2)
C(3S)-H(3S1)	0.9900
C(3S)-H(3S2)	0.9900
C(3T)-Cl(5T)	1.74(2)
C(3T)-Cl(6T)	1.74(2)
C(3T)-H(3T1)	0.9900
C(3T)-H(3T2)	0.9900

C(6)-Fe(1)-C(4)	179.1(11)
C(6)-Fe(1)-C(1)	92.7(16)
C(4)-Fe(1)-C(1)	87.0(3)
C(6)-Fe(1)-C(5)	87(3)
C(4)-Fe(1)-C(5)	92(2)
C(1)-Fe(1)-C(5)	84.9(16)
C(6)-Fe(1)-C(3)	89.2(10)
C(4)-Fe(1)-C(3)	91.6(3)
C(1)-Fe(1)-C(3)	92.4(3)
C(5)-Fe(1)-C(3)	175(2)
C(5E)-Fe(1)-C(6F)	93(3)
C(6)-Fe(1)-C(2)	93.3(16)
C(4)-Fe(1)-C(2)	87.0(3)
C(1)-Fe(1)-C(2)	173.4(3)
C(5)-Fe(1)-C(2)	92.6(16)
C(3)-Fe(1)-C(2)	90.5(3)
N(1)-C(1)-Fe(1)	174.0(7)
C(1)-N(1)-B(1)	172.0(8)
N(1)-B(1)-C(131)	106.9(6)
N(1)-B(1)-C(111)	106.0(7)
C(131)-B(1)-C(111)	116.8(6)
N(1)-B(1)-C(121)	108.4(6)
C(131)-B(1)-C(121)	108.4(7)
C(111)-B(1)-C(121)	110.0(6)
C(112)-C(111)-C(116)	115.0(9)
C(112)-C(111)-B(1)	125.5(9)
C(116)-C(111)-B(1)	119.4(7)
C(111)-C(112)-C(113)	123.4(12)
C(111)-C(112)-H(112)	118.3
C(113)-C(112)-H(112)	118.3
C(114)-C(113)-C(112)	120.2(11)
C(114)-C(113)-H(113)	119.9
C(112)-C(113)-H(113)	119.9

C(115)-C(114)-C(113)	118.8(11)	C(2)-N(2)-B(2)	168.5(7)
C(115)-C(114)-H(114)	120.6	N(2)-B(2)-C(231)	107.1(7)
C(113)-C(114)-H(114)	120.6	N(2)-B(2)-C(211)	108.6(6)
C(114)-C(115)-C(116)	118.4(12)	C(231)-B(2)-C(211)	107.9(7)
C(114)-C(115)-H(115)	120.8	N(2)-B(2)-C(221)	107.0(6)
C(116)-C(115)-H(115)	120.8	C(231)-B(2)-C(221)	111.9(7)
C(111)-C(116)-C(115)	124.1(11)	C(211)-B(2)-C(221)	114.2(7)
C(111)-C(116)-H(116)	118.0	C(216)-C(211)-C(212)	115.4(8)
C(115)-C(116)-H(116)	118.0	C(216)-C(211)-B(2)	121.6(7)
C(126)-C(121)-C(122)	115.7(8)	C(212)-C(211)-B(2)	122.0(8)
C(126)-C(121)-B(1)	123.8(7)	C(213)-C(212)-C(211)	121.6(12)
C(122)-C(121)-B(1)	120.4(7)	C(213)-C(212)-H(212)	119.2
C(121)-C(122)-C(123)	122.8(9)	C(211)-C(212)-H(212)	119.2
C(121)-C(122)-H(122)	118.6	C(214)-C(213)-C(212)	121.0(12)
C(123)-C(122)-H(122)	118.6	C(214)-C(213)-H(213)	119.5
C(124)-C(123)-C(122)	119.7(10)	C(212)-C(213)-H(213)	119.5
C(124)-C(123)-H(123)	120.1	C(213)-C(214)-C(215)	120.3(11)
C(122)-C(123)-H(123)	120.1	C(213)-C(214)-H(214)	119.8
C(125)-C(124)-C(123)	118.9(10)	C(215)-C(214)-H(214)	119.8
C(125)-C(124)-H(124)	120.6	C(216)-C(215)-C(214)	118.9(12)
C(123)-C(124)-H(124)	120.6	C(216)-C(215)-H(215)	120.6
C(124)-C(125)-C(126)	120.9(9)	C(214)-C(215)-H(215)	120.6
C(124)-C(125)-H(125)	119.5	C(215)-C(216)-C(211)	122.7(9)
C(126)-C(125)-H(125)	119.5	C(215)-C(216)-H(216)	118.6
C(121)-C(126)-C(125)	122.0(8)	C(211)-C(216)-H(216)	118.6
C(121)-C(126)-H(126)	119.0	C(226)-C(221)-C(222)	116.3(8)
C(125)-C(126)-H(126)	119.0	C(226)-C(221)-B(2)	124.1(7)
C(136)-C(131)-C(132)	113.2(8)	C(222)-C(221)-B(2)	119.6(8)
C(136)-C(131)-B(1)	120.7(6)	C(223)-C(222)-C(221)	121.9(10)
C(132)-C(131)-B(1)	125.4(8)	C(223)-C(222)-H(222)	119.1
C(131)-C(132)-C(133)	123.5(9)	C(221)-C(222)-H(222)	119.1
C(131)-C(132)-H(132)	118.3	C(224)-C(223)-C(222)	120.9(9)
C(133)-C(132)-H(132)	118.3	C(224)-C(223)-H(223)	119.6
C(134)-C(133)-C(132)	120.9(9)	C(222)-C(223)-H(223)	119.6
C(134)-C(133)-H(133)	119.6	C(223)-C(224)-C(225)	118.5(9)
C(132)-C(133)-H(133)	119.6	C(223)-C(224)-H(224)	120.7
C(133)-C(134)-C(135)	117.9(10)	C(225)-C(224)-H(224)	120.7
C(133)-C(134)-H(134)	121.0	C(226)-C(225)-C(224)	119.4(9)
C(135)-C(134)-H(134)	121.0	C(226)-C(225)-H(225)	120.3
C(134)-C(135)-C(136)	120.9(9)	C(224)-C(225)-H(225)	120.3
C(134)-C(135)-H(135)	119.6	C(221)-C(226)-C(225)	123.0(8)
C(136)-C(135)-H(135)	119.6	C(221)-C(226)-H(226)	118.5
C(131)-C(136)-C(135)	123.6(8)	C(225)-C(226)-H(226)	118.5
C(131)-C(136)-H(136)	118.2	C(232)-C(231)-C(236)	114.8(8)
C(135)-C(136)-H(136)	118.2	C(232)-C(231)-B(2)	122.1(7)
N(2)-C(2)-Fe(1)	175.2(7)	C(236)-C(231)-B(2)	123.1(7)

C(233)-C(232)-C(231)	122.8(9)	C(323)-C(322)-H(322)	119.5
C(233)-C(232)-H(232)	118.6	C(324)-C(323)-C(322)	120.6(13)
C(231)-C(232)-H(232)	118.6	C(324)-C(323)-H(323)	119.7
C(232)-C(233)-C(234)	121.2(8)	C(322)-C(323)-H(323)	119.7
C(232)-C(233)-H(233)	119.4	C(323)-C(324)-C(325)	120.0(11)
C(234)-C(233)-H(233)	119.4	C(323)-C(324)-H(324)	120.0
C(235)-C(234)-C(233)	117.2(9)	C(325)-C(324)-H(324)	120.0
C(235)-C(234)-H(234)	121.4	C(324)-C(325)-C(326)	118.7(13)
C(233)-C(234)-H(234)	121.4	C(324)-C(325)-H(325)	120.7
C(234)-C(235)-C(236)	122.2(9)	C(326)-C(325)-H(325)	120.7
C(234)-C(235)-H(235)	118.9	C(321)-C(326)-C(325)	122.8(11)
C(236)-C(235)-H(235)	118.9	C(321)-C(326)-H(326)	118.6
C(235)-C(236)-C(231)	121.9(7)	C(325)-C(326)-H(326)	118.6
C(235)-C(236)-H(236)	119.1	C(336)-C(331)-C(332)	115.1(8)
C(231)-C(236)-H(236)	119.1	C(336)-C(331)-B(3)	124.0(7)
N(3)-C(3)-Fe(1)	174.0(7)	C(332)-C(331)-B(3)	120.7(8)
C(3)-N(3)-B(3)	173.5(8)	C(333)-C(332)-C(331)	121.7(9)
N(3)-B(3)-C(331)	107.0(7)	C(333)-C(332)-H(332)	119.1
N(3)-B(3)-C(311)	106.3(6)	C(331)-C(332)-H(332)	119.1
C(331)-B(3)-C(311)	113.9(7)	C(334)-C(333)-C(332)	121.4(9)
N(3)-B(3)-C(321)	108.2(7)	C(334)-C(333)-H(333)	119.3
C(331)-B(3)-C(321)	110.3(7)	C(332)-C(333)-H(333)	119.3
C(311)-B(3)-C(321)	110.9(7)	C(333)-C(334)-C(335)	118.0(10)
C(316)-C(311)-C(312)	116.0(8)	C(333)-C(334)-H(334)	121.0
C(316)-C(311)-B(3)	121.7(7)	C(335)-C(334)-H(334)	121.0
C(312)-C(311)-B(3)	122.3(8)	C(336)-C(335)-C(334)	120.1(10)
C(311)-C(312)-C(313)	121.7(10)	C(336)-C(335)-H(335)	120.0
C(311)-C(312)-H(312)	119.1	C(334)-C(335)-H(335)	120.0
C(313)-C(312)-H(312)	119.1	C(335)-C(336)-C(331)	123.6(8)
C(314)-C(313)-C(312)	120.3(10)	C(335)-C(336)-H(336)	118.2
C(314)-C(313)-H(313)	119.8	C(331)-C(336)-H(336)	118.2
C(312)-C(313)-H(313)	119.8	N(4)-C(4)-Fe(1)	179.6(8)
C(315)-C(314)-C(313)	118.9(10)	C(4)-N(4)-B(4)	176.3(7)
C(315)-C(314)-H(314)	120.5	N(4)-B(4)-C(431)	108.3(6)
C(313)-C(314)-H(314)	120.5	N(4)-B(4)-C(411)	107.5(6)
C(314)-C(315)-C(316)	121.0(10)	C(431)-B(4)-C(411)	112.4(7)
C(314)-C(315)-H(315)	119.5	N(4)-B(4)-C(421)	107.0(6)
C(316)-C(315)-H(315)	119.5	C(431)-B(4)-C(421)	110.4(6)
C(311)-C(316)-C(315)	122.0(8)	C(411)-B(4)-C(421)	111.0(6)
C(311)-C(316)-H(316)	119.0	C(416)-C(411)-C(412)	114.8(7)
C(315)-C(316)-H(316)	119.0	C(416)-C(411)-B(4)	122.1(7)
C(326)-C(321)-C(322)	116.8(9)	C(412)-C(411)-B(4)	123.0(7)
C(326)-C(321)-B(3)	123.0(8)	C(413)-C(412)-C(411)	123.2(7)
C(322)-C(321)-B(3)	120.2(9)	C(413)-C(412)-H(412)	118.4
C(321)-C(322)-C(323)	121.0(12)	C(411)-C(412)-H(412)	118.4
C(321)-C(322)-H(322)	119.5	C(412)-C(413)-C(414)	119.6(8)

C(412)-C(413)-H(413)	120.2	C(431)-C(436)-H(436)	118.7
C(414)-C(413)-H(413)	120.2	N(5)-C(5)-Fe(1)	176(4)
C(415)-C(414)-C(413)	119.3(8)	C(5)-N(5)-B(5)	165(4)
C(415)-C(414)-H(414)	120.3	N(5)-B(5)-C(511)	107.5(14)
C(413)-C(414)-H(414)	120.3	N(5)-B(5)-C(531)	108.7(15)
C(416)-C(415)-C(414)	119.8(8)	C(511)-B(5)-C(531)	114.4(14)
C(416)-C(415)-H(415)	120.1	N(5)-B(5)-C(521)	110.1(16)
C(414)-C(415)-H(415)	120.1	C(511)-B(5)-C(521)	108.4(14)
C(415)-C(416)-C(411)	123.3(8)	C(531)-B(5)-C(521)	107.7(14)
C(415)-C(416)-H(416)	118.4	C(516)-C(511)-C(512)	116.8(15)
C(411)-C(416)-H(416)	118.4	C(516)-C(511)-B(5)	122.6(16)
C(426)-C(421)-C(422)	116.4(8)	C(512)-C(511)-B(5)	120.6(15)
C(426)-C(421)-B(4)	122.2(7)	C(513)-C(512)-C(511)	122.2(17)
C(422)-C(421)-B(4)	121.4(8)	C(513)-C(512)-H(512)	118.9
C(423)-C(422)-C(421)	121.3(10)	C(511)-C(512)-H(512)	118.9
C(423)-C(422)-H(422)	119.3	C(514)-C(513)-C(512)	119.5(18)
C(421)-C(422)-H(422)	119.3	C(514)-C(513)-H(513)	120.3
C(424)-C(423)-C(422)	121.3(10)	C(512)-C(513)-H(513)	120.3
C(424)-C(423)-H(423)	119.4	C(513)-C(514)-C(515)	120.3(18)
C(422)-C(423)-H(423)	119.4	C(513)-C(514)-H(514)	119.8
C(423)-C(424)-C(425)	118.7(9)	C(515)-C(514)-H(514)	119.8
C(423)-C(424)-H(424)	120.6	C(514)-C(515)-C(516)	119.3(18)
C(425)-C(424)-H(424)	120.6	C(514)-C(515)-H(515)	120.3
C(426)-C(425)-C(424)	119.4(10)	C(516)-C(515)-H(515)	120.3
C(426)-C(425)-H(425)	120.3	C(511)-C(516)-C(515)	121.7(18)
C(424)-C(425)-H(425)	120.3	C(511)-C(516)-H(516)	119.2
C(421)-C(426)-C(425)	122.8(9)	C(515)-C(516)-H(516)	119.2
C(421)-C(426)-H(426)	118.6	C(526)-C(521)-C(522)	114.9(14)
C(425)-C(426)-H(426)	118.6	C(526)-C(521)-B(5)	122.0(14)
C(432)-C(431)-C(436)	115.5(8)	C(522)-C(521)-B(5)	122.8(14)
C(432)-C(431)-B(4)	121.3(7)	C(521)-C(522)-C(523)	122.3(16)
C(436)-C(431)-B(4)	123.2(8)	C(521)-C(522)-H(522)	118.9
C(433)-C(432)-C(431)	121.9(9)	C(523)-C(522)-H(522)	118.9
C(433)-C(432)-H(432)	119.0	C(524)-C(523)-C(522)	119.3(17)
C(431)-C(432)-H(432)	119.0	C(524)-C(523)-H(523)	120.3
C(434)-C(433)-C(432)	120.6(10)	C(522)-C(523)-H(523)	120.3
C(434)-C(433)-H(433)	119.7	C(523)-C(524)-C(525)	120.5(17)
C(432)-C(433)-H(433)	119.7	C(523)-C(524)-H(524)	119.7
C(435)-C(434)-C(433)	119.4(10)	C(525)-C(524)-H(524)	119.7
C(435)-C(434)-H(434)	120.3	C(524)-C(525)-C(526)	118.4(17)
C(433)-C(434)-H(434)	120.3	C(524)-C(525)-H(525)	120.8
C(434)-C(435)-C(436)	119.9(10)	C(526)-C(525)-H(525)	120.8
C(434)-C(435)-H(435)	120.0	C(521)-C(526)-C(525)	124.3(17)
C(436)-C(435)-H(435)	120.0	C(521)-C(526)-H(526)	117.9
C(435)-C(436)-C(431)	122.6(9)	C(525)-C(526)-H(526)	117.9
C(435)-C(436)-H(436)	118.7	C(536)-C(531)-C(532)	115.0(15)

C(536)-C(531)-B(5)	122.9(15)	C(21E)-C(22E)-C(23E)	122.5(17)
C(532)-C(531)-B(5)	122.1(15)	C(21E)-C(22E)-H(22E)	118.7
C(533)-C(532)-C(531)	123.9(17)	C(23E)-C(22E)-H(22E)	118.7
C(533)-C(532)-H(532)	118.1	C(24E)-C(23E)-C(22E)	119.3(17)
C(531)-C(532)-H(532)	118.1	C(24E)-C(23E)-H(23E)	120.3
C(534)-C(533)-C(532)	119.0(18)	C(22E)-C(23E)-H(23E)	120.3
C(534)-C(533)-H(533)	120.5	C(23E)-C(24E)-C(25E)	119.6(17)
C(532)-C(533)-H(533)	120.5	C(23E)-C(24E)-H(24E)	120.2
C(535)-C(534)-C(533)	119.2(18)	C(25E)-C(24E)-H(24E)	120.2
C(535)-C(534)-H(534)	120.4	C(24E)-C(25E)-C(26E)	120.0(18)
C(533)-C(534)-H(534)	120.4	C(24E)-C(25E)-H(25E)	120.0
C(534)-C(535)-C(536)	120.6(19)	C(26E)-C(25E)-H(25E)	120.0
C(534)-C(535)-H(535)	119.7	C(21E)-C(26E)-C(25E)	122.3(16)
C(536)-C(535)-H(535)	119.7	C(21E)-C(26E)-H(26E)	118.9
C(531)-C(536)-C(535)	122.3(18)	C(25E)-C(26E)-H(26E)	118.9
C(531)-C(536)-H(536)	118.8	C(36E)-C(31E)-C(32E)	116.8(16)
C(535)-C(536)-H(536)	118.8	C(36E)-C(31E)-B(5E)	122.2(15)
N(5E)-C(5E)-Fe(1)	167(6)	C(32E)-C(31E)-B(5E)	120.8(15)
C(5E)-N(5E)-B(5E)	179(4)	C(31E)-C(32E)-C(33E)	122.0(18)
N(5E)-B(5E)-C(11E)	107.7(16)	C(31E)-C(32E)-H(32E)	119.0
N(5E)-B(5E)-C(21E)	106.6(16)	C(33E)-C(32E)-H(32E)	119.0
C(11E)-B(5E)-C(21E)	110.5(14)	C(34E)-C(33E)-C(32E)	118.5(18)
N(5E)-B(5E)-C(31E)	104.7(15)	C(34E)-C(33E)-H(33E)	120.8
C(11E)-B(5E)-C(31E)	115.4(15)	C(32E)-C(33E)-H(33E)	120.8
C(21E)-B(5E)-C(31E)	111.2(15)	C(35E)-C(34E)-C(33E)	121.5(19)
C(16E)-C(11E)-C(12E)	115.7(15)	C(35E)-C(34E)-H(34E)	119.2
C(16E)-C(11E)-B(5E)	123.6(15)	C(33E)-C(34E)-H(34E)	119.2
C(12E)-C(11E)-B(5E)	120.4(15)	C(34E)-C(35E)-C(36E)	118.7(19)
C(11E)-C(12E)-C(13E)	122.0(17)	C(34E)-C(35E)-H(35E)	120.7
C(11E)-C(12E)-H(12E)	119.0	C(36E)-C(35E)-H(35E)	120.7
C(13E)-C(12E)-H(12E)	119.0	C(31E)-C(36E)-C(35E)	122.3(18)
C(14E)-C(13E)-C(12E)	120.3(18)	C(31E)-C(36E)-H(36E)	118.8
C(14E)-C(13E)-H(13E)	119.9	C(35E)-C(36E)-H(36E)	118.8
C(12E)-C(13E)-H(13E)	119.9	N(6)-C(6)-Fe(1)	176(3)
C(15E)-C(14E)-C(13E)	119.9(18)	C(6)-N(6)-B(6)	173(3)
C(15E)-C(14E)-H(14E)	120.0	N(6)-B(6)-C(621)	105.9(12)
C(13E)-C(14E)-H(14E)	120.0	N(6)-B(6)-C(631)	107.8(12)
C(14E)-C(15E)-C(16E)	118.6(17)	C(621)-B(6)-C(631)	110.4(12)
C(14E)-C(15E)-H(15E)	120.7	N(6)-B(6)-C(611)	106.4(12)
C(16E)-C(15E)-H(15E)	120.7	C(621)-B(6)-C(611)	117.2(12)
C(11E)-C(16E)-C(15E)	123.4(17)	C(631)-B(6)-C(611)	108.6(12)
C(11E)-C(16E)-H(16E)	118.3	C(616)-C(611)-C(612)	116.2(13)
C(15E)-C(16E)-H(16E)	118.3	C(616)-C(611)-B(6)	121.7(13)
C(22E)-C(21E)-C(26E)	115.8(15)	C(612)-C(611)-B(6)	121.9(11)
C(22E)-C(21E)-B(5E)	123.9(15)	C(613)-C(612)-C(611)	121.5(14)
C(26E)-C(21E)-B(5E)	120.3(14)	C(613)-C(612)-H(612)	119.2

C(611)-C(612)-H(612)	119.2
C(614)-C(613)-C(612)	120.2(16)
C(614)-C(613)-H(613)	119.9
C(612)-C(613)-H(613)	119.9
C(615)-C(614)-C(613)	120.0(17)
C(615)-C(614)-H(614)	120.0
C(613)-C(614)-H(614)	120.0
C(614)-C(615)-C(616)	119.8(16)
C(614)-C(615)-H(615)	120.1
C(616)-C(615)-H(615)	120.1
C(611)-C(616)-C(615)	122.2(16)
C(611)-C(616)-H(616)	118.9
C(615)-C(616)-H(616)	118.9
C(622)-C(621)-C(626)	114.4(14)
C(622)-C(621)-B(6)	122.7(14)
C(626)-C(621)-B(6)	122.9(12)
C(621)-C(622)-C(623)	122.3(17)
C(621)-C(622)-H(622)	118.9
C(623)-C(622)-H(622)	118.9
C(624)-C(623)-C(622)	121.4(17)
C(624)-C(623)-H(623)	119.3
C(622)-C(623)-H(623)	119.3
C(625)-C(624)-C(623)	118.4(18)
C(625)-C(624)-H(624)	120.8
C(623)-C(624)-H(624)	120.8
C(624)-C(625)-C(626)	120.1(18)
C(624)-C(625)-H(625)	120.0
C(626)-C(625)-H(625)	120.0
C(625)-C(626)-C(621)	123.3(16)
C(625)-C(626)-H(626)	118.4
C(621)-C(626)-H(626)	118.4
C(632)-C(631)-C(636)	117.9(12)
C(632)-C(631)-B(6)	121.1(12)
C(636)-C(631)-B(6)	120.9(12)
C(631)-C(632)-C(633)	121.2(14)
C(631)-C(632)-H(632)	119.4
C(633)-C(632)-H(632)	119.4
C(634)-C(633)-C(632)	118.8(14)
C(634)-C(633)-H(633)	120.6
C(632)-C(633)-H(633)	120.6
C(635)-C(634)-C(633)	120.7(15)
C(635)-C(634)-H(634)	119.7
C(633)-C(634)-H(634)	119.7
C(634)-C(635)-C(636)	119.8(15)
C(634)-C(635)-H(635)	120.1
C(636)-C(635)-H(635)	120.1
C(631)-C(636)-C(635)	121.4(14)
C(631)-C(636)-H(636)	119.3
C(635)-C(636)-H(636)	119.3
N(6F)-C(6F)-Fe(1)	175(6)
C(6F)-N(6F)-B(6F)	171(4)
N(6F)-B(6F)-C(21F)	105.9(16)
N(6F)-B(6F)-C(11F)	106.7(16)
C(21F)-B(6F)-C(11F)	112.6(16)
N(6F)-B(6F)-C(31F)	109.5(16)
C(21F)-B(6F)-C(31F)	112.9(16)
C(11F)-B(6F)-C(31F)	108.9(16)
C(12F)-C(11F)-C(16F)	113.9(17)
C(12F)-C(11F)-B(6F)	122.8(16)
C(16F)-C(11F)-B(6F)	122.7(17)
C(11F)-C(12F)-C(13F)	124(2)
C(11F)-C(12F)-H(12F)	118.0
C(13F)-C(12F)-H(12F)	118.0
C(14F)-C(13F)-C(12F)	118(2)
C(14F)-C(13F)-H(13F)	121.1
C(12F)-C(13F)-H(13F)	121.1
C(13F)-C(14F)-C(15F)	122(2)
C(13F)-C(14F)-H(14F)	118.9
C(15F)-C(14F)-H(14F)	118.9
C(14F)-C(15F)-C(16F)	116(2)
C(14F)-C(15F)-H(15F)	121.8
C(16F)-C(15F)-H(15F)	121.8
C(11F)-C(16F)-C(15F)	125(2)
C(11F)-C(16F)-H(16F)	117.6
C(15F)-C(16F)-H(16F)	117.6
C(22F)-C(21F)-C(26F)	115.3(17)
C(22F)-C(21F)-B(6F)	123.9(17)
C(26F)-C(21F)-B(6F)	119.9(15)
C(23F)-C(22F)-C(21F)	124(2)
C(23F)-C(22F)-H(22F)	117.9
C(21F)-C(22F)-H(22F)	117.9
C(24F)-C(23F)-C(22F)	119(2)
C(24F)-C(23F)-H(23F)	120.7
C(22F)-C(23F)-H(23F)	120.7
C(25F)-C(24F)-C(23F)	119(2)
C(25F)-C(24F)-H(24F)	120.3
C(23F)-C(24F)-H(24F)	120.3
C(24F)-C(25F)-C(26F)	121(2)
C(24F)-C(25F)-H(25F)	119.4
C(26F)-C(25F)-H(25F)	119.4
C(21F)-C(26F)-C(25F)	121.0(18)
C(21F)-C(26F)-H(26F)	119.5

C(25F)-C(26F)-H(26F)	119.5
C(36F)-C(31F)-C(32F)	115.7(16)
C(36F)-C(31F)-B(6F)	123.0(16)
C(32F)-C(31F)-B(6F)	121.0(16)
C(31F)-C(32F)-C(33F)	122.1(19)
C(31F)-C(32F)-H(32F)	119.0
C(33F)-C(32F)-H(32F)	119.0
C(34F)-C(33F)-C(32F)	120(2)
C(34F)-C(33F)-H(33F)	120.2
C(32F)-C(33F)-H(33F)	120.2
C(33F)-C(34F)-C(35F)	119.8(19)
C(33F)-C(34F)-H(34F)	120.1
C(35F)-C(34F)-H(34F)	120.1
C(34F)-C(35F)-C(36F)	119.4(19)
C(34F)-C(35F)-H(35F)	120.3
C(36F)-C(35F)-H(35F)	120.3
C(31F)-C(36F)-C(35F)	122.6(19)
C(31F)-C(36F)-H(36F)	118.7
C(35F)-C(36F)-H(36F)	118.7
C(11A)-N(1A)-C(17A)	108.4(11)
C(11A)-N(1A)-C(15A)	108.1(11)
C(17A)-N(1A)-C(15A)	110.5(11)
C(11A)-N(1A)-C(13A)	110.6(12)
C(17A)-N(1A)-C(13A)	109.6(11)
C(15A)-N(1A)-C(13A)	109.6(11)
C(12A)-C(11A)-N(1A)	115.0(16)
C(12A)-C(11A)-H(11A)	108.5
N(1A)-C(11A)-H(11A)	108.5
C(12A)-C(11A)-H(11B)	108.5
N(1A)-C(11A)-H(11B)	108.5
H(11A)-C(11A)-H(11B)	107.5
C(11A)-C(12A)-H(12A)	109.5
C(11A)-C(12A)-H(12B)	109.5
H(12A)-C(12A)-H(12B)	109.5
C(11A)-C(12A)-H(12C)	109.5
H(12A)-C(12A)-H(12C)	109.5
H(12B)-C(12A)-H(12C)	109.5
C(14A)-C(13A)-N(1A)	115.7(16)
C(14A)-C(13A)-H(13A)	108.4
N(1A)-C(13A)-H(13A)	108.4
C(14A)-C(13A)-H(13B)	108.4
N(1A)-C(13A)-H(13B)	108.4
H(13A)-C(13A)-H(13B)	107.4
C(13A)-C(14A)-H(14A)	109.5
C(13A)-C(14A)-H(14B)	109.5
H(14A)-C(14A)-H(14B)	109.5
C(13A)-C(14A)-H(14C)	109.5
H(14A)-C(14A)-H(14C)	109.5
H(14B)-C(14A)-H(14C)	109.5
C(16A)-C(15A)-N(1A)	114.2(13)
C(16A)-C(15A)-H(15A)	108.7
N(1A)-C(15A)-H(15A)	108.7
C(16A)-C(15A)-H(15B)	108.7
N(1A)-C(15A)-H(15B)	108.7
H(15A)-C(15A)-H(15B)	107.6
C(15A)-C(16A)-H(16A)	109.5
C(15A)-C(16A)-H(16B)	109.5
H(16A)-C(16A)-H(16B)	109.5
C(15A)-C(16A)-H(16C)	109.5
H(16A)-C(16A)-H(16C)	109.5
H(16B)-C(16A)-H(16C)	109.5
N(1A)-C(17A)-C(18A)	117.2(12)
N(1A)-C(17A)-H(17A)	108.0
C(18A)-C(17A)-H(17A)	108.0
N(1A)-C(17A)-H(17B)	108.0
C(18A)-C(17A)-H(17B)	108.0
H(17A)-C(17A)-H(17B)	107.2
C(17A)-C(18A)-H(18A)	109.5
C(17A)-C(18A)-H(18B)	109.5
H(18A)-C(18A)-H(18B)	109.5
C(17A)-C(18A)-H(18C)	109.5
H(18A)-C(18A)-H(18C)	109.5
H(18B)-C(18A)-H(18C)	109.5
C(27A)-N(2A)-C(21A)	110.2(14)
C(27A)-N(2A)-C(25A)	112.8(15)
C(21A)-N(2A)-C(25A)	107.2(13)
C(27A)-N(2A)-C(23A)	106.5(13)
C(21A)-N(2A)-C(23A)	110.0(14)
C(25A)-N(2A)-C(23A)	110.2(14)
C(22A)-C(21A)-N(2A)	113.5(16)
C(22A)-C(21A)-H(21A)	108.9
N(2A)-C(21A)-H(21A)	108.9
C(22A)-C(21A)-H(21B)	108.9
N(2A)-C(21A)-H(21B)	108.9
H(21A)-C(21A)-H(21B)	107.7
C(21A)-C(22A)-H(22A)	109.5
C(21A)-C(22A)-H(22B)	109.5
H(22A)-C(22A)-H(22B)	109.5
C(21A)-C(22A)-H(22C)	109.5
H(22A)-C(22A)-H(22C)	109.5
H(22B)-C(22A)-H(22C)	109.5
C(24A)-C(23A)-N(2A)	115.1(14)

C(24A)-C(23A)-H(23A)	108.5
N(2A)-C(23A)-H(23A)	108.5
C(24A)-C(23A)-H(23B)	108.5
N(2A)-C(23A)-H(23B)	108.5
H(23A)-C(23A)-H(23B)	107.5
C(23A)-C(24A)-H(24A)	109.5
C(23A)-C(24A)-H(24B)	109.5
H(24A)-C(24A)-H(24B)	109.5
C(23A)-C(24A)-H(24C)	109.5
H(24A)-C(24A)-H(24C)	109.5
H(24B)-C(24A)-H(24C)	109.5
C(26A)-C(25A)-N(2A)	114.6(16)
C(26A)-C(25A)-H(25A)	108.6
N(2A)-C(25A)-H(25A)	108.6
C(26A)-C(25A)-H(25B)	108.6
N(2A)-C(25A)-H(25B)	108.6
H(25A)-C(25A)-H(25B)	107.6
C(25A)-C(26A)-H(26A)	109.5
C(25A)-C(26A)-H(26B)	109.5
H(26A)-C(26A)-H(26B)	109.5
C(25A)-C(26A)-H(26C)	109.5
H(26A)-C(26A)-H(26C)	109.5
H(26B)-C(26A)-H(26C)	109.5
N(2A)-C(27A)-C(28A)	115.6(14)
N(2A)-C(27A)-H(27A)	108.4
C(28A)-C(27A)-H(27A)	108.4
N(2A)-C(27A)-H(27B)	108.4
C(28A)-C(27A)-H(27B)	108.4
H(27A)-C(27A)-H(27B)	107.4
C(27A)-C(28A)-H(28A)	109.5
C(27A)-C(28A)-H(28B)	109.5
H(28A)-C(28A)-H(28B)	109.5
C(27A)-C(28A)-H(28C)	109.5
H(28A)-C(28A)-H(28C)	109.5
H(28B)-C(28A)-H(28C)	109.5
C(31A)-N(3A)-C(33A)	111.5(10)
C(31A)-N(3A)-C(35A)	105.0(9)
C(33A)-N(3A)-C(35A)	111.9(10)
C(31A)-N(3A)-C(37A)	111.7(11)
C(33A)-N(3A)-C(37A)	107.6(10)
C(35A)-N(3A)-C(37A)	109.1(10)
C(32A)-C(31A)-N(3A)	116.2(11)
C(32A)-C(31A)-H(31A)	108.2
N(3A)-C(31A)-H(31A)	108.2
C(32A)-C(31A)-H(31B)	108.2
N(3A)-C(31A)-H(31B)	108.2
H(31A)-C(31A)-H(31B)	107.4
C(31A)-C(32A)-H(32A)	109.5
C(31A)-C(32A)-H(32B)	109.5
H(32A)-C(32A)-H(32B)	109.5
C(31A)-C(32A)-H(32C)	109.5
H(32A)-C(32A)-H(32C)	109.5
H(32B)-C(32A)-H(32C)	109.5
C(34A)-C(33A)-N(3A)	115.8(11)
C(34A)-C(33A)-H(33A)	108.3
N(3A)-C(33A)-H(33A)	108.3
C(34A)-C(33A)-H(33B)	108.3
N(3A)-C(33A)-H(33B)	108.3
H(33A)-C(33A)-H(33B)	107.4
C(33A)-C(34A)-H(34A)	109.5
C(33A)-C(34A)-H(34B)	109.5
H(34A)-C(34A)-H(34B)	109.5
C(33A)-C(34A)-H(34C)	109.5
H(34A)-C(34A)-H(34C)	109.5
H(34B)-C(34A)-H(34C)	109.5
C(36A)-C(35A)-N(3A)	115.2(10)
C(36A)-C(35A)-H(35A)	108.5
N(3A)-C(35A)-H(35A)	108.5
C(36A)-C(35A)-H(35B)	108.5
N(3A)-C(35A)-H(35B)	108.5
H(35A)-C(35A)-H(35B)	107.5
C(35A)-C(36A)-H(36A)	109.5
C(35A)-C(36A)-H(36B)	109.5
H(36A)-C(36A)-H(36B)	109.5
C(35A)-C(36A)-H(36C)	109.5
H(36A)-C(36A)-H(36C)	109.5
H(36B)-C(36A)-H(36C)	109.5
C(38A)-C(37A)-N(3A)	117.2(12)
C(38A)-C(37A)-H(37A)	108.0
N(3A)-C(37A)-H(37A)	108.0
C(38A)-C(37A)-H(37B)	108.0
N(3A)-C(37A)-H(37B)	108.0
H(37A)-C(37A)-H(37B)	107.3
C(37A)-C(38A)-H(38A)	109.5
C(37A)-C(38A)-H(38B)	109.5
H(38A)-C(38A)-H(38B)	109.5
C(37A)-C(38A)-H(38C)	109.5
H(38A)-C(38A)-H(38C)	109.5
H(38B)-C(38A)-H(38C)	109.5
C(43A)-N(4A)-C(47A)	108(2)
C(43A)-N(4A)-C(45A)	108(2)
C(47A)-N(4A)-C(45A)	109(2)

C(43A)-N(4A)-C(41A)	115(2)	C(47A)-C(48A)-H(48B)	109.5
C(47A)-N(4A)-C(41A)	110(2)	H(48A)-C(48A)-H(48B)	109.5
C(45A)-N(4A)-C(41A)	106(2)	C(47A)-C(48A)-H(48C)	109.5
C(42A)-C(41A)-N(4A)	114(2)	H(48A)-C(48A)-H(48C)	109.5
C(42A)-C(41A)-H(41A)	108.7	H(48B)-C(48A)-H(48C)	109.5
N(4A)-C(41A)-H(41A)	108.7	C(13B)-N(1B)-C(17B)	109.5(19)
C(42A)-C(41A)-H(41B)	108.7	C(13B)-N(1B)-C(11B)	111(2)
N(4A)-C(41A)-H(41B)	108.7	C(17B)-N(1B)-C(11B)	110(2)
H(41A)-C(41A)-H(41B)	107.6	C(13B)-N(1B)-C(15B)	107.9(18)
C(41A)-C(42A)-H(42A)	109.5	C(17B)-N(1B)-C(15B)	110.7(19)
C(41A)-C(42A)-H(42B)	109.5	C(11B)-N(1B)-C(15B)	107(2)
H(42A)-C(42A)-H(42B)	109.5	C(12B)-C(11B)-N(1B)	116(3)
C(41A)-C(42A)-H(42C)	109.5	C(12B)-C(11B)-H(11C)	108.4
H(42A)-C(42A)-H(42C)	109.5	N(1B)-C(11B)-H(11C)	108.4
H(42B)-C(42A)-H(42C)	109.5	C(12B)-C(11B)-H(11D)	108.4
N(4A)-C(43A)-C(44A)	117(2)	N(1B)-C(11B)-H(11D)	108.4
N(4A)-C(43A)-H(43A)	108.1	H(11C)-C(11B)-H(11D)	107.4
C(44A)-C(43A)-H(43A)	108.1	C(11B)-C(12B)-H(12D)	109.5
N(4A)-C(43A)-H(43B)	108.1	C(11B)-C(12B)-H(12G)	109.5
C(44A)-C(43A)-H(43B)	108.1	H(12D)-C(12B)-H(12G)	109.5
H(43A)-C(43A)-H(43B)	107.3	C(11B)-C(12B)-H(12H)	109.5
C(43A)-C(44A)-H(44A)	109.5	H(12D)-C(12B)-H(12H)	109.5
C(43A)-C(44A)-H(44B)	109.5	H(12G)-C(12B)-H(12H)	109.5
H(44A)-C(44A)-H(44B)	109.5	C(14B)-C(13B)-N(1B)	117(3)
C(43A)-C(44A)-H(44C)	109.5	C(14B)-C(13B)-H(13C)	108.0
H(44A)-C(44A)-H(44C)	109.5	N(1B)-C(13B)-H(13C)	108.0
H(44B)-C(44A)-H(44C)	109.5	C(14B)-C(13B)-H(13D)	108.0
C(46A)-C(45A)-N(4A)	115(2)	N(1B)-C(13B)-H(13D)	108.0
C(46A)-C(45A)-H(45A)	108.5	H(13C)-C(13B)-H(13D)	107.2
N(4A)-C(45A)-H(45A)	108.5	C(13B)-C(14B)-H(14D)	109.5
C(46A)-C(45A)-H(45B)	108.5	C(13B)-C(14B)-H(14G)	109.5
N(4A)-C(45A)-H(45B)	108.5	H(14D)-C(14B)-H(14G)	109.5
H(45A)-C(45A)-H(45B)	107.5	C(13B)-C(14B)-H(14H)	109.5
C(45A)-C(46A)-H(46A)	109.5	H(14D)-C(14B)-H(14H)	109.5
C(45A)-C(46A)-H(46B)	109.5	H(14G)-C(14B)-H(14H)	109.5
H(46A)-C(46A)-H(46B)	109.5	C(16B)-C(15B)-N(1B)	115(2)
C(45A)-C(46A)-H(46C)	109.5	C(16B)-C(15B)-H(15C)	108.6
H(46A)-C(46A)-H(46C)	109.5	N(1B)-C(15B)-H(15C)	108.6
H(46B)-C(46A)-H(46C)	109.5	C(16B)-C(15B)-H(15D)	108.6
C(48A)-C(47A)-N(4A)	115(3)	N(1B)-C(15B)-H(15D)	108.6
C(48A)-C(47A)-H(47A)	108.5	H(15C)-C(15B)-H(15D)	107.6
N(4A)-C(47A)-H(47A)	108.5	C(15B)-C(16B)-H(16D)	109.5
C(48A)-C(47A)-H(47B)	108.5	C(15B)-C(16B)-H(16G)	109.5
N(4A)-C(47A)-H(47B)	108.5	H(16D)-C(16B)-H(16G)	109.5
H(47A)-C(47A)-H(47B)	107.5	C(15B)-C(16B)-H(16H)	109.5
C(47A)-C(48A)-H(48A)	109.5	H(16D)-C(16B)-H(16H)	109.5

H(16G)-C(16B)-H(16H)	109.5	C(26B)-C(25B)-H(25D)	107.8
C(18B)-C(17B)-N(1B)	117(2)	N(2B)-C(25B)-H(25D)	107.8
C(18B)-C(17B)-H(17C)	108.0	H(25C)-C(25B)-H(25D)	107.1
N(1B)-C(17B)-H(17C)	108.0	C(25B)-C(26B)-H(26D)	109.5
C(18B)-C(17B)-H(17D)	108.0	C(25B)-C(26B)-H(26G)	109.5
N(1B)-C(17B)-H(17D)	108.0	H(26D)-C(26B)-H(26G)	109.5
H(17C)-C(17B)-H(17D)	107.3	C(25B)-C(26B)-H(26H)	109.5
C(17B)-C(18B)-H(18D)	109.5	H(26D)-C(26B)-H(26H)	109.5
C(17B)-C(18B)-H(18E)	109.5	H(26G)-C(26B)-H(26H)	109.5
H(18D)-C(18B)-H(18E)	109.5	C(28B)-C(27B)-N(2B)	115.6(18)
C(17B)-C(18B)-H(18F)	109.5	C(28B)-C(27B)-H(27C)	108.4
H(18D)-C(18B)-H(18F)	109.5	N(2B)-C(27B)-H(27C)	108.4
H(18E)-C(18B)-H(18F)	109.5	C(28B)-C(27B)-H(27D)	108.4
C(25B)-N(2B)-C(21B)	105.2(16)	N(2B)-C(27B)-H(27D)	108.4
C(25B)-N(2B)-C(27B)	112.4(18)	H(27C)-C(27B)-H(27D)	107.4
C(21B)-N(2B)-C(27B)	113.1(18)	C(27B)-C(28B)-H(28D)	109.5
C(25B)-N(2B)-C(23B)	111.1(18)	C(27B)-C(28B)-H(28E)	109.5
C(21B)-N(2B)-C(23B)	111.0(17)	H(28D)-C(28B)-H(28E)	109.5
C(27B)-N(2B)-C(23B)	104.1(16)	C(27B)-C(28B)-H(28F)	109.5
C(22B)-C(21B)-N(2B)	115(2)	H(28D)-C(28B)-H(28F)	109.5
C(22B)-C(21B)-H(21C)	108.5	H(28E)-C(28B)-H(28F)	109.5
N(2B)-C(21B)-H(21C)	108.5	C(31B)-N(3B)-C(35B)	107(2)
C(22B)-C(21B)-H(21D)	108.5	C(31B)-N(3B)-C(33B)	110(2)
N(2B)-C(21B)-H(21D)	108.5	C(35B)-N(3B)-C(33B)	111(2)
H(21C)-C(21B)-H(21D)	107.5	C(31B)-N(3B)-C(37B)	111(2)
C(21B)-C(22B)-H(22D)	109.5	C(35B)-N(3B)-C(37B)	110(2)
C(21B)-C(22B)-H(22G)	109.5	C(33B)-N(3B)-C(37B)	107(2)
H(22D)-C(22B)-H(22G)	109.5	C(32B)-C(31B)-N(3B)	116(3)
C(21B)-C(22B)-H(22H)	109.5	C(32B)-C(31B)-H(31C)	108.4
H(22D)-C(22B)-H(22H)	109.5	N(3B)-C(31B)-H(31C)	108.4
H(22G)-C(22B)-H(22H)	109.5	C(32B)-C(31B)-H(31D)	108.4
C(24B)-C(23B)-N(2B)	115.1(18)	N(3B)-C(31B)-H(31D)	108.4
C(24B)-C(23B)-H(23C)	108.5	H(31C)-C(31B)-H(31D)	107.4
N(2B)-C(23B)-H(23C)	108.5	C(31B)-C(32B)-H(32D)	109.5
C(24B)-C(23B)-H(23D)	108.5	C(31B)-C(32B)-H(32G)	109.5
N(2B)-C(23B)-H(23D)	108.5	H(32D)-C(32B)-H(32G)	109.5
H(23C)-C(23B)-H(23D)	107.5	C(31B)-C(32B)-H(32H)	109.5
C(23B)-C(24B)-H(24D)	109.5	H(32D)-C(32B)-H(32H)	109.5
C(23B)-C(24B)-H(24G)	109.5	H(32G)-C(32B)-H(32H)	109.5
H(24D)-C(24B)-H(24G)	109.5	C(34B)-C(33B)-N(3B)	117(3)
C(23B)-C(24B)-H(24H)	109.5	C(34B)-C(33B)-H(33C)	108.1
H(24D)-C(24B)-H(24H)	109.5	N(3B)-C(33B)-H(33C)	108.1
H(24G)-C(24B)-H(24H)	109.5	C(34B)-C(33B)-H(33D)	108.1
C(26B)-C(25B)-N(2B)	118(2)	N(3B)-C(33B)-H(33D)	108.1
C(26B)-C(25B)-H(25C)	107.8	H(33C)-C(33B)-H(33D)	107.3
N(2B)-C(25B)-H(25C)	107.8	C(33B)-C(34B)-H(34D)	109.5

C(33B)-C(34B)-H(34G)	109.5	H(42E)-C(42B)-H(42F)	109.5
H(34D)-C(34B)-H(34G)	109.5	C(44B)-C(43B)-N(4B)	120(3)
C(33B)-C(34B)-H(34H)	109.5	C(44B)-C(43B)-H(43C)	107.3
H(34D)-C(34B)-H(34H)	109.5	N(4B)-C(43B)-H(43C)	107.3
H(34G)-C(34B)-H(34H)	109.5	C(44B)-C(43B)-H(43D)	107.3
C(36B)-C(35B)-N(3B)	114(3)	N(4B)-C(43B)-H(43D)	107.3
C(36B)-C(35B)-H(35C)	108.6	H(43C)-C(43B)-H(43D)	106.9
N(3B)-C(35B)-H(35C)	108.6	C(43B)-C(44B)-H(44D)	109.5
C(36B)-C(35B)-H(35D)	108.6	C(43B)-C(44B)-H(44E)	109.5
N(3B)-C(35B)-H(35D)	108.6	H(44D)-C(44B)-H(44E)	109.5
H(35C)-C(35B)-H(35D)	107.6	C(43B)-C(44B)-H(44F)	109.5
C(35B)-C(36B)-H(36D)	109.5	H(44D)-C(44B)-H(44F)	109.5
C(35B)-C(36B)-H(36G)	109.5	H(44E)-C(44B)-H(44F)	109.5
H(36D)-C(36B)-H(36G)	109.5	C(46B)-C(45B)-N(4B)	121(2)
C(35B)-C(36B)-H(36H)	109.5	C(46B)-C(45B)-H(45C)	107.0
H(36D)-C(36B)-H(36H)	109.5	N(4B)-C(45B)-H(45C)	107.0
H(36G)-C(36B)-H(36H)	109.5	C(46B)-C(45B)-H(45D)	107.0
C(38B)-C(37B)-N(3B)	116(3)	N(4B)-C(45B)-H(45D)	107.0
C(38B)-C(37B)-H(37C)	108.3	H(45C)-C(45B)-H(45D)	106.8
N(3B)-C(37B)-H(37C)	108.3	C(45B)-C(46B)-H(46D)	109.5
C(38B)-C(37B)-H(37D)	108.3	C(45B)-C(46B)-H(46E)	109.5
N(3B)-C(37B)-H(37D)	108.3	H(46D)-C(46B)-H(46E)	109.5
H(37C)-C(37B)-H(37D)	107.4	C(45B)-C(46B)-H(46F)	109.5
C(37B)-C(38B)-H(38D)	109.5	H(46D)-C(46B)-H(46F)	109.5
C(37B)-C(38B)-H(38E)	109.5	H(46E)-C(46B)-H(46F)	109.5
H(38D)-C(38B)-H(38E)	109.5	C(48B)-C(47B)-N(4B)	122(2)
C(37B)-C(38B)-H(38F)	109.5	C(48B)-C(47B)-H(47C)	106.8
H(38D)-C(38B)-H(38F)	109.5	N(4B)-C(47B)-H(47C)	106.8
H(38E)-C(38B)-H(38F)	109.5	C(48B)-C(47B)-H(47D)	106.8
C(43B)-N(4B)-C(47B)	111(2)	N(4B)-C(47B)-H(47D)	106.8
C(43B)-N(4B)-C(45B)	115.3(19)	H(47C)-C(47B)-H(47D)	106.6
C(47B)-N(4B)-C(45B)	114(2)	C(47B)-C(48B)-H(48D)	109.5
C(43B)-N(4B)-C(41B)	110(2)	C(47B)-C(48B)-H(48E)	109.5
C(47B)-N(4B)-C(41B)	107(2)	H(48D)-C(48B)-H(48E)	109.5
C(45B)-N(4B)-C(41B)	99(2)	C(47B)-C(48B)-H(48F)	109.5
C(42B)-C(41B)-N(4B)	110(2)	H(48D)-C(48B)-H(48F)	109.5
C(42B)-C(41B)-H(41C)	109.7	H(48E)-C(48B)-H(48F)	109.5
N(4B)-C(41B)-H(41C)	109.7	Cl(1S)-C(1S)-Cl(2S)	123.6(18)
C(42B)-C(41B)-H(41D)	109.7	Cl(1S)-C(1S)-H(1S1)	106.4
N(4B)-C(41B)-H(41D)	109.7	Cl(2S)-C(1S)-H(1S1)	106.4
H(41C)-C(41B)-H(41D)	108.2	Cl(1S)-C(1S)-H(1S2)	106.4
C(41B)-C(42B)-H(42D)	109.5	Cl(2S)-C(1S)-H(1S2)	106.4
C(41B)-C(42B)-H(42E)	109.5	H(1S1)-C(1S)-H(1S2)	106.5
H(42D)-C(42B)-H(42E)	109.5	Cl(3T)-C(2T)-Cl(4T)	110.6(19)
C(41B)-C(42B)-H(42F)	109.5	Cl(3T)-C(2T)-H(2T1)	109.5
H(42D)-C(42B)-H(42F)	109.5	Cl(4T)-C(2T)-H(2T1)	109.5

Cl(3T)-C(2T)-H(2T2)	109.5	H(2S1)-C(2S)-H(2S2)	107.7
Cl(4T)-C(2T)-H(2T2)	109.5	Cl(5S)-C(3S)-Cl(6S)	112.1(17)
H(2T1)-C(2T)-H(2T2)	108.1	Cl(5S)-C(3S)-H(3S1)	109.2
Cl(4U)-C(2U)-Cl(3U)	111(2)	Cl(6S)-C(3S)-H(3S1)	109.2
Cl(4U)-C(2U)-H(2U1)	109.5	Cl(5S)-C(3S)-H(3S2)	109.2
Cl(3U)-C(2U)-H(2U1)	109.5	Cl(6S)-C(3S)-H(3S2)	109.2
Cl(4U)-C(2U)-H(2U2)	109.5	H(3S1)-C(3S)-H(3S2)	107.9
Cl(3U)-C(2U)-H(2U2)	109.5	Cl(5T)-C(3T)-Cl(6T)	111(2)
H(2U1)-C(2U)-H(2U2)	108.0	Cl(5T)-C(3T)-H(3T1)	109.4
Cl(3S)-C(2S)-Cl(4S)	113.2(16)	Cl(6T)-C(3T)-H(3T1)	109.4
Cl(3S)-C(2S)-H(2S1)	108.9	Cl(5T)-C(3T)-H(3T2)	109.4
Cl(4S)-C(2S)-H(2S1)	108.9	Cl(6T)-C(3T)-H(3T2)	109.4
Cl(3S)-C(2S)-H(2S2)	108.9	H(3T1)-C(3T)-H(3T2)	108.0
Cl(4S)-C(2S)-H(2S2)	108.9		

c) Complex 3:

Collection and Refinement of complex 3:

A crystal was mounted on a polyimide MiTeGen loop with STP Oil Treatment and placed under a nitrogen stream. Low temperature (100K) X-ray data were obtained on a Bruker AXS D8 VENTURE KAPPA diffractometer with an IμS micro-source (50 kV and 1mA; Cu K_{α} radiation, λ = 1.54178 Å), Helios focusing multilayer mirror optics, and a PHOTON II CPAD detector. All diffractometer manipulations, including data collection, integration, and scaling were carried out using the Bruker APEX3 software. An absorption correction was applied using SADABS. The space group was determined on the basis of systematic absences and intensity statistics; the structure was solved by intrinsic phasing using XT and refined by full-matrix least squares on F^2 using XL. All non-hydrogen atoms were refined using anisotropic displacement parameters. Hydrogen atoms were placed in idealized positions and refined using a riding model. The isotropic displacement parameters of all hydrogen atoms were fixed at 1.2 times the U_{eq} value of the bonded atom.

$(PPN)_4[Fe(CN\text{-}B(C_6F_5)_3)_6]$ (**3**) crystallizes in the triclinic space group *P*-1 with half an anion (the Fe lies on a center of symmetry), two cations, and some CH_2Cl_2 in the asymmetric unit. Collecting data at temperatures above 100 K did not improve the structure. A large solvent region consists of disordered CH_2Cl_2 molecules. A few are modelled with restraints. Although difference peaks at the characteristic 2.9 Å separation indicated additional CH_2Cl_2, these would not behave during refinement and therefore an OLEX2 solvent mask was employed (617.8 Å^3; 242.8 e$^-$).

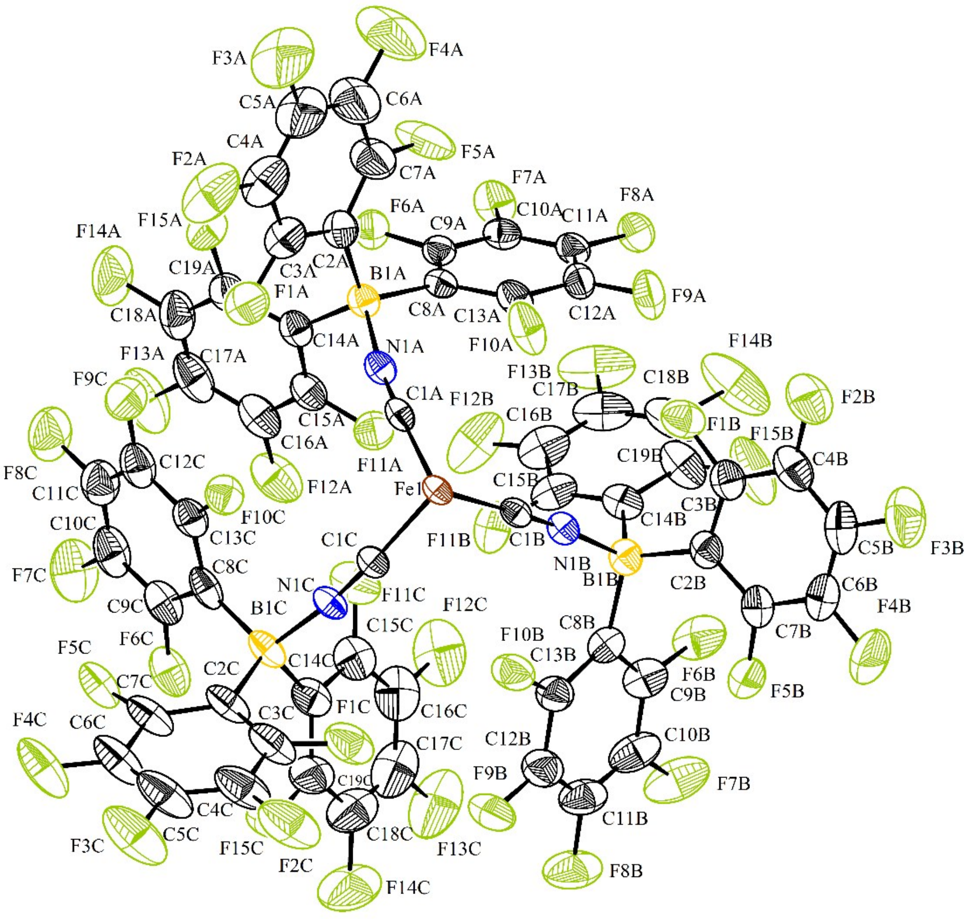

Figure S13. Crystal structure of **3** (protons and cations are omitted for clarity).

Table S6. Crystal data and structure refinement for complex **3**. Thermal ellipsoids set at 50% probability.

Empirical formula	C263.25 H130.49 B6 Cl10.49 F90 Fe N10 P8	
Moiety formula	C114 B6 F90 Fe N6, 4(C36 H30 N P2), 5.25(C H2 Cl2)	
Formula weight	5883.60	
Temperature	100 K	
Wavelength	1.54178 Å	
Crystal system	Triclinic	
Space group	P-1	
Unit cell dimensions	a = 19.290(4) Å	α= 65.073(6)°
	b = 19.912(4) Å	β= 68.449(19)°
	c = 20.432(4) Å	γ = 80.849(6)°
Volume	6619(2) Å^3	
Z	1	

Density (calculated)	1.476 g/cm^3
Absorption coefficient	2.947 mm^{-1}
F(000)	2944
Crystal size	0.21 x 0.11 x 0.08 mm^3
Theta range for data collection	2.447 to 70.367°.
Index ranges	-22 ≤ h ≤ 23, -23 ≤ k ≤ 24, -24 ≤ l ≤ 24
Reflections collected	158484
Independent reflections	24649 [R(int) = 0.0483]
Completeness to theta = 67.679°	99.1 %
Absorption correction	Semi-empirical from equivalents
Max. and min. transmission	1.0000 and 0.7413
Refinement method	Full-matrix least-squares on F^2
Data / restraints / parameters	24649 / 48 / 1839
Goodness-of-fit on F^2	1.058
Final R indices [I>2sigma(I)]	R1 = 0.0743, wR2 = 0.2117
R indices (all data)	R1 = 0.0915, wR2 = 0.2272
Extinction coefficient	n/a
Largest diff. peak and hole	1.628 and -0.999 e.Å^{-3}

Table S7. Bond lengths [Å] and angles [°] for **3** (Symmetry transformations used to generate equivalent atoms: #1 -x+1,-y+1,-z+1).

Fe(1)-C(1A)#1	1.899(4)
Fe(1)-C(1A)	1.899(4)
Fe(1)-C(1B)	1.904(3)
Fe(1)-C(1B)#1	1.904(3)
Fe(1)-C(1C)	1.897(3)
Fe(1)-C(1C)#1	1.897(3)
F(1A)-C(3A)	1.340(5)
F(2A)-C(4A)	1.352(6)
F(3A)-C(5A)	1.360(6)
F(4A)-C(6A)	1.354(7)
F(5A)-C(7A)	1.344(6)
F(6A)-C(9A)	1.361(4)
F(7A)-C(10A)	1.348(4)
F(8A)-C(11A)	1.349(4)
F(9A)-C(12A)	1.347(4)
F(10A)-C(13A)	1.347(4)
F(11A)-C(15A)	1.342(5)
F(12A)-C(16A)	1.348(5)
F(13A)-C(17A)	1.350(5)
F(14A)-C(18A)	1.351(5)
F(15A)-C(19A)	1.352(5)
F(1B)-C(3B)	1.343(5)
F(2B)-C(4B)	1.344(5)
F(3B)-C(5B)	1.349(5)
F(4B)-C(6B)	1.359(6)
F(5B)-C(7B)	1.361(5)
F(6B)-C(9B)	1.352(5)
F(7B)-C(10B)	1.358(5)
F(8B)-C(11B)	1.348(5)
F(9B)-C(12B)	1.348(6)
F(10B)-C(13B)	1.348(5)
F(11B)-C(15B)	1.335(5)
F(12B)-C(16B)	1.345(6)
F(13B)-C(17B)	1.354(5)

F(14B)-C(18B)	1.356(6)
F(15B)-C(19B)	1.331(6)
F(1C)-C(3C)	1.365(6)
F(2C)-C(4C)	1.344(6)
F(3C)-C(5C)	1.352(5)
F(4C)-C(6C)	1.345(6)
F(5C)-C(7C)	1.339(5)
F(6C)-C(9C)	1.356(5)
F(7C)-C(10C)	1.352(5)
F(8C)-C(11C)	1.344(5)
F(9C)-C(12C)	1.331(5)
F(10C)-C(13C)	1.338(4)
F(11C)-C(15C)	1.327(5)
F(12C)-C(16C)	1.338(7)
F(13C)-C(17C)	1.366(6)
F(14C)-C(18C)	1.367(7)
F(15C)-C(19C)	1.362(6)
N(1A)-C(1A)	1.146(4)
N(1A)-B(1A)	1.545(5)
N(1B)-C(1B)	1.152(4)
N(1B)-B(1B)	1.550(5)
N(1C)-C(1C)	1.160(4)
N(1C)-B(1C)	1.551(4)
C(2A)-C(3A)	1.384(6)
C(2A)-C(7A)	1.371(6)
C(2A)-B(1A)	1.632(6)
C(3A)-C(4A)	1.379(7)
C(4A)-C(5A)	1.366(9)
C(5A)-C(6A)	1.341(9)
C(6A)-C(7A)	1.389(7)
C(8A)-C(9A)	1.382(5)
C(8A)-C(13A)	1.397(5)
C(8A)-B(1A)	1.648(5)
C(9A)-C(10A)	1.380(5)
C(10A)-C(11A)	1.370(5)
C(11A)-C(12A)	1.363(5)
C(12A)-C(13A)	1.375(5)
C(14A)-C(15A)	1.381(6)
C(14A)-C(19A)	1.392(5)
C(14A)-B(1A)	1.654(5)
C(15A)-C(16A)	1.383(6)
C(16A)-C(17A)	1.367(7)
C(17A)-C(18A)	1.359(8)
C(18A)-C(19A)	1.390(6)
C(2B)-C(3B)	1.391(6)
C(2B)-C(7B)	1.374(6)
C(2B)-B(1B)	1.651(6)
C(3B)-C(4B)	1.384(6)
C(4B)-C(5B)	1.361(7)
C(5B)-C(6B)	1.361(7)
C(6B)-C(7B)	1.367(7)
C(8B)-C(9B)	1.394(5)
C(8B)-C(13B)	1.382(6)
C(8B)-B(1B)	1.645(6)
C(9B)-C(10B)	1.382(6)
C(10B)-C(11B)	1.355(8)
C(11B)-C(12B)	1.371(7)
C(12B)-C(13B)	1.387(6)
C(14B)-C(15B)	1.380(6)
C(14B)-C(19B)	1.382(6)
C(14B)-B(1B)	1.666(5)
C(15B)-C(16B)	1.400(6)
C(16B)-C(17B)	1.330(8)
C(17B)-C(18B)	1.367(8)
C(18B)-C(19B)	1.414(7)
C(2C)-C(3C)	1.374(6)
C(2C)-C(7C)	1.395(6)
C(2C)-B(1C)	1.639(5)
C(3C)-C(4C)	1.385(7)
C(4C)-C(5C)	1.382(9)
C(5C)-C(6C)	1.352(8)
C(6C)-C(7C)	1.386(6)
C(8C)-C(9C)	1.392(5)
C(8C)-C(13C)	1.379(6)
C(8C)-B(1C)	1.645(6)
C(9C)-C(10C)	1.393(7)
C(10C)-C(11C)	1.370(7)
C(11C)-C(12C)	1.349(7)
C(12C)-C(13C)	1.410(6)
C(14C)-C(15C)	1.379(6)
C(14C)-C(19C)	1.375(6)
C(14C)-B(1C)	1.655(6)
C(15C)-C(16C)	1.389(6)
C(16C)-C(17C)	1.345(9)
C(17C)-C(18C)	1.349(10)
C(18C)-C(19C)	1.383(8)
P(1B)-N(2B)	1.582(4)
P(1B)-C(20B)	1.792(4)
P(1B)-C(26B)	1.803(5)
P(1B)-C(32B)	1.800(4)
P(2B)-N(2B)	1.580(4)
P(2B)-C(38B)	1.805(5)

P(2B)-C(44B)	1.802(4)
P(2B)-C(50B)	1.795(5)
C(20B)-C(21B)	1.388(6)
C(20B)-C(25B)	1.388(6)
C(21B)-H(21B)	0.9500
C(21B)-C(22B)	1.383(6)
C(22B)-H(22B)	0.9500
C(22B)-C(23B)	1.363(7)
C(23B)-H(23B)	0.9500
C(23B)-C(24B)	1.365(8)
C(24B)-H(24B)	0.9500
C(24B)-C(25B)	1.378(7)
C(25B)-H(25B)	0.9500
C(26B)-C(27B)	1.395(7)
C(26B)-C(31B)	1.390(7)
C(27B)-H(27B)	0.9500
C(27B)-C(28B)	1.370(8)
C(28B)-H(28B)	0.9500
C(28B)-C(29B)	1.367(10)
C(29B)-H(29B)	0.9500
C(29B)-C(30B)	1.367(11)
C(30B)-H(30B)	0.9500
C(30B)-C(31B)	1.390(8)
C(31B)-H(31B)	0.9500
C(32B)-C(33B)	1.393(6)
C(32B)-C(37B)	1.396(5)
C(33B)-H(33B)	0.9500
C(33B)-C(34B)	1.380(6)
C(34B)-H(34B)	0.9500
C(34B)-C(35B)	1.371(6)
C(35B)-H(35B)	0.9500
C(35B)-C(36B)	1.376(6)
C(36B)-H(36B)	0.9500
C(36B)-C(37B)	1.383(6)
C(37B)-H(37B)	0.9500
C(38B)-C(39B)	1.369(7)
C(38B)-C(43B)	1.373(7)
C(39B)-H(39B)	0.9500
C(39B)-C(40B)	1.371(9)
C(40B)-H(40B)	0.9500
C(40B)-C(41B)	1.376(10)
C(41B)-H(41B)	0.9500
C(41B)-C(42B)	1.351(9)
C(42B)-H(42B)	0.9500
C(42B)-C(43B)	1.373(8)
C(43B)-H(43B)	0.9500
C(44B)-C(45B)	1.391(7)
C(44B)-C(49B)	1.358(7)
C(45B)-H(45B)	0.9500
C(45B)-C(46B)	1.390(6)
C(46B)-H(46B)	0.9500
C(46B)-C(47B)	1.327(7)
C(47B)-H(47B)	0.9500
C(47B)-C(48B)	1.355(8)
C(48B)-H(48B)	0.9500
C(48B)-C(49B)	1.392(8)
C(49B)-H(49B)	0.9500
C(50B)-C(51B)	1.297(9)
C(50B)-C(55B)	1.415(9)
C(50B)-C(51C)	1.45(2)
C(50B)-C(55C)	1.391(17)
C(53B)-H(53B)	0.9500
C(53B)-H(53A)	0.9500
C(53B)-C(52B)	1.317(12)
C(53B)-C(54B)	1.464(10)
C(53B)-C(52C)	1.38(3)
C(53B)-C(54C)	1.320(18)
C(51B)-H(51B)	0.9500
C(51B)-C(52B)	1.392(11)
C(52B)-H(52B)	0.9500
C(54B)-H(54B)	0.9500
C(54B)-C(55B)	1.384(10)
C(55B)-H(55B)	0.9500
C(51C)-H(51C)	0.9500
C(51C)-C(52C)	1.42(3)
C(52C)-H(52C)	0.9500
C(54C)-H(54C)	0.9500
C(54C)-C(55C)	1.40(2)
C(55C)-H(55C)	0.9500
P(1A)-N(2A)	1.577(3)
P(1A)-C(20A)	1.800(4)
P(1A)-C(26A)	1.794(4)
P(1A)-C(32A)	1.808(5)
P(2A)-N(2A)	1.572(3)
P(2A)-C(38A)	1.797(4)
P(2A)-C(44A)	1.807(4)
P(2A)-C(50A)	1.799(4)
C(20A)-C(21A)	1.379(7)
C(20A)-C(25A)	1.395(7)
C(21A)-H(21A)	0.9500
C(21A)-C(22A)	1.397(6)
C(22A)-H(22A)	0.9500

C(22A)-C(23A)	1.369(8)
C(23A)-H(23A)	0.9500
C(23A)-C(24A)	1.361(10)
C(24A)-H(24A)	0.9500
C(24A)-C(25A)	1.392(8)
C(25A)-H(25A)	0.9500
C(26A)-C(27A)	1.397(6)
C(26A)-C(31A)	1.399(6)
C(27A)-H(27A)	0.9500
C(27A)-C(28A)	1.383(7)
C(28A)-H(28A)	0.9500
C(28A)-C(29A)	1.383(8)
C(29A)-H(29A)	0.9500
C(29A)-C(30A)	1.386(7)
C(30A)-H(30A)	0.9500
C(30A)-C(31A)	1.376(6)
C(31A)-H(31A)	0.9500
C(32A)-C(33A)	1.377(8)
C(32A)-C(37A)	1.393(7)
C(33A)-H(33A)	0.9500
C(33A)-C(34A)	1.406(10)
C(34A)-H(34A)	0.9500
C(34A)-C(35A)	1.380(13)
C(35A)-H(35A)	0.9500
C(35A)-C(36A)	1.350(12)
C(36A)-H(36A)	0.9500
C(36A)-C(37A)	1.385(7)
C(37A)-H(37A)	0.9500
C(38A)-C(39A)	1.388(5)
C(38A)-C(43A)	1.384(5)
C(39A)-H(39A)	0.9500
C(39A)-C(40A)	1.388(6)
C(40A)-H(40A)	0.9500
C(40A)-C(41A)	1.374(6)
C(41A)-H(41A)	0.9500
C(41A)-C(42A)	1.373(6)
C(42A)-H(42A)	0.9500
C(42A)-C(43A)	1.393(6)
C(43A)-H(43A)	0.9500
C(44A)-C(45A)	1.385(6)
C(44A)-C(49A)	1.405(6)
C(45A)-H(45A)	0.9500
C(45A)-C(46A)	1.400(6)
C(46A)-H(46A)	0.9500
C(46A)-C(47A)	1.371(8)
C(47A)-H(47A)	0.9500
C(47A)-C(48A)	1.373(8)
C(48A)-H(48A)	0.9500
C(48A)-C(49A)	1.369(6)
C(49A)-H(49A)	0.9500
C(50A)-C(51A)	1.397(5)
C(50A)-C(55A)	1.387(5)
C(51A)-H(51A)	0.9500
C(51A)-C(52A)	1.379(6)
C(52A)-H(52A)	0.9500
C(52A)-C(53A)	1.376(6)
C(53A)-H(53C)	0.9500
C(53A)-C(54A)	1.370(7)
C(54A)-H(54A)	0.9500
C(54A)-C(55A)	1.399(6)
C(55A)-H(55A)	0.9500
Cl(1)-C(56)	1.697(8)
Cl(2A)-C(56)	1.748(10)
Cl(2B)-C(56)	1.748(12)
C(56)-H(56C)	0.9900
C(56)-H(56D)	0.9900
C(56)-H(56A)	0.9900
C(56)-H(56B)	0.9900
Cl(3A)-C(57A)	1.755(12)
Cl(4A)-C(57A)	1.744(9)
C(57A)-H(57A)	0.9900
C(57A)-H(57B)	0.9900
Cl(5)-C(58)	1.65(2)
Cl(6)-C(58)	1.794(18)
C(58)-H(58A)	0.9900
C(58)-H(58B)	0.9900
Cl(3B)-C(57B)	1.754(16)
Cl(4B)-C(57B)	1.723(16)
C(57B)-H(57C)	0.9900
C(57B)-H(57D)	0.9900
C(1A)#1-Fe(1)-C(1A)	180.0
C(1A)-Fe(1)-C(1B)	89.60(13)
C(1A)#1-Fe(1)-C(1B)	90.40(13)
C(1A)#1-Fe(1)-C(1B)#1	89.60(13)
C(1A)-Fe(1)-C(1B)#1	90.40(13)
C(1B)#1-Fe(1)-C(1B)	180.0
C(1C)#1-Fe(1)-C(1A)#1	89.30(13)
C(1C)#1-Fe(1)-C(1A)	90.70(13)
C(1C)-Fe(1)-C(1A)	89.30(13)
C(1C)-Fe(1)-C(1A)#1	90.70(13)
C(1C)#1-Fe(1)-C(1B)	89.40(13)

C(1C)-Fe(1)-C(1B)#1	89.40(13)	C(19A)-C(14A)-B(1A)	127.0(3)
C(1C)#1-Fe(1)-C(1B)#1	90.60(13)	F(11A)-C(15A)-C(14A)	119.0(3)
C(1C)-Fe(1)-C(1B)	90.60(13)	F(11A)-C(15A)-C(16A)	116.8(4)
C(1C)#1-Fe(1)-C(1C)	180.0	C(14A)-C(15A)-C(16A)	124.2(4)
C(1A)-N(1A)-B(1A)	173.2(3)	F(12A)-C(16A)-C(15A)	120.6(4)
C(1B)-N(1B)-B(1B)	175.1(3)	F(12A)-C(16A)-C(17A)	120.2(4)
C(1C)-N(1C)-B(1C)	173.3(4)	C(17A)-C(16A)-C(15A)	119.2(4)
N(1A)-C(1A)-Fe(1)	176.9(3)	F(13A)-C(17A)-C(16A)	119.5(5)
C(3A)-C(2A)-B(1A)	120.5(3)	F(13A)-C(17A)-C(18A)	120.7(5)
C(7A)-C(2A)-C(3A)	113.7(4)	C(18A)-C(17A)-C(16A)	119.7(4)
C(7A)-C(2A)-B(1A)	125.8(3)	F(14A)-C(18A)-C(17A)	120.8(4)
F(1A)-C(3A)-C(2A)	119.6(4)	F(14A)-C(18A)-C(19A)	119.5(5)
F(1A)-C(3A)-C(4A)	116.8(4)	C(17A)-C(18A)-C(19A)	119.6(4)
C(4A)-C(3A)-C(2A)	123.5(4)	F(15A)-C(19A)-C(14A)	120.9(4)
F(2A)-C(4A)-C(3A)	120.7(5)	F(15A)-C(19A)-C(18A)	115.8(4)
F(2A)-C(4A)-C(5A)	119.6(5)	C(18A)-C(19A)-C(14A)	123.3(4)
C(5A)-C(4A)-C(3A)	119.7(5)	N(1B)-C(1B)-Fe(1)	177.9(3)
F(3A)-C(5A)-C(4A)	119.1(6)	C(3B)-C(2B)-B(1B)	119.5(3)
C(6A)-C(5A)-F(3A)	121.5(6)	C(7B)-C(2B)-C(3B)	113.1(4)
C(6A)-C(5A)-C(4A)	119.3(5)	C(7B)-C(2B)-B(1B)	127.4(4)
F(4A)-C(6A)-C(7A)	120.0(6)	F(1B)-C(3B)-C(2B)	119.6(3)
C(5A)-C(6A)-F(4A)	120.2(5)	F(1B)-C(3B)-C(4B)	116.7(4)
C(5A)-C(6A)-C(7A)	119.8(5)	C(4B)-C(3B)-C(2B)	123.8(4)
F(5A)-C(7A)-C(2A)	121.1(4)	F(2B)-C(4B)-C(3B)	119.8(4)
F(5A)-C(7A)-C(6A)	115.0(5)	F(2B)-C(4B)-C(5B)	120.6(4)
C(2A)-C(7A)-C(6A)	123.9(5)	C(5B)-C(4B)-C(3B)	119.6(4)
C(9A)-C(8A)-C(13A)	112.8(3)	F(3B)-C(5B)-C(4B)	120.3(5)
C(9A)-C(8A)-B(1A)	122.6(3)	F(3B)-C(5B)-C(6B)	120.9(5)
C(13A)-C(8A)-B(1A)	124.6(3)	C(6B)-C(5B)-C(4B)	118.8(4)
F(6A)-C(9A)-C(8A)	120.2(3)	F(4B)-C(6B)-C(5B)	119.0(4)
F(6A)-C(9A)-C(10A)	114.9(3)	F(4B)-C(6B)-C(7B)	121.0(5)
C(10A)-C(9A)-C(8A)	124.8(3)	C(5B)-C(6B)-C(7B)	120.0(5)
F(7A)-C(10A)-C(9A)	120.7(3)	F(5B)-C(7B)-C(2B)	120.8(4)
F(7A)-C(10A)-C(11A)	120.1(3)	F(5B)-C(7B)-C(6B)	114.6(4)
C(11A)-C(10A)-C(9A)	119.2(3)	C(6B)-C(7B)-C(2B)	124.7(4)
F(8A)-C(11A)-C(10A)	120.9(3)	C(9B)-C(8B)-B(1B)	119.8(3)
F(8A)-C(11A)-C(12A)	120.0(3)	C(13B)-C(8B)-C(9B)	112.8(4)
C(12A)-C(11A)-C(10A)	119.1(3)	C(13B)-C(8B)-B(1B)	127.4(3)
F(9A)-C(12A)-C(11A)	118.7(3)	F(6B)-C(9B)-C(8B)	119.0(4)
F(9A)-C(12A)-C(13A)	121.4(3)	F(6B)-C(9B)-C(10B)	116.7(4)
C(11A)-C(12A)-C(13A)	119.9(3)	C(10B)-C(9B)-C(8B)	124.3(4)
F(10A)-C(13A)-C(8A)	120.8(3)	F(7B)-C(10B)-C(9B)	119.9(5)
F(10A)-C(13A)-C(12A)	115.1(3)	C(11B)-C(10B)-F(7B)	120.1(4)
C(12A)-C(13A)-C(8A)	124.1(3)	C(11B)-C(10B)-C(9B)	120.0(4)
C(15A)-C(14A)-C(19A)	113.9(3)	F(8B)-C(11B)-C(10B)	120.8(5)
C(15A)-C(14A)-B(1A)	118.9(3)	F(8B)-C(11B)-C(12B)	120.3(5)

C(10B)-C(11B)-C(12B)	118.9(4)
F(9B)-C(12B)-C(11B)	119.8(4)
F(9B)-C(12B)-C(13B)	120.5(4)
C(11B)-C(12B)-C(13B)	119.6(4)
F(10B)-C(13B)-C(8B)	121.3(3)
F(10B)-C(13B)-C(12B)	114.2(4)
C(8B)-C(13B)-C(12B)	124.4(4)
C(15B)-C(14B)-C(19B)	114.1(4)
C(15B)-C(14B)-B(1B)	118.9(3)
C(19B)-C(14B)-B(1B)	126.9(4)
F(11B)-C(15B)-C(14B)	119.3(3)
F(11B)-C(15B)-C(16B)	116.1(4)
C(14B)-C(15B)-C(16B)	124.6(4)
F(12B)-C(16B)-C(15B)	120.9(5)
C(17B)-C(16B)-F(12B)	120.0(4)
C(17B)-C(16B)-C(15B)	119.0(5)
F(13B)-C(17B)-C(18B)	118.1(5)
C(16B)-C(17B)-F(13B)	121.6(6)
C(16B)-C(17B)-C(18B)	120.2(4)
F(14B)-C(18B)-C(17B)	121.8(5)
F(14B)-C(18B)-C(19B)	118.3(6)
C(17B)-C(18B)-C(19B)	119.9(4)
F(15B)-C(19B)-C(14B)	121.4(4)
F(15B)-C(19B)-C(18B)	116.5(4)
C(14B)-C(19B)-C(18B)	122.1(5)
N(1C)-C(1C)-Fe(1)	175.9(3)
C(3C)-C(2C)-C(7C)	114.1(4)
C(3C)-C(2C)-B(1C)	120.6(4)
C(7C)-C(2C)-B(1C)	125.3(4)
F(1C)-C(3C)-C(2C)	119.4(4)
F(1C)-C(3C)-C(4C)	117.2(4)
C(2C)-C(3C)-C(4C)	123.3(5)
F(2C)-C(4C)-C(3C)	120.3(5)
F(2C)-C(4C)-C(5C)	119.8(4)
C(5C)-C(4C)-C(3C)	120.0(5)
F(3C)-C(5C)-C(4C)	120.9(5)
F(3C)-C(5C)-C(6C)	120.1(5)
C(6C)-C(5C)-C(4C)	119.1(4)
F(4C)-C(6C)-C(5C)	120.0(4)
F(4C)-C(6C)-C(7C)	120.4(5)
C(5C)-C(6C)-C(7C)	119.6(5)
F(5C)-C(7C)-C(2C)	121.6(3)
F(5C)-C(7C)-C(6C)	114.7(4)
C(6C)-C(7C)-C(2C)	123.8(4)
C(9C)-C(8C)-B(1C)	123.0(4)
C(13C)-C(8C)-C(9C)	112.9(4)

C(13C)-C(8C)-B(1C)	124.1(3)
F(6C)-C(9C)-C(8C)	119.1(4)
F(6C)-C(9C)-C(10C)	116.9(4)
C(8C)-C(9C)-C(10C)	123.9(4)
F(7C)-C(10C)-C(9C)	119.6(5)
F(7C)-C(10C)-C(11C)	120.6(4)
C(11C)-C(10C)-C(9C)	119.9(4)
F(8C)-C(11C)-C(10C)	119.5(4)
F(8C)-C(11C)-C(12C)	121.0(5)
C(12C)-C(11C)-C(10C)	119.5(4)
F(9C)-C(12C)-C(11C)	120.1(4)
F(9C)-C(12C)-C(13C)	120.9(4)
C(11C)-C(12C)-C(13C)	119.0(5)
F(10C)-C(13C)-C(8C)	121.4(3)
F(10C)-C(13C)-C(12C)	113.8(4)
C(8C)-C(13C)-C(12C)	124.8(4)
C(15C)-C(14C)-B(1C)	118.8(4)
C(19C)-C(14C)-C(15C)	114.0(4)
C(19C)-C(14C)-B(1C)	127.1(4)
F(11C)-C(15C)-C(14C)	119.3(4)
F(11C)-C(15C)-C(16C)	117.3(4)
C(14C)-C(15C)-C(16C)	123.5(5)
F(12C)-C(16C)-C(15C)	121.0(5)
F(12C)-C(16C)-C(17C)	119.2(5)
C(17C)-C(16C)-C(15C)	119.7(6)
C(16C)-C(17C)-F(13C)	119.7(7)
C(16C)-C(17C)-C(18C)	119.1(5)
C(18C)-C(17C)-F(13C)	121.1(6)
F(14C)-C(18C)-C(19C)	118.6(7)
C(17C)-C(18C)-F(14C)	120.8(6)
C(17C)-C(18C)-C(19C)	120.6(5)
F(15C)-C(19C)-C(14C)	120.7(4)
F(15C)-C(19C)-C(18C)	116.3(5)
C(14C)-C(19C)-C(18C)	123.0(5)
N(1A)-B(1A)-C(2A)	105.9(3)
N(1A)-B(1A)-C(8A)	108.0(3)
N(1A)-B(1A)-C(14A)	107.0(3)
C(2A)-B(1A)-C(8A)	112.3(3)
C(2A)-B(1A)-C(14A)	114.7(3)
C(8A)-B(1A)-C(14A)	108.6(3)
N(1B)-B(1B)-C(2B)	105.9(3)
N(1B)-B(1B)-C(8B)	108.9(3)
N(1B)-B(1B)-C(14B)	106.9(3)
C(2B)-B(1B)-C(14B)	113.1(3)
C(8B)-B(1B)-C(2B)	112.9(3)
C(8B)-B(1B)-C(14B)	108.9(3)

N(1C)-B(1C)-C(2C)	107.1(3)	C(28B)-C(29B)-H(29B)	119.1
N(1C)-B(1C)-C(8C)	107.5(3)	C(28B)-C(29B)-C(30B)	121.9(6)
N(1C)-B(1C)-C(14C)	105.0(3)	C(30B)-C(29B)-H(29B)	119.1
C(2C)-B(1C)-C(8C)	112.2(3)	C(29B)-C(30B)-H(30B)	120.4
C(2C)-B(1C)-C(14C)	112.8(3)	C(29B)-C(30B)-C(31B)	119.2(7)
C(8C)-B(1C)-C(14C)	111.8(3)	C(31B)-C(30B)-H(30B)	120.4
N(2B)-P(1B)-C(20B)	109.62(19)	C(26B)-C(31B)-C(30B)	119.9(6)
N(2B)-P(1B)-C(26B)	109.1(2)	C(26B)-C(31B)-H(31B)	120.0
N(2B)-P(1B)-C(32B)	114.52(18)	C(30B)-C(31B)-H(31B)	120.0
C(20B)-P(1B)-C(26B)	106.7(2)	C(33B)-C(32B)-P(1B)	120.2(3)
C(20B)-P(1B)-C(32B)	108.13(19)	C(33B)-C(32B)-C(37B)	119.6(4)
C(32B)-P(1B)-C(26B)	108.41(19)	C(37B)-C(32B)-P(1B)	119.8(3)
N(2B)-P(2B)-C(38B)	113.9(2)	C(32B)-C(33B)-H(33B)	120.0
N(2B)-P(2B)-C(44B)	111.9(2)	C(34B)-C(33B)-C(32B)	120.0(4)
N(2B)-P(2B)-C(50B)	107.33(19)	C(34B)-C(33B)-H(33B)	120.0
C(44B)-P(2B)-C(38B)	108.0(2)	C(33B)-C(34B)-H(34B)	119.8
C(50B)-P(2B)-C(38B)	108.1(2)	C(35B)-C(34B)-C(33B)	120.3(4)
C(50B)-P(2B)-C(44B)	107.3(2)	C(35B)-C(34B)-H(34B)	119.8
P(2B)-N(2B)-P(1B)	136.2(2)	C(34B)-C(35B)-H(35B)	119.9
C(21B)-C(20B)-P(1B)	120.4(3)	C(34B)-C(35B)-C(36B)	120.2(4)
C(21B)-C(20B)-C(25B)	119.2(4)	C(36B)-C(35B)-H(35B)	119.9
C(25B)-C(20B)-P(1B)	120.3(3)	C(35B)-C(36B)-H(36B)	119.7
C(20B)-C(21B)-H(21B)	119.9	C(35B)-C(36B)-C(37B)	120.7(4)
C(22B)-C(21B)-C(20B)	120.3(4)	C(37B)-C(36B)-H(36B)	119.7
C(22B)-C(21B)-H(21B)	119.9	C(32B)-C(37B)-H(37B)	120.4
C(21B)-C(22B)-H(22B)	120.3	C(36B)-C(37B)-C(32B)	119.3(4)
C(23B)-C(22B)-C(21B)	119.5(4)	C(36B)-C(37B)-H(37B)	120.4
C(23B)-C(22B)-H(22B)	120.3	C(39B)-C(38B)-P(2B)	118.5(4)
C(22B)-C(23B)-H(23B)	119.5	C(39B)-C(38B)-C(43B)	117.5(5)
C(22B)-C(23B)-C(24B)	121.1(5)	C(43B)-C(38B)-P(2B)	123.4(4)
C(24B)-C(23B)-H(23B)	119.5	C(38B)-C(39B)-H(39B)	119.2
C(23B)-C(24B)-H(24B)	119.9	C(38B)-C(39B)-C(40B)	121.7(6)
C(23B)-C(24B)-C(25B)	120.2(5)	C(40B)-C(39B)-H(39B)	119.2
C(25B)-C(24B)-H(24B)	119.9	C(39B)-C(40B)-H(40B)	120.2
C(20B)-C(25B)-H(25B)	120.1	C(39B)-C(40B)-C(41B)	119.5(7)
C(24B)-C(25B)-C(20B)	119.7(4)	C(41B)-C(40B)-H(40B)	120.2
C(24B)-C(25B)-H(25B)	120.1	C(40B)-C(41B)-H(41B)	120.5
C(27B)-C(26B)-P(1B)	121.1(4)	C(42B)-C(41B)-C(40B)	119.0(6)
C(31B)-C(26B)-P(1B)	119.8(4)	C(42B)-C(41B)-H(41B)	120.5
C(31B)-C(26B)-C(27B)	119.0(5)	C(41B)-C(42B)-H(42B)	119.5
C(26B)-C(27B)-H(27B)	119.6	C(41B)-C(42B)-C(43B)	121.0(6)
C(28B)-C(27B)-C(26B)	120.7(6)	C(43B)-C(42B)-H(42B)	119.5
C(28B)-C(27B)-H(27B)	119.6	C(38B)-C(43B)-H(43B)	119.7
C(27B)-C(28B)-H(28B)	120.4	C(42B)-C(43B)-C(38B)	120.6(5)
C(29B)-C(28B)-C(27B)	119.3(7)	C(42B)-C(43B)-H(43B)	119.7
C(29B)-C(28B)-H(28B)	120.4	C(45B)-C(44B)-P(2B)	121.9(3)

C(49B)-C(44B)-P(2B)	121.7(4)	C(51C)-C(52C)-H(52C)	124.2
C(49B)-C(44B)-C(45B)	116.4(4)	C(53B)-C(54C)-H(54C)	123.3
C(44B)-C(45B)-H(45B)	119.3	C(53B)-C(54C)-C(55C)	113.5(14)
C(46B)-C(45B)-C(44B)	121.3(5)	C(55C)-C(54C)-H(54C)	123.3
C(46B)-C(45B)-H(45B)	119.3	C(50B)-C(55C)-C(54C)	126.5(15)
C(45B)-C(46B)-H(46B)	119.8	C(50B)-C(55C)-H(55C)	116.8
C(47B)-C(46B)-C(45B)	120.4(5)	C(54C)-C(55C)-H(55C)	116.8
C(47B)-C(46B)-H(46B)	119.8	N(2A)-P(1A)-C(20A)	110.2(2)
C(46B)-C(47B)-H(47B)	119.9	N(2A)-P(1A)-C(26A)	108.71(18)
C(46B)-C(47B)-C(48B)	120.1(5)	N(2A)-P(1A)-C(32A)	114.63(19)
C(48B)-C(47B)-H(47B)	119.9	C(20A)-P(1A)-C(32A)	107.1(2)
C(47B)-C(48B)-H(48B)	120.0	C(26A)-P(1A)-C(20A)	108.6(2)
C(47B)-C(48B)-C(49B)	120.0(5)	C(26A)-P(1A)-C(32A)	107.4(2)
C(49B)-C(48B)-H(48B)	120.0	N(2A)-P(2A)-C(38A)	115.35(17)
C(44B)-C(49B)-C(48B)	121.7(5)	N(2A)-P(2A)-C(44A)	111.13(18)
C(44B)-C(49B)-H(49B)	119.1	N(2A)-P(2A)-C(50A)	107.83(18)
C(48B)-C(49B)-H(49B)	119.1	C(38A)-P(2A)-C(44A)	108.26(17)
C(51B)-C(50B)-P(2B)	118.6(5)	C(38A)-P(2A)-C(50A)	106.78(17)
C(51B)-C(50B)-C(55B)	121.6(6)	C(50A)-P(2A)-C(44A)	107.11(17)
C(55B)-C(50B)-P(2B)	119.8(4)	P(2A)-N(2A)-P(1A)	139.7(2)
C(51C)-C(50B)-P(2B)	122.1(9)	C(21A)-C(20A)-P(1A)	120.3(4)
C(55C)-C(50B)-P(2B)	124.2(7)	C(21A)-C(20A)-C(25A)	119.9(4)
C(55C)-C(50B)-C(51C)	110.4(12)	C(25A)-C(20A)-P(1A)	119.8(4)
C(52B)-C(53B)-H(53B)	120.5	C(20A)-C(21A)-H(21A)	120.2
C(52B)-C(53B)-C(54B)	118.9(6)	C(20A)-C(21A)-C(22A)	119.5(5)
C(54B)-C(53B)-H(53B)	120.5	C(22A)-C(21A)-H(21A)	120.2
C(52C)-C(53B)-H(53A)	116.7	C(21A)-C(22A)-H(22A)	120.0
C(54C)-C(53B)-H(53A)	116.7	C(23A)-C(22A)-C(21A)	120.0(5)
C(54C)-C(53B)-C(52C)	126.6(14)	C(23A)-C(22A)-H(22A)	120.0
C(50B)-C(51B)-H(51B)	119.5	C(22A)-C(23A)-H(23A)	119.5
C(50B)-C(51B)-C(52B)	121.0(8)	C(24A)-C(23A)-C(22A)	121.0(5)
C(52B)-C(51B)-H(51B)	119.5	C(24A)-C(23A)-H(23A)	119.5
C(53B)-C(52B)-C(51B)	121.6(8)	C(23A)-C(24A)-H(24A)	120.0
C(53B)-C(52B)-H(52B)	119.2	C(23A)-C(24A)-C(25A)	120.0(6)
C(51B)-C(52B)-H(52B)	119.2	C(25A)-C(24A)-H(24A)	120.0
C(53B)-C(54B)-H(54B)	120.9	C(20A)-C(25A)-H(25A)	120.2
C(55B)-C(54B)-C(53B)	118.1(7)	C(24A)-C(25A)-C(20A)	119.6(6)
C(55B)-C(54B)-H(54B)	120.9	C(24A)-C(25A)-H(25A)	120.2
C(50B)-C(55B)-H(55B)	120.7	C(27A)-C(26A)-P(1A)	121.7(3)
C(54B)-C(55B)-C(50B)	118.5(6)	C(27A)-C(26A)-C(31A)	119.4(4)
C(54B)-C(55B)-H(55B)	120.7	C(31A)-C(26A)-P(1A)	118.9(3)
C(50B)-C(51C)-H(51C)	117.8	C(26A)-C(27A)-H(27A)	120.0
C(52C)-C(51C)-C(50B)	124.4(19)	C(28A)-C(27A)-C(26A)	120.0(5)
C(52C)-C(51C)-H(51C)	117.8	C(28A)-C(27A)-H(27A)	120.0
C(53B)-C(52C)-C(51C)	112(2)	C(27A)-C(28A)-H(28A)	120.0
C(53B)-C(52C)-H(52C)	124.2	C(29A)-C(28A)-C(27A)	119.9(5)

C(29A)-C(28A)-H(28A)	120.0
C(28A)-C(29A)-H(29A)	119.8
C(28A)-C(29A)-C(30A)	120.4(5)
C(30A)-C(29A)-H(29A)	119.8
C(29A)-C(30A)-H(30A)	120.0
C(31A)-C(30A)-C(29A)	120.1(5)
C(31A)-C(30A)-H(30A)	120.0
C(26A)-C(31A)-H(31A)	119.9
C(30A)-C(31A)-C(26A)	120.1(4)
C(30A)-C(31A)-H(31A)	119.9
C(33A)-C(32A)-P(1A)	121.5(5)
C(33A)-C(32A)-C(37A)	119.7(5)
C(37A)-C(32A)-P(1A)	118.7(4)
C(32A)-C(33A)-H(33A)	120.0
C(32A)-C(33A)-C(34A)	119.9(7)
C(34A)-C(33A)-H(33A)	120.0
C(33A)-C(34A)-H(34A)	120.7
C(35A)-C(34A)-C(33A)	118.7(7)
C(35A)-C(34A)-H(34A)	120.7
C(34A)-C(35A)-H(35A)	119.1
C(36A)-C(35A)-C(34A)	121.7(6)
C(36A)-C(35A)-H(35A)	119.1
C(35A)-C(36A)-H(36A)	120.0
C(35A)-C(36A)-C(37A)	120.0(7)
C(37A)-C(36A)-H(36A)	120.0
C(32A)-C(37A)-H(37A)	120.0
C(36A)-C(37A)-C(32A)	119.9(6)
C(36A)-C(37A)-H(37A)	120.0
C(39A)-C(38A)-P(2A)	118.1(3)
C(43A)-C(38A)-P(2A)	122.5(3)
C(43A)-C(38A)-C(39A)	119.3(3)
C(38A)-C(39A)-H(39A)	119.8
C(40A)-C(39A)-C(38A)	120.5(4)
C(40A)-C(39A)-H(39A)	119.8
C(39A)-C(40A)-H(40A)	120.1
C(41A)-C(40A)-C(39A)	119.7(4)
C(41A)-C(40A)-H(40A)	120.1
C(40A)-C(41A)-H(41A)	119.8
C(42A)-C(41A)-C(40A)	120.4(4)
C(42A)-C(41A)-H(41A)	119.8
C(41A)-C(42A)-H(42A)	119.9
C(41A)-C(42A)-C(43A)	120.3(4)
C(43A)-C(42A)-H(42A)	119.9
C(38A)-C(43A)-C(42A)	119.8(4)
C(38A)-C(43A)-H(43A)	120.1
C(42A)-C(43A)-H(43A)	120.1
C(45A)-C(44A)-P(2A)	119.3(3)
C(45A)-C(44A)-C(49A)	119.7(4)
C(49A)-C(44A)-P(2A)	121.0(3)
C(44A)-C(45A)-H(45A)	120.3
C(44A)-C(45A)-C(46A)	119.4(4)
C(46A)-C(45A)-H(45A)	120.3
C(45A)-C(46A)-H(46A)	120.0
C(47A)-C(46A)-C(45A)	119.9(4)
C(47A)-C(46A)-H(46A)	120.0
C(46A)-C(47A)-H(47A)	119.7
C(46A)-C(47A)-C(48A)	120.7(4)
C(48A)-C(47A)-H(47A)	119.7
C(47A)-C(48A)-H(48A)	119.7
C(49A)-C(48A)-C(47A)	120.5(5)
C(49A)-C(48A)-H(48A)	119.7
C(44A)-C(49A)-H(49A)	120.1
C(48A)-C(49A)-C(44A)	119.8(4)
C(48A)-C(49A)-H(49A)	120.1
C(51A)-C(50A)-P(2A)	119.4(3)
C(55A)-C(50A)-P(2A)	120.9(3)
C(55A)-C(50A)-C(51A)	119.6(4)
C(50A)-C(51A)-H(51A)	119.9
C(52A)-C(51A)-C(50A)	120.1(4)
C(52A)-C(51A)-H(51A)	119.9
C(51A)-C(52A)-H(52A)	120.0
C(53A)-C(52A)-C(51A)	120.0(4)
C(53A)-C(52A)-H(52A)	120.0
C(52A)-C(53A)-H(53C)	119.7
C(54A)-C(53A)-C(52A)	120.6(4)
C(54A)-C(53A)-H(53C)	119.7
C(53A)-C(54A)-H(54A)	119.9
C(53A)-C(54A)-C(55A)	120.2(4)
C(55A)-C(54A)-H(54A)	119.9
C(50A)-C(55A)-C(54A)	119.4(4)
C(50A)-C(55A)-H(55A)	120.3
C(54A)-C(55A)-H(55A)	120.3
Cl(1)-C(56)-Cl(2A)	118.6(5)
Cl(1)-C(56)-Cl(2B)	104.1(6)
Cl(1)-C(56)-H(56C)	110.9
Cl(1)-C(56)-H(56D)	110.9
Cl(1)-C(56)-H(56A)	107.7
Cl(1)-C(56)-H(56B)	107.7
Cl(2A)-C(56)-H(56A)	107.7
Cl(2A)-C(56)-H(56B)	107.7
Cl(2B)-C(56)-H(56C)	110.9
Cl(2B)-C(56)-H(56D)	110.9

H(56C)-C(56)-H(56D)	108.9	Cl(5)-C(58)-H(58B)	105.7
H(56A)-C(56)-H(56B)	107.1	Cl(6)-C(58)-H(58A)	105.7
Cl(3A)-C(57A)-H(57A)	109.4	Cl(6)-C(58)-H(58B)	105.7
Cl(3A)-C(57A)-H(57B)	109.4	H(58A)-C(58)-H(58B)	106.2
Cl(4A)-C(57A)-Cl(3A)	111.1(7)	Cl(3B)-C(57B)-H(57C)	111.9
Cl(4A)-C(57A)-H(57A)	109.4	Cl(3B)-C(57B)-H(57D)	111.9
Cl(4A)-C(57A)-H(57B)	109.4	Cl(4B)-C(57B)-Cl(3B)	99.5(15)
H(57A)-C(57A)-H(57B)	108.0	Cl(4B)-C(57B)-H(57C)	111.9
Cl(5)-C(58)-Cl(6)	126.3(13)	Cl(4B)-C(57B)-H(57D)	111.9
Cl(5)-C(58)-H(58A)	105.7	H(57C)-C(57B)-H(57D)	109.6

4. Laser Experiment: Flash Quench Electron Transfer and Rate Derivation:

Laser samples were composed of 20 µM $[Ru(bpy)_3]Cl_2$, 10 mM of oxidative quencher (methylviologen-2 PF_6) and different concentrations of $(PPN)_4[Fe(CN\text{-}B(C_6F_5)_3)_6]$ (0, 0.22, 0.5 and 1 mM) in 2 mL of acetonitrile. Laser samples were prepared in the box and placed in a quartz fluorescence cuvette with a high-vacuum Teflon valve, equipped with a small stir bar.

For nanosecond-to-millisecond transient luminescence and absorption experiments, excitation was provided by 460 nm pulses from a tunable optical parametric oscillator (Spectra Physics, Quanta-Ray MOPO-700) pumped by the third harmonic from a Spectra Physics Q-switched Nd:YAG laser (Spectra-Physics, Quanta-Ray PRO-Series, 8 ns pulse width) operated at 10 Hz. Probe light was provided by a 75-W arc lamp (PTI model A 1010) that could be operated in continuous or pulsed mode and passed through the sample collinearly with the excitation pulse. After rejection of scattered light by appropriate long- and short-pass filters, and intensity modulation by a neutral density filter, probe wavelengths were selected by a double monochromator (Instruments SA DH-10) with 1 mm slits. Transmitted light was detected by a photomultiplier tube (PMT, Hamamatsu R928) and amplified by a 200 MHz wideband voltage amplifier DHPVA-200 (FEMTO). Luminescence decays were monitored at 630 nm. Single wavelength transient absorption kinetics were monitored at 440 nm, averaging ~500 shots. Data from three separate time scales (2 µs, 10 µs and100 µs) were collected,

log-compressed, and spliced together to produce full kinetics traces using Matlab software (Mathworks).

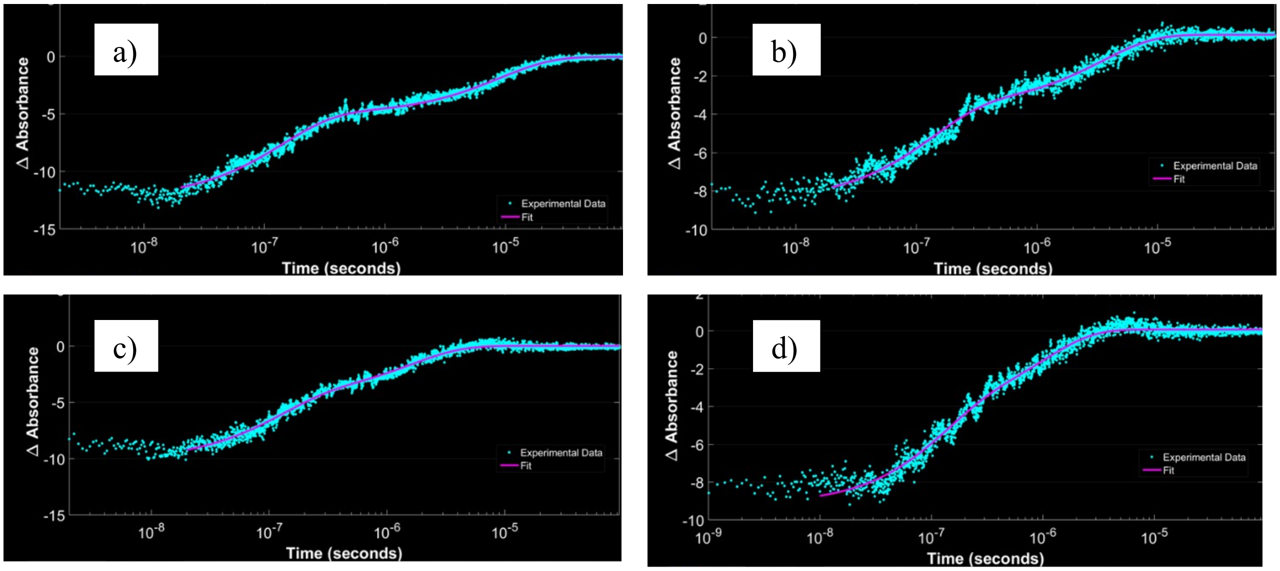

Figure S14. Transient absorption kinetics following reductive quenching of $[Ru(bpy)_3]^{3+}$ (λ_{ex} = 460 nm; λ_{obsd} = 440 nm) at different concentration of complex **3**: a) without complex **3**, b) 0.22 mM, c) 0.5 mM, and d) 1 mM of complex **3**.

Table S8. Rate constants of the reductive quenching of Ru(III) with different concentration of **3** estimated from the MatLab modeling.

**Luminescence**	**Concentration (µM)**	**k_{rate} (M^{-1} s^{-1})**	**Lifetime (s)**
No Fe	0	7.16E+06	1.40E-07
Fe-220uM	220	7.64E+06	1.31E-07
Fe-500uM	500	7.91E+06	1.26E-07
Fe-1mM	1000	8.40E+06	1.19E-07

**Transient Absorption**			
No Fe	0	7.16E+06	1.40E-07
		1.12E+05	8.95E-06
Fe-220uM	220	7.64E+06	1.31E-07
		2.90E+05	3.45E-06
Fe-500uM	500	7.91E+06	1.26E-07
		6.09E+05	1.64E-06
Fe-1mM	1000	8.40E+06	1.19E-07
		9.57E+05	1.05E-06

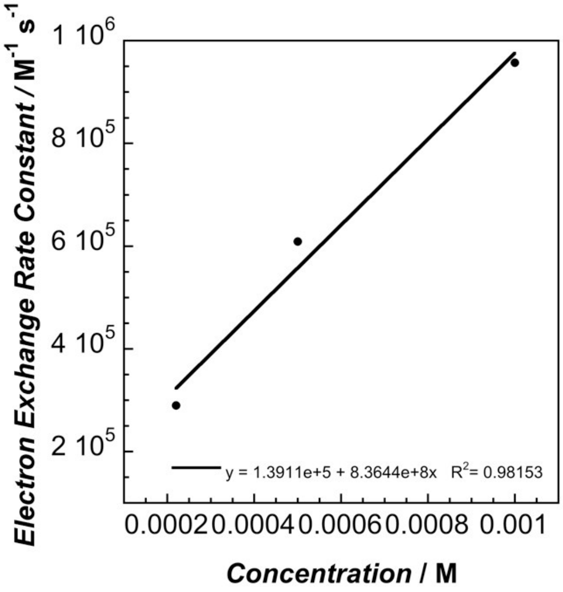

Figure S15. Linear fit of observed electron exchange constant versus concentration of **3**.

5. UV-Vis Data

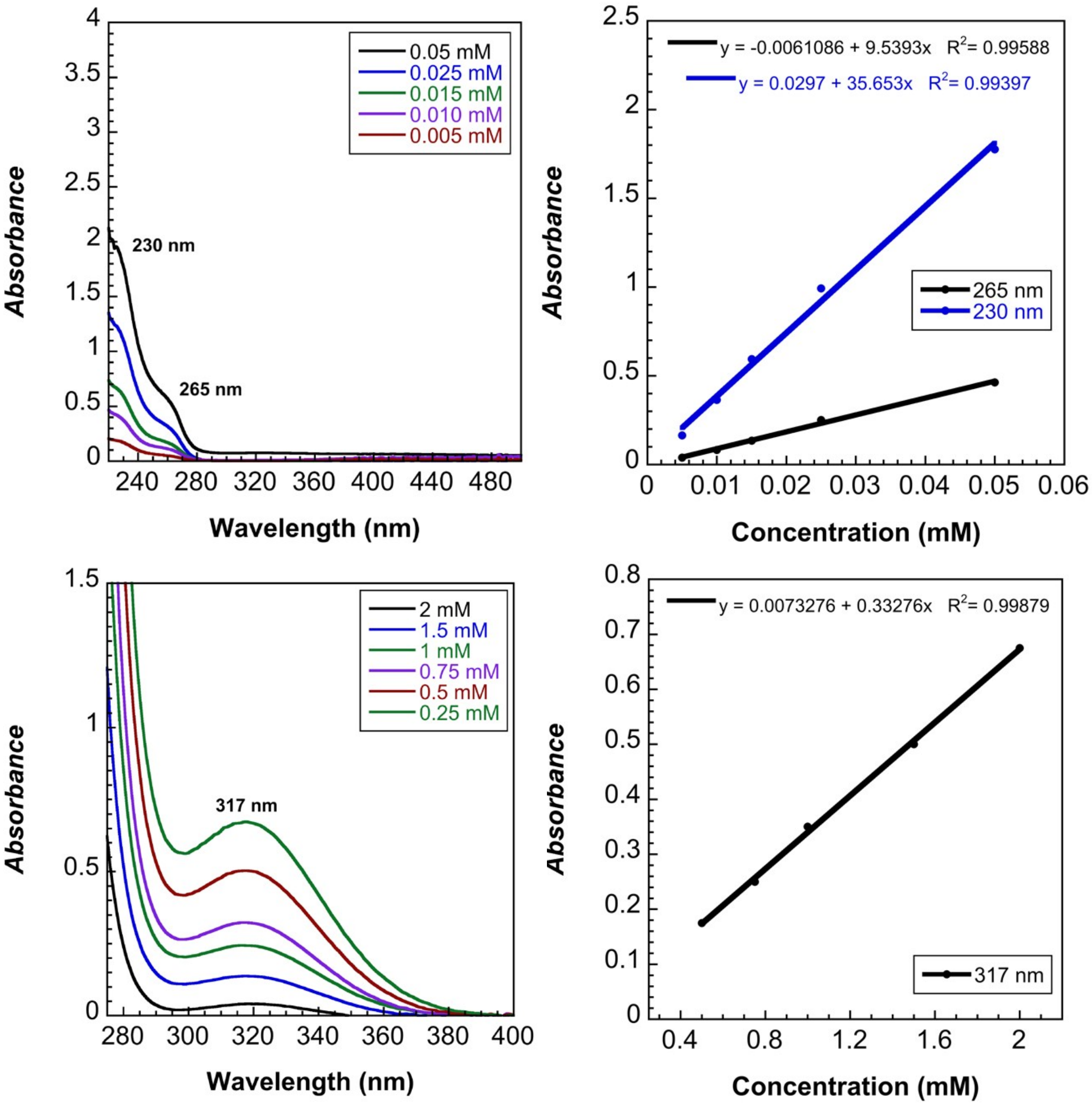

Figure S16. **Top Left**: UV-vis spectra of **3** in dichloromethane , displaying the two π to π* transitions of the borane moieties. **Top Right**: Linear fit of absorbance versus concentration for the two π to π* transitions. **Bottom Left**: UV-vis spectra of the $^1A_{1g} \rightarrow {}^1T_{1g}$ transition for **3**. **Bottom Right**: Linear fit of absorbance versus concentration for the $^1A_{1g} \rightarrow {}^1T_{1g}$ transition for **3**.

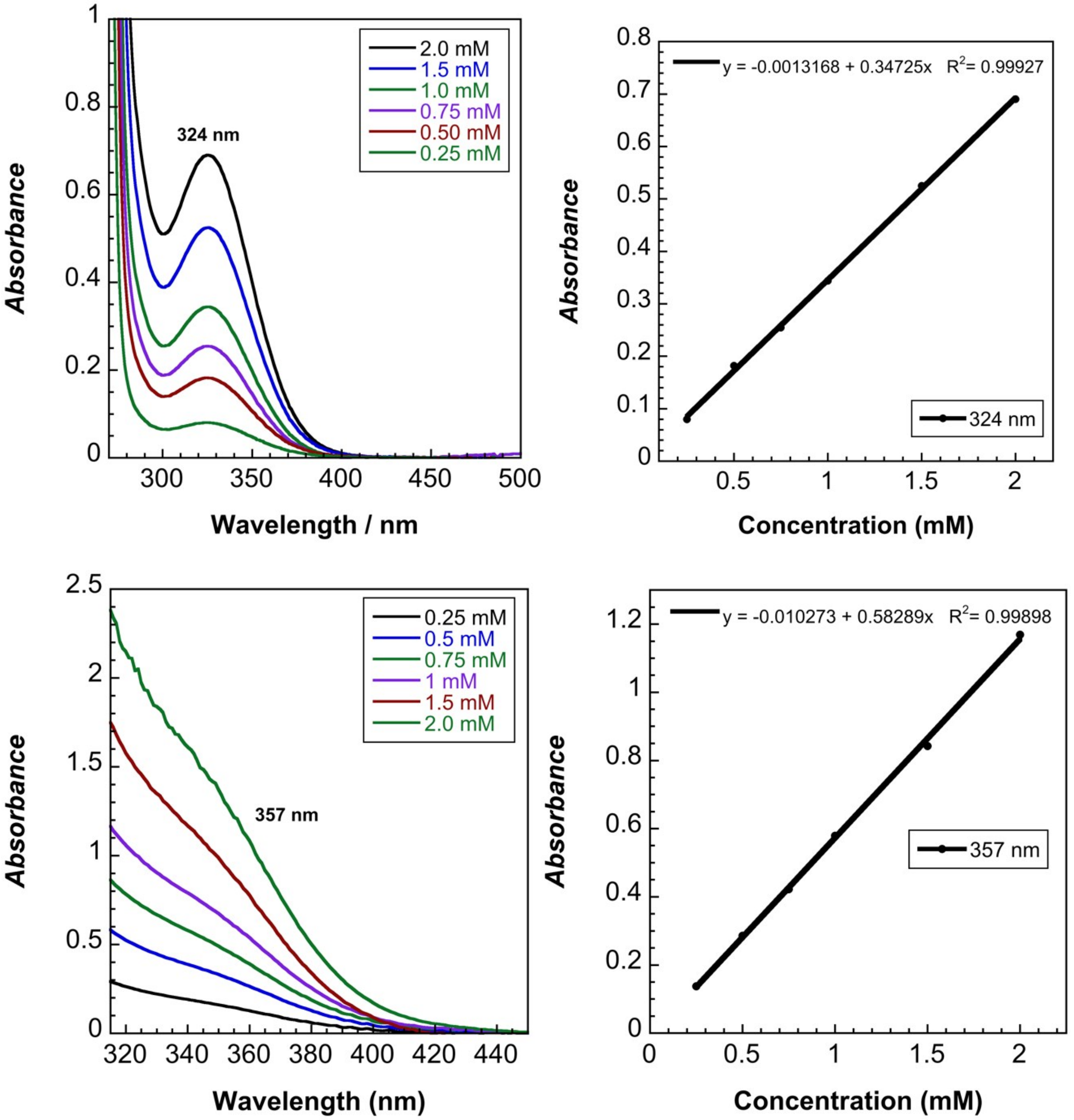

Figure S17. **Top Left**: UV-vis spectra of the $^1A_{1g} \rightarrow\ ^1T_{1g}$ transition for **2**. **Top Right**: Linear fit of absorbance versus concentration for the $^1A_{1g} \rightarrow\ ^1T_{1g}$ transition for **2**. **Bottom Left**: UV-vis spectra of the $^1A_{1g} \rightarrow\ ^1T_{1g}$ transition for **1a**. **Bottom Right**: Linear fit of absorbance versus concentration for the $^1A_{1g} \rightarrow\ ^1T_{1g}$ transition for **1a**.

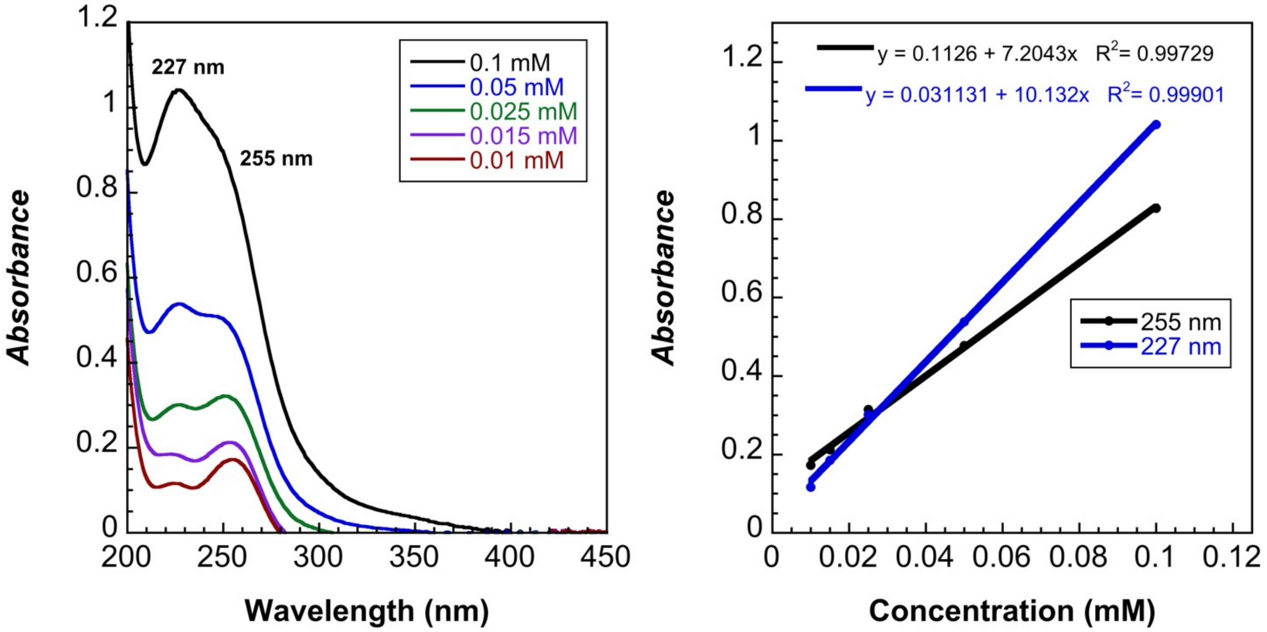

Figure S18. **Left**: UV-vis spectra of MLCT transitions for **1a**. **Right**: Linear fit of absorbance versus concentration for the MLCT transitions for **1a**.

References:

1. Barzilay, C. M.; Sibilia, S. A.; Spiro, T. G.; Gross, Z. *Chem. Eur. J.* **1995**, *1*, 223-231.
2. Alexander, J. J.; Gray, H. B. *J. Am. Chem. Soc.* **1968**, *90*, 4260-4271.
3. Berret, R. R., Fitzsimmons, B. W. *J. Chem. Soc. (A): Inorg. Phys. Theor***. 1967**, 525-527.
4. Sheldrick, G. M. *Acta Cryst.* **1990**, A46, 467-473.
5. Sheldrick, G. M. *Acta Cryst.* **2015**, C71, 3-8
6. Müller, P. *Crystallography Reviews* **2009**, *15*, 57-83.
